# Balancing LncRNA H19 and miR‐675 Bioconversion as a Key Regulator of Embryonic Myogenesis Under Maternal Obesity

**DOI:** 10.1002/jcsm.13791

**Published:** 2025-03-31

**Authors:** Yao Gao, Md Nazmul Hossain, Liang Zhao, Xiangdong Liu, Yanting Chen, Jeanene Marie Deavila, Mei‐Jun Zhu, Gordon K. Murdoch, Min Du

**Affiliations:** ^1^ Nutrigenomics and Growth Biology Laboratory, Department of Animal Sciences Washington State University Pullman Washington USA; ^2^ College of Animal Science and Technology Nanjing Agricultural University Nanjing Jiangsu China; ^3^ Dana‐Farber Cancer Institute Boston Massachusetts USA; ^4^ School of Food Science Washington State University Pullman Washington USA

**Keywords:** embryonic myogenesis, H19, KHSRP, maternal obesity, miR675

## Abstract

**Background:**

Maternal obesity (MO) impairs fetal skeletal muscle development, but the underlying mechanisms remain poorly defined. The regulatory roles of lncRNA *H19* and its first exon derived *microRNA675* (*miR675*) in prenatal muscle development remain to be examined. *H19*/*Igf2* are in the same imprinting cluster with *H19* expressed from the maternal allele while *Igf2* expresses paternally. *H19* contains a G‐rich loop, and KH‐type splicing regulatory protein (KHSRP) mediates the biogenesis of pre‐*miRNAs* containing G‐rich loops, which depends on its phosphorylation by AKT, a key mediator of IGF2 signalling. This study aims to depict the elusive function of these regulators that are affected by MO during embryonic myogenesis.

**Methods:**

Single‐cell transcriptomic sequencing and GeoMx spatial RNA sequencing were performed to identify the differentially expressed genes between embryos from MO and control (CT) mice. Both E11.5 and E13.5 embryos were collected and analysed to validate the sequencing data. The roles of *H19* and *miR657* in myogenesis were further analysed in P19 embryonic cells via CRISPR/dCas9‐mediated *H19* activation and inhibition. The epigenetic changes of *H19* were analysed by methylated DNA immunoprecipitation, and allele‐targeted analysis of *H19* was performed by crossing C57BL/6J and CAST/EiJ mice.

**Results:**

Transcriptomic analysis showed that MO embryos contained less differentiated myocytes (1.34%) than CT embryos (2.86%). Myogenesis‐related GO biological processes were down‐regulated in the MO embryonic myotome region. MO embryos showed lower expression of myogenic transcription factors such as *Myf5*, *Myod1*, *Myog*, *Mef2c* and *Myh3* (*p* < 0.05). MO altered epigenetic modifications of the *H19* genomic cluster, showing a decreased methylation level in *H19* imprinting control region (*p <* 0.05) and a diallelic expression pattern of *H19*, which elevated its expression in MO embryos. Overexpression of *H19* inhibited myogenesis in P19 cells, but *miR675* promoted myogenesis, suggesting the critical regulatory roles of bioconversion of *H19* to *miR675*. A KHSRP mediates the biogenesis of *miR675*, a process that relies on its phosphorylation by IGF2/AKT signalling. Knocking‐down of KHSRP and inhibition of AKT abolished *miR675* biogenesis. MO suppressed IGF2/AKT signalling and blocked KHSRP‐dependent *miR675* biogenesis in embryos.

**Conclusions:**

We found differential effects of *H19* and *miR675* on embryonic myogenesis. MO up‐regulates *H19* but blocks its *miR675* bioconversion via suppressing IGF2/AKT/KHSRP signalling axis. Myogenesis in MO embryos was impeded due to the highly accumulated *H19* and blocked *miR675* biogenesis.

## 1 Introduction

Currently, one‐third of the global population is either obese or overweight, and more than 40% of women at the reproductive age are obese in the United States [1]. Maternal obesity (MO) during pregnancy induces permanent alterations in fetal development, encompassing changes in cell composition, organ structure and disease susceptibility, which lead to metabolic disorders in the later life of children [2]. Skeletal muscle, as a critical metabolic organ, accounts for ~35% of total body mass [S1]. MO leads to persistent dysfunction of offspring muscle, signifying the importance of early muscle development on the long‐term function of muscle [3]. However, the exact mechanisms underlying this phenomenon remain poorly defined.

Myogenesis is regulated by a group of myogenic regulatory factors: MYF5, MYOD, MYOG and MRF4. Their spatiotemporal expression governs embryonic myogenesis [4]. In mice, myogenesis initiates on Embryonic Day 8.5 (E8.5). Following the formation of the three germ layers, a portion of embryonic pluripotent stem cells within the somitic mesoderm commit into myogenic progenitor cells, characterized by the expression of paired box transcription factors *Pax3* and *Pax7*. These myogenic progenitor cells subsequently express MYF5 and transform into myoblasts, which eventually differentiate into myotubes. The first wave of myotube formation (primary myogenesis) occurs from E8.5 to E14.5, serving as a scaffold for the subsequent secondary myogenesis and postnatal muscle growth [5]. Disruption of myogenesis at this stage results in lasting adverse effects on postnatal skeletal muscle growth [6].

Imprinted genes are evolutionarily selected for their critical roles in regulating fetal and placental development. The *H19*/*Igf2* imprinting cluster is the first identified, with *H19* maternally expressed and *Igf2* paternally expressed [7]. Both *H19* and *Igf2* are highly expressed in embryos [8]. An imprinting control region (ICR) between *Igf2* and *H19* governs the monoallelic expression of *H19*/*Igf2*. In the maternal allele, the ICR remains unmethylated, permitting the binding of CCCTC‐binding factor (CTCF), an insulator protein, to the ICR. This association directs an enhancer functioning towards the *H19* promoter, initiating its expression. Conversely, in the paternal allele, the ICR is hypermethylated, leading to chromatin remodelling that obstructs the CTCF binding. As a result, the enhancer interacts with the *Igf2* promoter to enhance its expression [9]. The genotype and epigenotype of *H19*/*Igf2* locus accounts for 31% newborn weight variance [10], showing the critical roles of this locus in regulating prenatal development.

A previous study reported that *H19* lncRNA inhibits muscle development by recruiting a KH‐type splicing regulatory protein (KHSRP) to degrade myogenic mRNAs [11]. These mRNAs usually contain adenylate/uridylate‐rich elements (AREs) in their 3′ untranslated region, which can be recognized by KHSRP [12]. However, this inhibitory role of KHSRP in myogenesis is abolished when a serine (S193) within the KH1 domain of KHSRP is phosphorylated by AKT [13]. Phosphorylation triggers the dissociation of KHSRP from mRNAs, promoting its function shift from mRNA decay to microRNAs (miRs) biogenesis [12]. Consequently, myogenic mRNAs are stabilized, and phosphorylated KHSRP (p‐KHSRP) is repurposed to promote the biogenesis of miRs. In addition, KHSRP preferentially binds to the GC‐rich terminal loop of the primary‐miRs (pri‐miRs) and precursor‐miRs (pre‐miRs) [14]. As a long non‐coding RNA (lncRNA), *H19* harbours an intragenic *miR675* [15], which was discovered to be a promyogenic miR, partially via inhibition of BMP signalling [16]. Though KHSRP has been proven to regulate maturation of myogenic miRs during muscle regeneration [12], its roles in mediating *miR675* biogenesis from *H19* and its effects on embryonic myogenesis remain unexamined.

IGF2 is a key embryonic growth factor promoting muscle development [17]. Because IGF2 is a major growth factor activating AKT signalling in fetal skeletal muscle, which regulates KHSRP phosphorylation, and *H19* and *Igf2* are in the same imprinting cluster, this raises an intriguing question about their roles in mediating KHSRP‐mediated miR biogenesis and embryonic myogenesis impaired due to MO.

In this study, we observed that MO reduced IGF2/AKT signalling, which decreased p‐KHSRP‐mediated processing of *H19* into its derived *miR675*, suppressing embryonic myogenesis. Mechanistically, we found that MO elevated HIF1α signalling, which increased *H19* expression and disrupted the imprinting pattern of the *Igf2*/*H19* locus, attenuating IGF2AKT signalling and p‐KHSRP‐mediated bioconversion of *H19* into *miR675*. These data suggest that the *H19*/*Igf2* imprinting cluster is a key intervention target to improve embryonic muscle development impaired by MO.

## 2 Materials and Methods

### 2.1 Ethics Declarations

All animal studies were conducted in AAALAC‐approved facilities under the guideline of the Institutional Animal Use and Care Committee at Washington State University (Permit No. 6712.04) in accordance with the standards of the 1964 Declaration of Helsinki and its later amendments.

### 2.2 Animals

A total of 32 female C57BL/6J mice (8 weeks old; 000664, The Jackson Lab, Bar Harbor, Maine) were randomly assigned to MO (*n* = 16) and control (CT, *n* = 16) groups per established protocol [S2]. To induce obesity, mice in MO group were fed with high‐fat diet (HFD, 45% calories from fat; D12451, Research Diets, New Brunswick, NJ) for 2–3 months before mating. Mice in the CT group were fed with a control diet (10% calories from fat, D12450, Research Diets). Mice in the MO group that had 20% heavier body weight than the average body weight of CT mice were considered obese (Figure S1a,b). The mating was confirmed by the presence of vaginal plug in the following early morning and designated as E0.5. After mating, pregnant mice were continually fed with their respective diets until embryo collection at E11.5 and E13.5. During embryo collection, embryos from the same group showed good uniformity, and their embryonic stages were further confirmed by somite numbers and morphology [S3, S4]. No embryos were excluded from the sample pooling and subsequent data analysis. All mice were housed at 22°C on 12‐h light/12‐h dark cycles, fed *ad libitum*. The food intake and body weight were measured regularly (Figure S1c,d). C57BL/6J male mice (4 months old) fed by a regular chow were used to mate with these females. At necropsy, the white adipose tissues of maternal mice were weighed (Figure S1e).

### 2.3 Embryo and Sample Collection

Embryos for the purpose of single‐cell sequencing were collected as follows: Pregnant mice were anaesthetised by carbon dioxide inhalation and euthanized by cervical dislocation at E11.5 and E13.5. Embryos from CT and MO groups were alternatively sampled to eliminate the time difference during collection. The cranial portion of the embryos were removed under a stereomicroscope. Each litter from one pregnant mouse was regarded as one biological replicate.

For spatial RNA sequencing, E13.5 embryo samples were processed per the standard protocol of GeoMx DSP, Nanostring [S5]. A total of six cross‐sections from CT embryos (*n* = 6) and six cross‐sections from HFD embryos at E13.5 were selected. Each randomly selected embryo from one pregnant mouse was regarded as one biological replicate (*n* = 6). Based on the MYF5‐positive staining (PA5‐47565, Thermo Fisher, Waltham, MA), myotomes, the regions of interest (ROIs), were manually selected to construct the transcriptomic library. A total of six libraries from ROIs of CT embryos and four libraries from ROIs of HFD embryos were successfully established. The cleaved indices were sequenced in an Illumina NextSeq 2K (San Diego, CA) and quantified to generate digital quantification of RNA expression with a spatial context.

### 2.4 Single‐Cell RNA Sequencing and Spatial RNA Sequencing

To obtain single‐cell suspension, we followed the same protocol as described previously [S6]. Briefly, 18 HFD embryos (three embryos/HFD mouse, *n* = 6) and 18 CT embryos (three embryos/CT mouse, *n* = 6) were randomly chosen for each time point (E11.5 and E13.5). Each group was further subdivided into two samples to avoid the batch effects. Embryos were dissociated into single‐cell suspension by TryLE Express dissociation reagent (12604013, Thermo Fisher). The cell numbers were counted using a haemocytometer, and the cell viability of more than 90% was confirmed using Trypan blue (15250061, Thermo Fisher) exclusion staining. A total of four cDNA libraries (two CT and two HFD) were constructed and multiplexed using a Chromium Next GEM Single Cell 3′ Kit v3.1 (1000269, 10x Genomics, Pleasanton, CA) and sequenced (PE150) by NovaSeq 6000 (Illumina Inc., San Diego, CA) platform at Novogene (Sacramento, CA). All the remaining embryos were labelled and frozen at −80°C until further analysis.

For single‐cell RNA sequencing (scRNA‐seq) data analysis, the raw sequencing data from both E11.5 and E13.5 were assembled and aggregated using the CellRanger (v7.0.0, 10x Genomics), with mouse transcriptome as reference (mm10‐2020‐A). The data were then integrated into R and analysed following the Seurat package (v4.3.0.1) tutorial [S7]. Detailed data processing has been described previously [S2]. Briefly, raw data were filtered (UMI > 200, MT < 30, COUNT < 12 500), normalized (LogNormalize) and scaled before generating the uniform manifold approximation and projection (UMAP) graph (resolution 0.2). The cells were clustered into 21 groups. The marker genes for each cluster were generated (FindAllMarkers) and annotated (Figure S2). Differentially expressed gene (DEG) analysis was conducted to find the DEG profile between CT and HFD groups (FindMarkers). The significance was confirmed using the MAST test with Bonferroni adjusted *p* value < 0.05. Based on Y‐chromosome gene expression, cells were separated into males and females in single‐cell RNA‐seq data, which showed no significant difference due to MO. Thus, data from male and female cells were aggregated for downstream analysis. The myogenic cluster was selected to perform Gene Ontology (GO) analysis (R package, cluster Pro‐filer v3.18.0). All detected genes from the whole scRNA‐seq library were used as background. Terms were enriched with the nominal *p* value < 0.05 and false discovery rate (*q* value < 0.05). Pseudotiming (Monocle3 v1.2.7) was performed to analyse the cell trajectory within the myogenic subpopulation.

For spatial RNA sequencing data analysis, the raw sequencing data were assembled and transferred into .DCC file (digital conversation counts) in Illumina Base Space analysis platform. The preprocessed .DCC data file was integrated into R and analysed following the GeoMx Workflows (v3.6.0) Vignette [S8]. Briefly, GeoMx Mouse Whole Transcriptome Atlas (Mm_R_NGS_WTA_v1.0.pkc) was used to configurate the raw data. The data were filtered (minSegmentReads = 10, percentTrimmed = 80, percentStitched = 80, percentAligned = 80, percentSaturation = 10, minNegativeCount = 1, maxNTCCount = 9000, minNuclei = 10, minArea = 100) and normalized (norm_method = ‘quant’) before generating the UMAP graph. DEG analysis was conducted to find the DEG profile between CT and MO groups (mixedModelDE). The significance was confirmed using the linear mixed‐effect model with *p* value < 0.05. The GO analysis was performed using R package, cluster Pro‐filer v3.18.0. All data were visualized and analysed by Prism GraphPad (V.7.0, San Diego, CA) and R package ggplot2 (v3.0.0), presented as mean ± SD.

### 2.5 Availability of Data

The data that support the findings of this study are available in the Supporting Information. The raw and processed data for E11.5 and E13.5 single‐cell RNA sequencing are accessible under accession number GSE27863 in the GEO database and are publicly available as of the date of publication. The raw and processed data for the GeoMx spatial sequencing are available online: https://doi.org/10.5281/zenodo.13140203 (accessed on 31 July 2024).

### 2.6 Others

Detailed protocols of cell culture and treatments, immunoblotting, immunocytochemical staining, methylated DNA immunoprecipitation, immunoprecipitation, genes gain‐and‐loss function analysis, RNA quantification and *H19* allelic expression analysis are available in the Supporting Information.

### 2.7 Statistical Analysis

All data were visualized and analysed by Prism GraphPad (V.7.0, San Diego, CA) or R package ggplot2, presented as mean ± SD. For comparison between two conditions, significance (*p* < 0.05) was identified using two‐tailed unpaired Student's *t* test. One‐way ANOVA with Tukey's multiple comparisons tests were carried out for multiple comparisons. D'Agostino and Pearson normality test was applied to test the normality of the dataset. Levene's test was used to test the equal variances. Embryos from a single litter were pooled, and one litter was considered as an experimental unit. Microscopy experiments were performed at least three times with similar results obtained. Experiments for quantification were also carried out at least three times. The sample size was estimated by G*Power (v3.1.9.7). No samples or animals were excluded in this study. The investigator was not blinded to the group allocation during the experiment but relied on unbiased data collection from randomly allocated samples into different groups.

## 3 Results

### 3.1 MO Negatively Affects Embryonic Myogenesis

**FIGURE 1:**
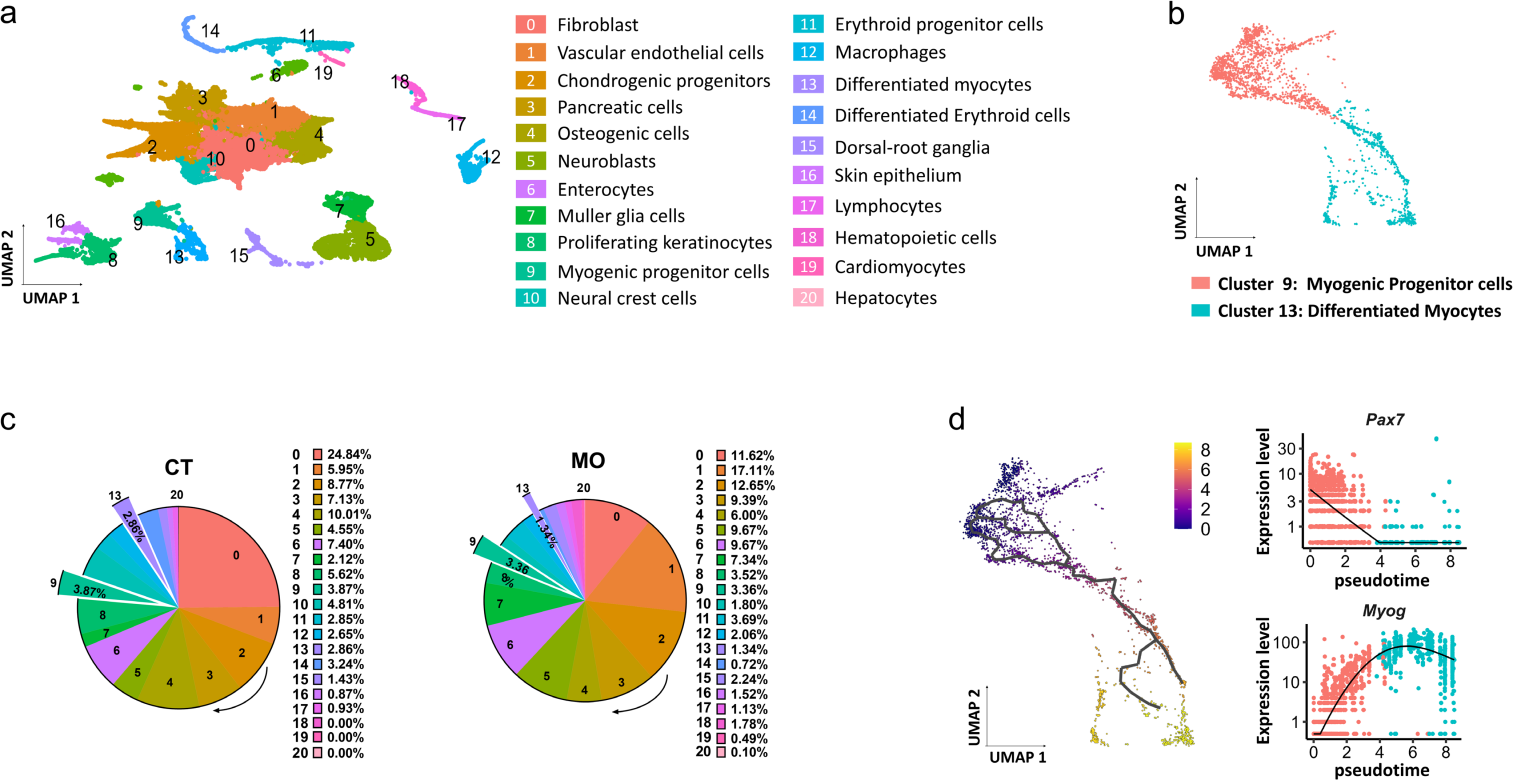
Single‐cell transcriptomic profiling of embryos. (a) Visualization of 21 cell clusters identified from integrated CT and MO datasets. (b) Visualization of two myogenic cell clusters. (c) Proportion of myogenic cells in embryonic cell pool. (d) Pseudo–time trajectory analysis of myogenic cells. Numbers 0–8, differentiation trajectory. All data were collected from integrated single‐cell samples of E11.5 and E13.5 embryos. CT, control; MO, maternal obesity; UMAP, uniform manifold approximation and projection.

Using unsupervised clustering of scRNA‐seq data integrated from E11.5 and E13.5 embryos (Figure 1a), a total of 20 cell groups were identified and annotated based on their marker gene expression (Figure S2). The myogenic cells were virtually sorted and further categorized into myogenic progenitors (Cluster 9) and differentiated myocytes (Cluster 13) based on their differentiation phases (Figure 1b). The proportion of myogenic progenitors in MO group (3.36%) was lower compared to the CT group (3.87%), and the proportion of differentiated myocytes (Cluster 13) in MO group (1.34%) was much lower compared to the CT group (2.86%) (Figure 1c).

Among the GO biological processes examined, genes involved in mRNA destabilization, response to decreased oxygen levels, BMP signalling, epigenetic regulation, genomic imprinting, chromatin remodelling and muscle cell apoptotic process were up‐regulated in MO group (Figure S3a). Conversely, genes involved in miRNA processing, protein localization, muscle development and cell growth were reduced in the MO group (Figure S3b). A pseudo–time trajectory of myogenesis was constructed for the myogenic cell cluster (Figures 1d and S4). As expected, expression patterns of myogenic genes accorded with their pseudodevelopmental trajectory. The early myogenic marker, *Pax7*, showed high expression initially and gradually decreased over time. Subsequently, *Myog* expression was initiated (Figure 1d).

**FIGURE 2:**
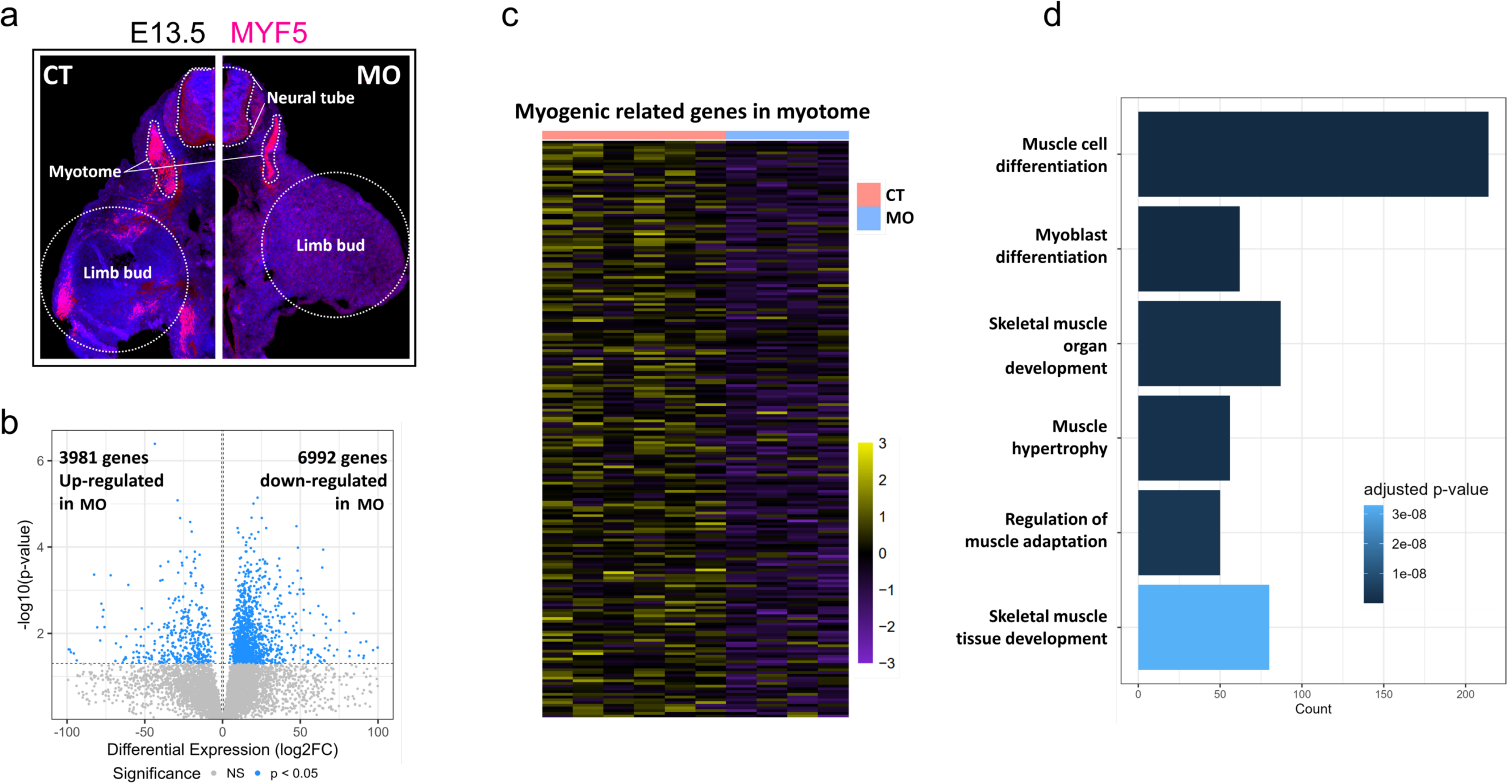
Spatial transcriptomic analysis of myotome in E13.5 embryos. (a) Immunohistochemical staining of the cross section of E13.5 embryonic hind limbs from CT and MO groups. (b) Volcano plot of DEGs up‐regulated and down‐regulated in MO E13.5 myotome compared with CT group. (c) The myogenic gene expression heatmap of CT and MO groups. (d) Down‐regulated myogenesis‐related GO biological processes terms in MO E13.5 myotome compared with CT group. CT, control; DEG, differentially expressed gene; MO, maternal obesity.

**FIGURE 3:**
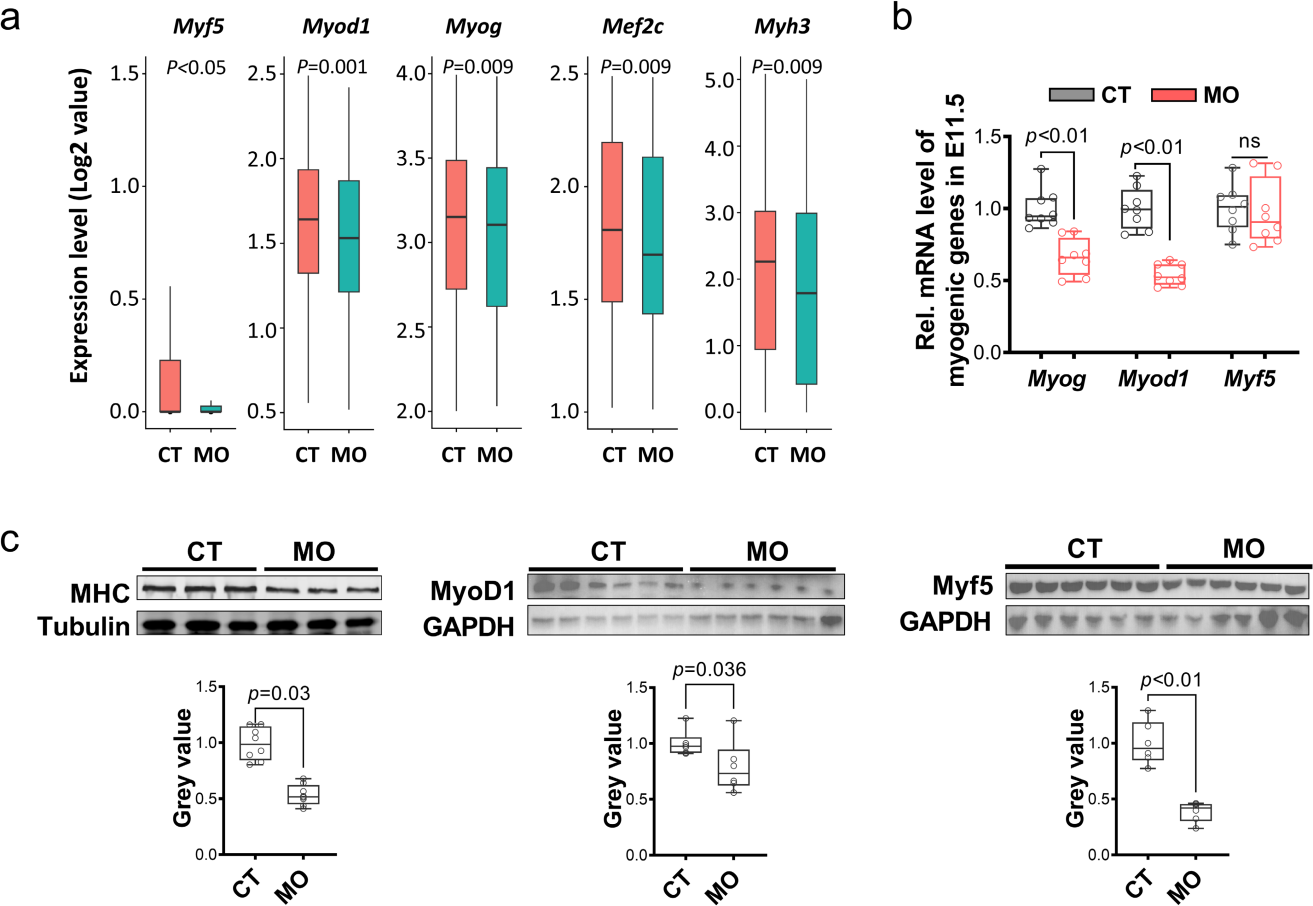
Maternal obesity inhibits myogenesis in embryos. (a) Expression levels of myogenic genes in scRNA‐seq data from myogenic cells cluster (E11.5 + E13.5). (b) Expression level of myogenic genes in E11.5 embryos (*n* = 8). (c) Representative images of western blots and quantification of MHC, MyoD1 and Myf5 protein levels in E11.5 embryos from CT and MO groups (*n* = 8). CT, control; MHC, myosin heavy chain; MO, maternal obesity; scRNA‐seq, single‐cell RNA sequencing.

Myotome is the origin of myogenic cells [5]. We specifically analysed the spatial transcriptome of myotome and found that MO inhibited *Myf5* expression (Figure 2a) and dramatically altered the transcriptional profile of this region, showing 3981 up‐regulated genes and 6992 down‐regulated genes in MO group (Figure 2b,c). Moreover, GO analysis demonstrated that myogenesis‐related biological processes were down‐regulated in the MO myotome (Figure 2d). Consistently, scRNA data showed decreased myogenic genes including *Myf5*, *Myod1*, *Myog*, *Mef2c* and *Myh3* in MO myogenic cluster (Figure 3a). The inhibited myogenesis was further confirmed in whole embryo samples, showing lower transcripts of *Myog* and *Myod1* and lower protein contents of MHC, MYF5 and MyoD1 in MO embryos (Figure 3b,c).

Overall, our scRNA‐seq and spatial transcriptomic data showed that maternal MO inhibits embryonic myogenesis.

#### 3.1.1 MO Induces Higher Expression of H19 but Lower Expression of miR675

**FIGURE 4:**
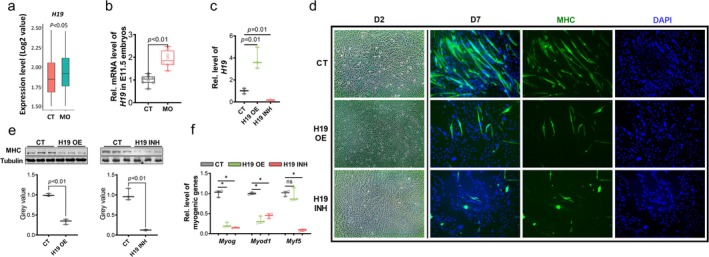
Overexpression and inhibition of *H19* suppress myogenesis in P19 cells. (a) Expression level of *H19* in scRNA‐seq data from myogenic cells cluster (E11.5 + E13.5). (b) Expression levels of *H19* in E11.5 embryos from CT and MO groups (*n* = 8). (c) Expression of *H19* lncRNA in P19 cells from CT, *H19* OE and *H19* INH groups (*n* = 3). (d) Representative images of ICC for MHC staining P19 cells (2‐ and 7‐day differentiations) from CT, *H19* OE and *H19* INH groups. (e) Representative images of western blots and quantification of MHC protein levels in P19 cells from CT, *H19* OE and *H19* INH groups (*n* = 3). (f) Myogenic gene expression in P19 cells from CT, *H19* OE and *H19* INH groups (*n* = 3). Data are presented as mean ± SD, each dot represents one cell culture dish; *p* values from two‐tailed unpaired Student's *t* test; asterisk (*) indicated *p* < 0.05. CT, control; D2, Day 2 differentiation; ICC, immunocytochemistry; INH, inhibition; MHC, myosin heavy chain; ns, not significant; OE, overexpression.

Based on scRNA‐seq, a moderate increase in the expression of *H19* lncRNA was observed as myogenesis progressed (Figure S4), with higher levels detected in the MO group (Figure 4a). Consistently, qPCR analysis revealed increased *H19* expression in the MO embryos (Figure 4b). To investigate the regulatory role of *H19* in myogenesis, we utilized the dCas9 system for *H19* overexpression and knockdown. By recruiting activators (VP64) or inhibitors (KRAB/MeCP2) to the *H19* promoter region through dCas9 targeting, this method stimulated or suppressed *H19* expression in P19 cell line (Figure S5a). The unique advantage of this system is that the *H19* expression can be altered profoundly in a native chromatin structure. The P19 cell line is an established *in vitro* model for studying embryonic myogenesis, as these cells are capable of forming embryonic bodies and differentiating into embryonic myogenic cells [3]. The efficacy of overexpression and inhibition was verified by qPCR (Figure 4c). *H19* overexpression hindered myofiber formation (Figure 4d,e) and suppressed the expression of myogenic genes, except for *Myf5*, the earliest myogenic regulator factor (Figure 4f), suggesting that *H19* might not affect the early myogenic commitment in P19 cells. Unexpectedly, inhibiting *H19* also suppressed myofiber formation, and the inhibition was considerably stronger than that observed with *H19* overexpression (Figure 4d–f).

**FIGURE 5:**
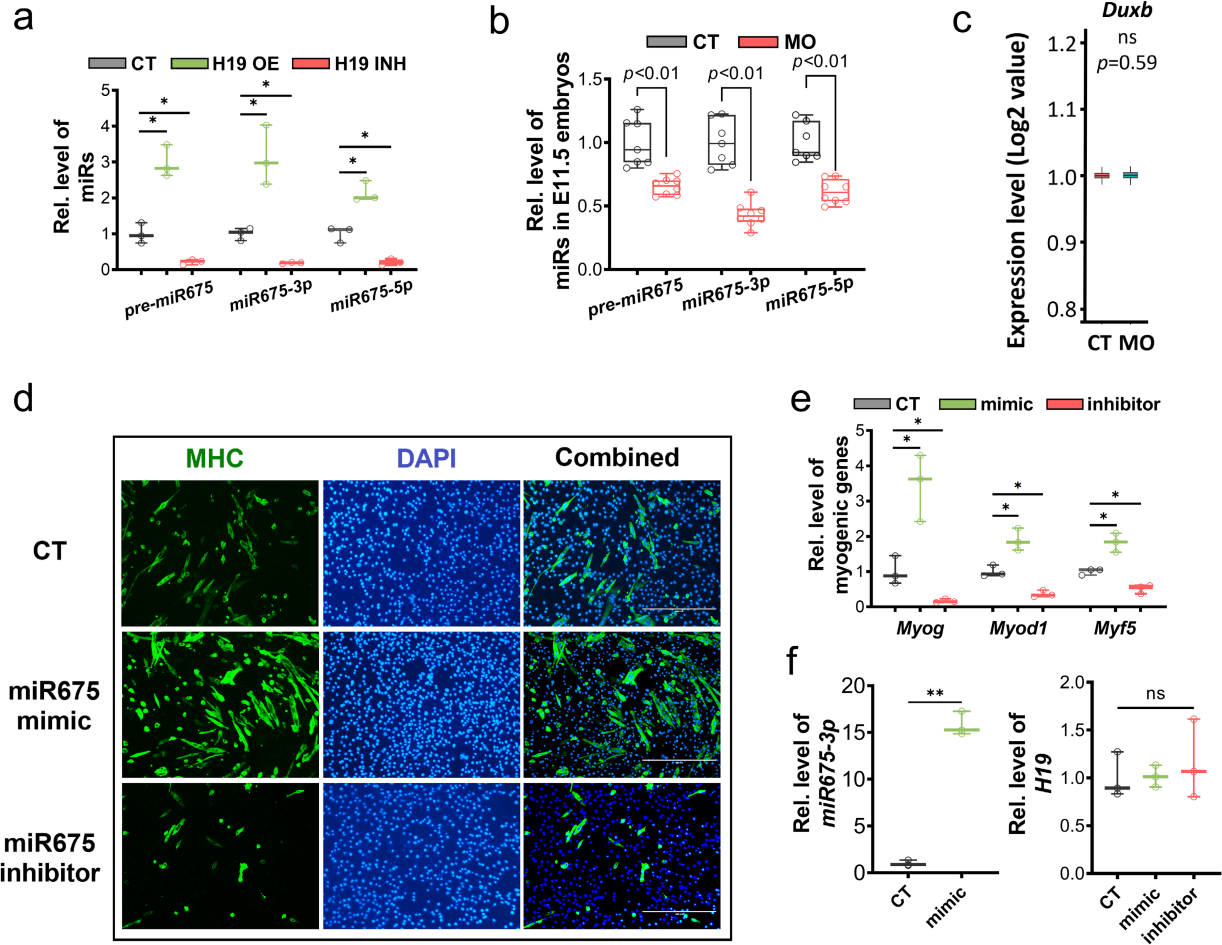
The biological effects of *miR675* on myogenesis. (a) Expression of *pre‐miR675*, *miR675‐3p* and *miR675‐5p* in P19 cells from CT, *H19* OE and *H19* INH groups (*n* = 3). (b) Expression of *pre‐miR675*, *miR675‐3p* and *miR675‐5p* in E11.5 embryos from CT and MO groups (*n* = 8). (c) Expression of *Duxb* in scRNA‐seq data from myogenic cells (E11.5 + E13.5). (d) Representative images of ICC for MHC staining in P19 cells (5‐day differentiation) from CT, *miR675* mimic and *miR675* inhibitor groups. (e) Myogenic gene expression in P19 cells from CT, *miR675* mimic and *miR675* inhibitor groups (*n* = 3). (f) Expression of *miR675‐3p* and *H19* in P19 cells from CT, *miR675* mimic and *miR675* inhibitor groups (*n* = 3). Data are presented as mean ± SD, each dot represents one cell culture dish or one pregnancy; *p* values from two‐tailed unpaired Student's *t* test; asterisk (*) indicated *p* < 0.05. CT, control; DAPI, 4,6‐diamidino‐2‐phenylindole; MHC, myosin heavy chain; miR, microRNA; ns, not significant.

Given that the first exon of *H19* harbours a myogenic *miR675* (Figure S5b), we further analysed the expression levels of *pre‐miR675*, *miR675‐3p* and *miR675‐5p* in the P19 cells with *H19* overexpression and inhibition. Inhibition of *H19* decreased the levels of *pre‐miR675*, *miR675‐3p* and *miR675‐5p*, while H19 overexpression increased their levels (Figure 5a). The expression levels of *pre‐miR675*, *miR675‐3p* and *miR675‐5p* were further confirmed in E11.5 embryos. Overall, the *miR675* bioconversion was significantly suppressed in MO E11.5 embryos (Figure 5b). Considering Dux4 is one of the targets of *miR675* in regulation of myogenesis, we analysed its expression in our scRNA‐seq data and found that *Dux4* expression was not detectable in myogenic cells (data not shown), while *Duxb* (a member of Dux4 family) expression was very low in myogenic cells and did not differ between CT and MO groups (Figure 5c); no other Dux4 members were detected. These data suggest the miR675‐Dux4 axis is unlikely to be involved in the regulation of myogenesis in E11.5 embryos.

To verify the roles of *miR675* in mediating myogenesis *in vitro*, we transfected the P19 cells with either a *miR675* mimic or inhibitor and analysed their effects on muscle differentiation. The higher level of *miR675* significantly promoted myogenesis, while *miR675* inhibition abolished myogenesis (Figure 5d,e). Successful transfection was confirmed by comparing *miR675* expression with the negative control. After transfection with *miR675* mimic, the level of *miR675* increased approximately 15‐fold, without affecting *H19* expression (Figure 5f). These data provided an explanation for the myogenic suppression due to *H19* inhibition (Figure 4d–f).

These observations indicated an antagonistic function of the *H19* lncRNA and its derived *miR675* on myogenesis, which provides an explanation for the previous inconsistent reports on *H19* and myogenesis [S1, 18]. MO inhibited the biogenesis of *miR675* from *H19*. These data underscore the critical role of *H19* to *miR675* conversion in the regulation of myogenesis.

### 3.2 MO Disrupts Imprinting of the *H19*/*Igf2* Locus

**FIGURE 6:**
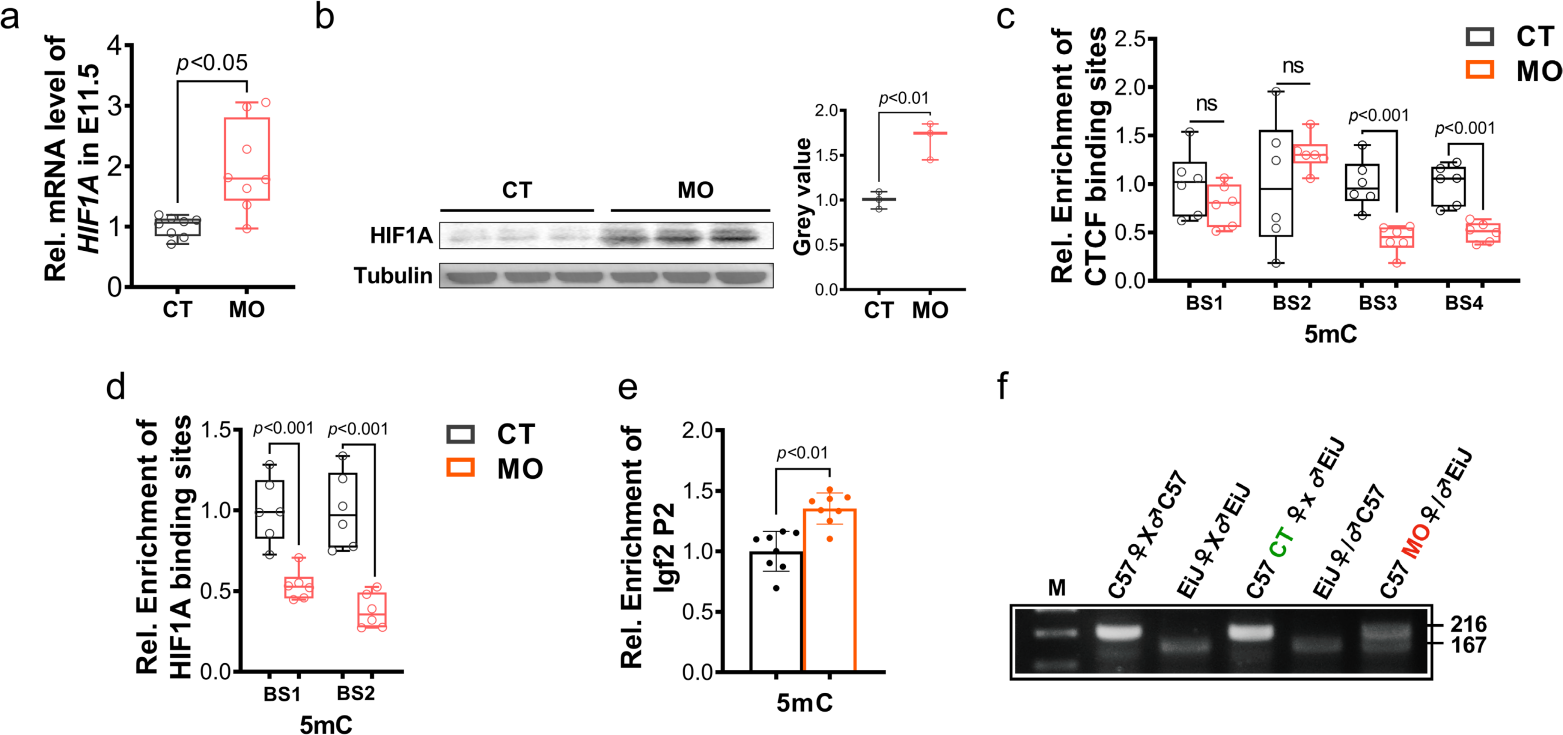
Maternal obesity induces hypoxia and disrupts imprinting of the *H19*/*Igf2* locus in E11.5 embryos. (a) Relative expression level of *Hif1a* in E11.5 embryos from CT and MO groups (*n* = 8). (b) Representative images of western blots of HIF1A protein level in E11.5 embryos from CT and MO groups (*n* = 8). (c) Methylation level of CTCF binding sites in E11.5 embryos from CT and MO groups (*n* = 6). (d) Methylation level of HIF1A binding sites in E11.5 embryos from CT and MO groups (*n* = 6). (e) Methylation level of *Igf2* Promoter 2 (P2) in E11.5 embryos from CT and MO groups (*n* = 8). (f) Allele‐specific expression analysis of *H19* in E11.5 embryos from corresponding breeding strategies. Data are presented as mean ± SD. *p* values from two‐tailed unpaired Student's *t* test. C57, C57BL/6 mice; CT, control; EiJ, CAST/EiJ mice; M, marker; MO, maternal obesity.

As mentioned above, the *H19* lncRNA was highly expressed in MO embryos, suggesting a possible disruption of *H19*/*Igf2* imprinting. To understand the causes of increased *H19* expression in MO embryos, the upstream region of the *H19* gene was analysed (Figure S5c). In addition to the previously identified CTCF binding site within the ICR region, four putative HIF1A binding sites were also found at positions −708, −647, −142 and −139 of the *H19* promoter (Figure S5d). Indeed, MO induced hypoxia in E11.5 embryos (Figure 6a,b). The scRNA data and our previous study also showed an increased hypoxia response (Figure S3a) and *Hif1a* expression [S2]. The methylation levels of ICR and HIF1A binding regions were lower in MO embryos (Figure 6c,d), suggesting increased accessibility of CTCF and HIF1A proteins to these regulatory regions. In addition, the upstream promoter region of *Igf2* was also analysed considering *Igf2* is a reciprocally imprinted gene with *H19* and also plays critical roles in embryonic development [7]. A pair of primers was designed to quantify the methylation level of *Igf2* Promoter 2, showing an increased methylation level in MO embryos (Figure 6e).

To identify the allelic expression of *H19*, we used CAST/EiJ and C57BL/6J crossed mice, which harbour SNPs in the *H19* transcripts, to identify the parental origin of *H19* expression. A pair of primers was designed to generate an amplicon where an AlwNI restriction site is present in the CAST/EiJ allele but not the C57BL/6J allele (Figure S6). Regardless of mouse breeds, only a single amplicon was detected in the CT embryos, showing an exclusive expression from the maternal allele. However, the MO C57BL/6J group exhibited double bands, indicating diallelic expression of *H19* (Figure 6f).

Furthermore, we utilized low oxygen–treated P19 cells to simulate the MO‐induced hypoxia in embryos. Exposure of P19 cells to low oxygen levels (1% oxygen) for 24‐h induced cell hypoxia, as indicated by increased mRNA and protein levels of HIF1A (Figure S7a). Hypoxia dramatically inhibited myofiber formation (Figure S7b) and reduced expression of myogenic genes (Figure S7c), as well as the MHC content (Figure S7d). Consistent with previous data, hypoxia up‐regulated *H19* expression (Figure S7e). We also analysed the expression of HuR, another stress sensor protein that is involved in regulation of HIF1A and *miR675* [19, 20]. No significant changes were observed (Figure S7f).

### 3.3 MO Suppresses Phosphorylation of KHSRP to Block miR675 Maturation

**FIGURE 7:**
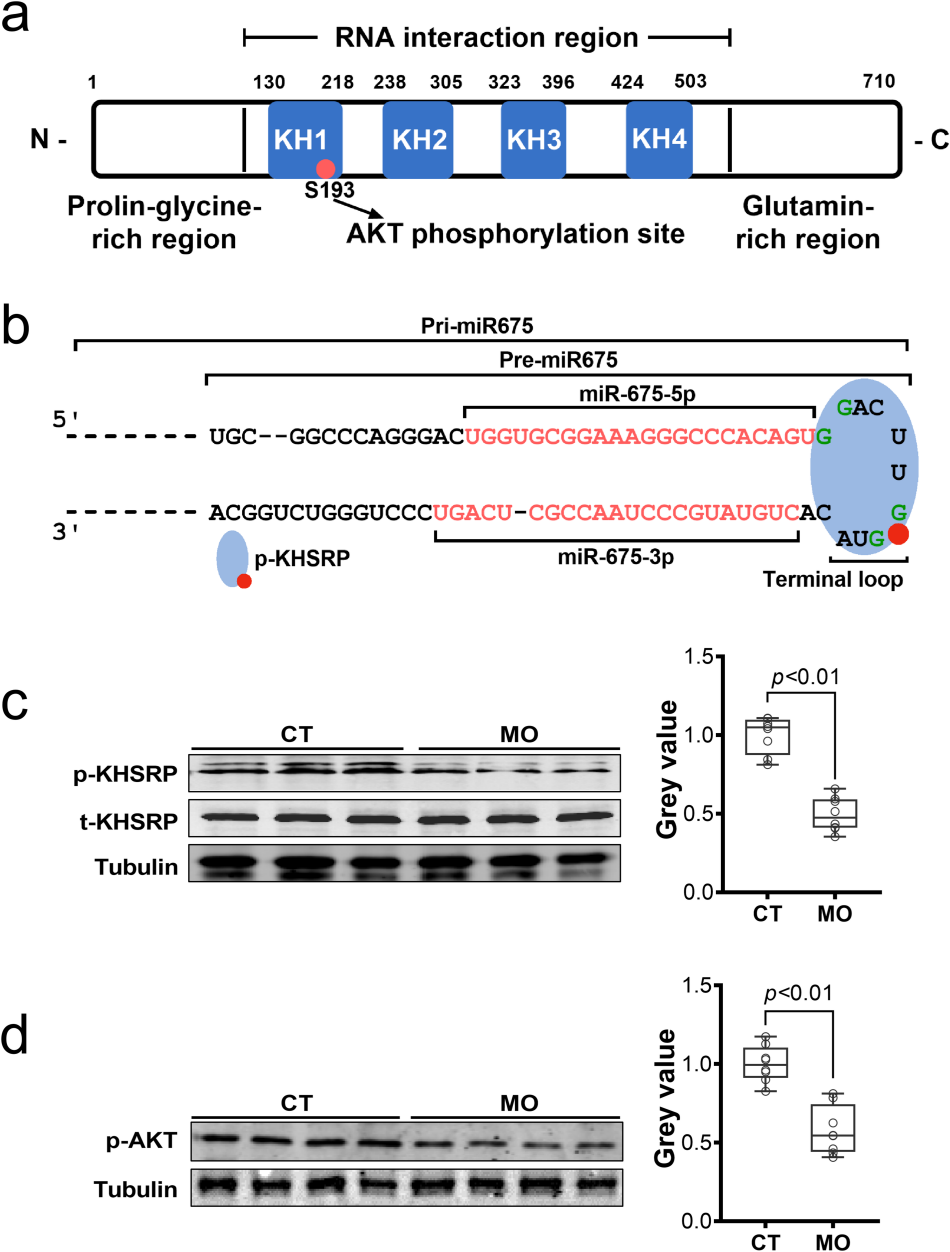
Maternal obesity inhibits AKT and KHSRP phosphorylation in E11.5 embryos. (a) Schematic diagram of KHSRP protein structure. Red dot indicates AKT phosphorylation site. (b) Schematic diagram of *pri‐miR675*, *pre‐miR675*, *miR675‐3p*, *miR675‐5p* sequence, terminal loop structure. (c) Representative images of western blots and quantification of p‐KHSRP and t‐KHSRP protein levels in E11.5 embryos from CT and MO groups (*n* = 8). (d) Representative images of western blots and quantification of p‐AKT protein levels in E11.5 embryos from CT and MO groups (*n* = 8). Data are presented as mean ± SD. *p* values from two‐tailed unpaired Student's *t* test. CT, control; KH, K homology (KH) domain; MO, maternal obesity; p, phosphorylation.

To further investigate the underlying mechanisms blocking *H19* to *miR675* bioprocessing, we focused on KHSRP, a multifunctional RNA‐binding protein involved in both lncRNA function and miRNA processing [12]. KHSRP is phosphorylated by AKT at serine‐183 (Figure 7a), and p‐KHSRP translocates from the cytoplasm into nucleus, where it associates with the GG‐rich terminal loop of pri‐miR and pre‐miR to facilitate their maturation [21]. Interestingly, we identified a GG‐rich binding site in the terminal loop of *pri‐miR675* and *pre‐miR675* (Figure 7b), suggesting that KHSRP mediates the bioconversion of *H19* to *miR675*. Therefore, we analysed the p‐KHSRP content, which was reduced in MO embryos (Figure 7c), explaining the attenuated *miR675* processing observed in MO embryos (Figure 5b).

To further confirm the role of KHSRP in *miR675* processing, we knocked down *Khsrp* using siRNA transfection in P19 cells. As expected, *Khsrp* knockdown impaired myogenesis (Figure S8a–c) and decreased *miR675* processing (Figure S8d). In aggregate, these data demonstrated that *miR675* biogenesis from *H19* promotes myogenesis in a KHSRP‐dependent manner.

During the prenatal development, IGF2 is the major growth factor activating AKT [22]. We found that the E11.5 embryos from MO group had significantly lower mass (Figure S9a), decreased *Igf2* (Figure S9b) and *Igf1* expression, but increased *Igf2r* and *Igf1r* (Figure S9c), likely due to the higher *H19* content [23]. IGF2R is a repressive receptor of IGF2 that can clear the IGF2 and attenuate its signalling [24]. Consistent with the decreased *Igf2* and elevated Igf2r expression, the phosphorylation level of AKT was decreased in MO embryos (Figure 7d), in agreement with a decrease in p‐KHSRP level (Figure 7c). Additionally, lower β‐catenin protein level was observed in MO embryos (Figure S9d), indicating an inhibited Wnt/β‐catenin signalling, which is consistent with previous studies [S9].

**FIGURE 8:**
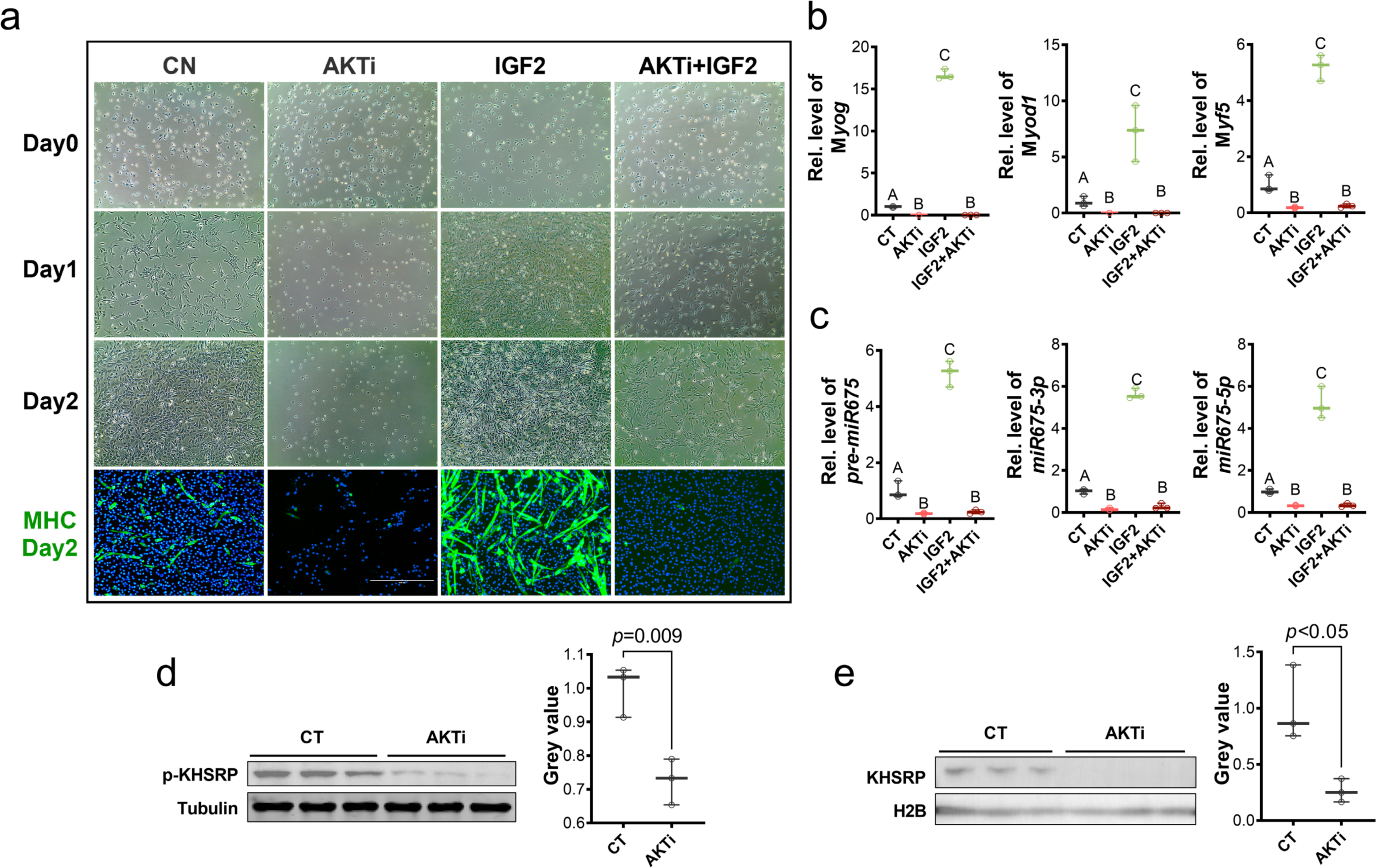
The effects of AKT activation and inhibition on KHSRP‐regulated myogenesis. (a) Representative images of ICC for MHC staining in P19 cells (5‐day differentiation) from CT, AKTi, IGF2 and AKTi + IGF2 groups. (b,c) Relative expression level of corresponding transcripts in P19 cells from CT, AKTi, IGF2 and AKTi + IGF2 groups (*n* = 3). (d) Representative images of western blots of p‐KHSRP protein levels in P19 cells from CT and AKTi groups (*n* = 3). (e) Representative images of western blots of KHSRP protein level in the nuclei of P19 cells from CT and AKTi groups (*n* = 3). Data are presented as mean ± SD. *p* values from two‐tailed unpaired Student's *t* test. AKT was inhibited or activated by AKT inhibitor PHT427 or activator IGF2, respectively. AKTi, AKT inhibitor; CT, control.

To further investigate the role of AKT signalling in myogenesis and *miR675* biogenesis, we treated P19 cells with an AKT inhibitor, which inhibited myogenesis (Figure 8a,b) and decreased *miR675* maturation (Figure 8c). Correspondingly, the phosphorylation level of KHSRP was decreased (Figure 8d), and its nuclear translocation was inhibited (Figure 8e). Conversely, treatment with IGF2 elevated the phosphorylation of AKT and KHSRP, which promoted myogenesis (Figure 8a,b) and *miR675* biogenesis (Figure 8c). These effects of IGF2 were blocked by AKT inhibitor (Figure 8a–c), showing that AKT‐induced KHSRP phosphorylation promotes myogenesis by facilitating biogenesis of *miR675*. In conclusion, MO inhibits IGF2 and AKT signaling, which suppresses KHSRP‐mediated *miR675* biogenesis and myogenesis.

Overall, our study demonstrated that MO disrupts the imprinting of *H19*/*Igf2* locus, resulting in the diallelic expression of *H19* lncRNA and decreased *Igf2* expression. This disruption inhibits embryonic myogenesis. The decreased level of *Igf2* further reduced AKT signalling and p‐KHSRP level. Consequently, KHSRP promotes myogenic mRNA decay while its function to promote miRNA biogenesis is blocked, deteriorating embryonic myogenesis due to MO (Figure S10). This novel mechanism shows the antagonistic function of *H19* lncRNA and its embedded *miR675* in myogenesis, and MO disrupts *H19*/*Igf2* imprinting to suppress KHSRP‐mediated *miR675* biogenesis.

## 4 Discussion

MO is occurring in more than 40% of pregnancies in the United States now [1]. Skeletal muscle comprises 30%–40% of body mass and is the main tissue utilizing glucose and fatty acids. Compromised muscle function leads to insulin resistance and precedes the development of Type II diabetes [S10]. MO impairs fetal skeletal muscle development, impairing skeletal muscle contractile force and metabolic functions in offspring [3]. Recently, we further discovered that myogenic suppression occurs as early as the embryonic stage [25]. However, mechanisms remain poorly characterized.

Imprinted genes are evolutionally selected for their critical roles in regulating fetal and placental development, with *H19*/*Igf2* to be the first identified imprinting cluster [26]. Imprinting disorders of the *H19*/*Igf2* cluster are quite common, and loss of *H19* and *Igf2* imprinting is present in 4% and 22% human neonates [27], respectively. The *H19*/*Igf2* locus accounts for 31% newborn weight variance [10], despite largely undefined biological significance. In this study, we found that MO induces *H19* expression from the silenced paternal allele, showing disruption of *H19*/*Igf2* imprinting.

To explore the mechanisms elevating *H19* expression in MO embryos, we analysed the *H19* promoter and found conserved HIF1a binding sites, suggesting that hypoxia induced by MO might contribute to the disruption of imprinting at the *H19*/*Igf2* locus. Hypoxia, via the transcription factor HIF1A, alters DNA methylation levels surrounding its binding sites in the *H19* promoter leading to the imprinting disruption. However, another stress sensor protein, HuR that is involved in the activation of HIF1A and processing of lncRNA *H19* [20], was not altered due to MO, suggesting HuR was not involved. These findings provide molecular insights into the effects of MO in affecting the epigenetic landscape of progenitor cells undergoing myogenesis. Other essential epigenetic mechanisms such as histone modifications and *ncRNA* interactions might also be affected by MO during embryonic development [28]. A recent study shows that MO‐induced metabolism disorder restricts the metabolite supply into the embryo, which is necessary for cells to acquire methylation [29]. Moreover, there are several enhancers and motifs in the *H19* gene locus [30], suggesting the intricate regulation of *H19* expression.

To further analyse the biological effects of *H19* imprinting disruption, we capitalized on CRISPR/dCas9 to alter expression of *H19* in a native chromatin structure. This method is necessary because *H19* is transcribed and processed mainly inside nuclei. We reported that both overexpression and inhibition of *H19* in P19 cells hindered myofiber formation and suppressed the expression of myogenic genes, highlighting its complex role in myogenesis. Our subsequent studies demonstrated the antagonistic functions of *H19* and *miR675* in myogenesis. Their ratio may influence the myogenesis in a concentration‐sensitive manner [31]. In previous studies, there are conflicting reports on the roles of H19 and its embedded *miR675* in myogenesis [32]. *H19* was reported to have both promyogenic and antimyogenic functions [33], with unclear mechanisms [11]. This is the first time that *H19* and *miR675* were comprehensively analysed for their roles in myogenesis. These findings underscore the importance of *miR675* biogenesis in regulating embryonic myogenesis, which was inhibited in MO embryos.

KHSRP was identified as a key player in the processing of miRNAs with a GG‐enriched stem loop [14]. We identified GG‐rich stem loop structure in *H19*, showing the potential mediatory roles of KHSRP in the bioconversion of *H19* into *miR675*. Function of KHSRP depends on its phosphorylation by AKT, a key downstream mediator of IGF2 signalling [12]. We found that *Igf2* expression was lower in MO embryos, in agreement with the decreased phosphorylation of KHSRP, as well as inhibited *miR675* biogenesis and suppressed myogenesis in MO embryos. Because *H19* and *Igf2* expression are inversely regulated [7], these data show an intriguing relationship between *H19*/*Igf2* imprinting disruption and myogenic suppression due to MO, which is intricately regulated by the IGF2/AKT‐mediated KHSRP phosphorylation and *miR675* biogenesis. Furthermore, we observed an increase of *Igf2r* in MO embryonic cells, an inhibitory receptor that attenuates IGF2 signalling [34]. This further strengthens our findings that MO inhibits IGF2/AKT signalling.

LncRNA *H19* has been reported to promote the expression of *Igf1r* via sponging miR *Let7* [35], consistent with a slight increase of *Igf1r* and decrease of *Igf1* in myogenic cells from MO embryos. Considering the biogenesis of miR *Let7* is also regulated by KHSRP [36], further studies are needed to comprehensively analyse the possible involvement of this pathway. Additionally, a recent study showed that in human, *miR675* rescued myogenesis through targeting DUX4 protein, a transcription factor inducing muscle dystrophy [37], but our data indicate that Dux4 is not expressed in embryonic myogenic cells. Moreover, we observed a decreased expression level of β‐catenin in E11.5 MO embryos, along with lower embryo mass. In sheep, MO has been shown to suppress fetal muscle development by inhibiting Wnt/β‐catenin [S9]. Our recently published study demonstrated that along the limb axis of E13.5 mouse embryos, Wnt/β‐catenin signalling was down‐regulated due to MO according to GO analysis of spatial sequencing data [25]. Mechanistically, lncRNA H19 sponges miR‐29a, by which promotes the expression of β‐catenin [38]. Interestingly, KHSRP itself is capable of stabilizing the transcript level of β‐catenin once it is phosphorylated by AKT [39]. All these findings sum up to the complexity of regulatory roles of KHSRP/*H19*/*miR675* axis in embryonic myogenesis.

In conclusion, this study sheds light on the molecular mechanisms underlying the impact of MO on embryonic myogenesis. The findings have significant implications for understanding developmental disorders associated with MO and suggest that the regulation of embryonic myogenesis relies on the precise spatial–temporal expression of *H19* and its conversion into *miR675*. We propose a mechanistic model that integrates the roles of *H19* lncRNA to *miR675* bioconversion and IGF2‐mediated AKT signalling and *H19*/*IGF2* imprinting disruption in the regulation of embryonic myogenesis affected by MO.

## Conflicts of Interest

The authors declare no conflicts of interest.

## Supporting information


**Data S1.** Supporting Information.

**Data S2.** Supporting Information.

**Data S3.** Supporting Information.

**Data S4.** Supporting Information.

**Data S5.** Supporting Information.

**Data S6.** Supporting Information.

**Data S7.** Supporting Information.
