# Supplementary material for: Balancing LncRNA H19 and miR‐675 Bioconversion as a Key Regulator of Embryonic Myogenesis Under Maternal Obesity
Source: J Cachexia Sarcopenia Muscle. 2025 Mar 31;16(2):e13791. doi: 10.1002/jcsm.13791 (PMC11955836; doi:10.1002/jcsm.13791)
Supplement: Supplementary file 1 — Data S1. Supporting Information. [file JCSM-16-e13791-s007.docx]

**Supplementary Table 1. Primers used in this study**

|  | **Primers** | **Gene RefSeq** |  | **Forward/Reverse** | **Amplicon length (bp)** | **Primer sequence** |
| --- | --- | --- | --- | --- | --- | --- |
|  | ***H19*** | NR_130973.1 |  | **F** | 177 | GAGACTCAAAGCACCCGTGA |
|  |  |  |  | **R** |  | AGATGGACGACAGGTGGGTA |
|  | ***Igf2*** | NM_001122736.2 |  | **F** | 318 | TGCCTCAACTCAGTCCCTCT |
|  |  |  |  | **R** |  | ACACAGCACCCATCTTGAGAC |
|  | ***Myf5*** | NM_008656.5 |  | **F** | 157 | CGGATCACGTCTACAGAGCC |
|  |  |  |  | **R** |  | GCAGGAGTGATCATCGGGAG |
|  | ***Myod1*** | NM_010866.2 |  | **F** | 150 | CGACACCGCCTACTACAGTG |
|  |  |  |  | **R** |  | GGTGGTGCATCTGCCAAAAG |
|  | ***Myog*** | NM_031189.2 |  | **F** | 82 | TCCCAACCCAGGAGATCATT |
|  |  |  |  | **R** |  | GCTGTCCACGATGGACGTAA |
| ***qPCR*** | ***Pri-miR675*** | NR_130973.1 |  | **F** | 163 | ACACTGTATGCCCTAACCGC |
|  |  | MI0004123 |  | **R** |  | CAGCTACTCGCTCTACCTGC |
|  | ***Pre-miR675*** | MI0004123 |  |  |  | ACAGTGGACTTGGTACACT |
|  |  |  |  |  |  |  |
|  | ***miR675-3p*** | MIMAT0003726 |  |  |  | CTGTATGCCCTAACCGCTCAGT |
|  |  |  |  |  |  |  |
|  | ***miR675-5p*** | MIMAT0003725 |  |  |  | TGGTGCGGAAAGGGCCCACAGT |
|  |  |  |  |  |  |  |
|  |  |  |  |  |  |  |
|  | **Genes** | **RefSeq** | **Chromosome location** | **Forward/Reverse** | **Amplicon length (bp)** | **Primer sequence** |
|  | ***CTCF*-** | NC_000073.7 | 142135839-142135819 | **F** | 209 | TGCCCTATTCTTGGACGTCTG |
|  | ***bs1*** |  | 142135631-142135651 | **R** |  | GAAAATGCATGTGTCCTGCCC |
| ***Chip-qPCR*** | ***CTCF*-** |  | 142135557-142135538 | **F** | 129 | TGTAAAGACCAGGGTTGCCG |
|  | ***bs2*** |  | 142135429-142135449 | **R** |  | GCCTCATGAAGCCCATGACTA |
|  | ***CTCF*-** |  | 142134539-142134520 | **F** | 250 | ATGTGCAACAAGGGAACGGA |
|  | ***bs3*** |  | 142134290-142134309 | **R** |  | TCGGCAACTTCGGTCTTACC |
|  | ***CTCF*- bs4** |  | 142134058-142134039  142133898-142133917 | **F**  **R** | 161 | CGCGTGGTGGCAGTACAATA  TGGCTAGCTTGAGGAGTCCC |
|  | ***HIF1A-*** |  | 142132444-142132425 | **F** | 145 | TCCTGACAAACGTGACGACT |
|  | ***bs1*** |  | 142132300-142132320 | **R** |  | AACAGCCTACGAGTGGCTAAC |
|  | ***HIF1A-*** |  | 142132064-142132045 | **F** | 107 | AGAATTTCAGGACGGGTGCG |
|  | ***bs2*** |  | 142131958-142131977 | **R** |  | CCAATCAGTACATGGCCCCG |
|  | ***Igf2-P2*** | MGI:96434 | 142211239 - 142211395 | **F** | 156 | TGCTATTCGAGGTGCCTCAC |
|  |  |  |  | **R** |  | TGAGTTAAGGCGCAGGTAGC |
|  |  |  |  |  |  |  |
| ***dCas9 gRNA-H19*** |  | CAATCAGTACATGGCCCCGCCGG  AACGTGCGCTGGAACGATACAGG | | | | |
| ***Amplicon contains***  ***AlwNI cutting site*** | B6 allele 216 | GCTATACCTTCACTGCCCAGG  GTTGAAGGACTGAGGGGCTA | | | | |
|  | CAST/EiJ allele 49+167 |  |  |  |  |  |
