# Supplementary material for: Balancing LncRNA H19 and miR‐675 Bioconversion as a Key Regulator of Embryonic Myogenesis Under Maternal Obesity
Source: J Cachexia Sarcopenia Muscle. 2025 Mar 31;16(2):e13791. doi: 10.1002/jcsm.13791 (PMC11955836; doi:10.1002/jcsm.13791)
Supplement: Supplementary file 2 — Data S2. Supporting Information. [file JCSM-16-e13791-s006.docx]

**Supplemental References**

S1. Luo W, Lin Z, Chen J, Chen G, Zhang S, Liu M, et al. TMEM182 interacts with integrin beta 1 and regulates myoblast differentiation and muscle regeneration. J Cachexia Sarcopenia Muscle. 2021;12:1704-23. doi:10.1002/jcsm.12767

S2. Zhao L, Law NC, Gomez NA, Son J, Gao Y, Liu X, et al. Obesity impairs embryonic myogenesis by enhancing BMP signaling within the dermomyotome. Advanced Science. 2021;8:2102157.

S3. Chan AOK, Dong M, Wang L, Chan WY. Somite as a morphological reference for staging and axial levels of developing structures in mouse embryos. Neuroembryology and Aging. 2005;3:102-10.

S4. Gao Y, Zhao L, Son JS, Liu X, Chen Y, Deavila JM, et al. Maternal Exercise Before and During Pregnancy Facilitates Embryonic Myogenesis by Enhancing Thyroid Hormone Signaling. Thyroid. 2022;32:581-93. doi:10.1089/thy.2021.0639

S5. Kruse AR, Malek MC, Allen J, Farrow M, Spraggins J. GeoMx-NGS Manual RNA Slide Preparation Protocol. 2023;

S6. Ma L, Meng Y, An Y, Han P, Zhang C, Yue Y, et al. Single-cell RNA-seq reveals novel interaction between muscle satellite cells and fibro-adipogenic progenitors mediated with FGF7 signalling. J Cachexia Sarcopenia Muscle. 2024;15:1388-403. doi:10.1002/jcsm.13484

S7. Hao Y, Hao S, Andersen-Nissen E, Mauck WM, Zheng S, Butler A, et al. Integrated analysis of multimodal single-cell data. Cell. 2021;184:3573-87. e29.

S8. Merritt CR, Ong GT, Church SE, Barker K, Danaher P, Geiss G, et al. Multiplex digital spatial profiling of proteins and RNA in fixed tissue. Nat Biotechnol. 2020;38:586-99. doi:10.1038/s41587-020-0472-9

S9. Tong JF, Yan X, Zhu MJ, Ford SP, Nathanielsz PW, Du M. Maternal obesity downregulates myogenesis and beta-catenin signaling in fetal skeletal muscle. Am J Physiol Endocrinol Metab. 2009;296:E917-24. doi:10.1152/ajpendo.90924.2008

S10. Lowell BB, Shulman GI. Mitochondrial dysfunction and type 2 diabetes. Science. 2005;307:384-7. doi:10.1126/science.1104343
