# Supplementary material for: Balancing LncRNA H19 and miR‐675 Bioconversion as a Key Regulator of Embryonic Myogenesis Under Maternal Obesity
Source: J Cachexia Sarcopenia Muscle. 2025 Mar 31;16(2):e13791. doi: 10.1002/jcsm.13791 (PMC11955836; doi:10.1002/jcsm.13791)
Supplement: Supplementary file 3 — Data S3. Supporting Information. [file JCSM-16-e13791-s001.pdf]

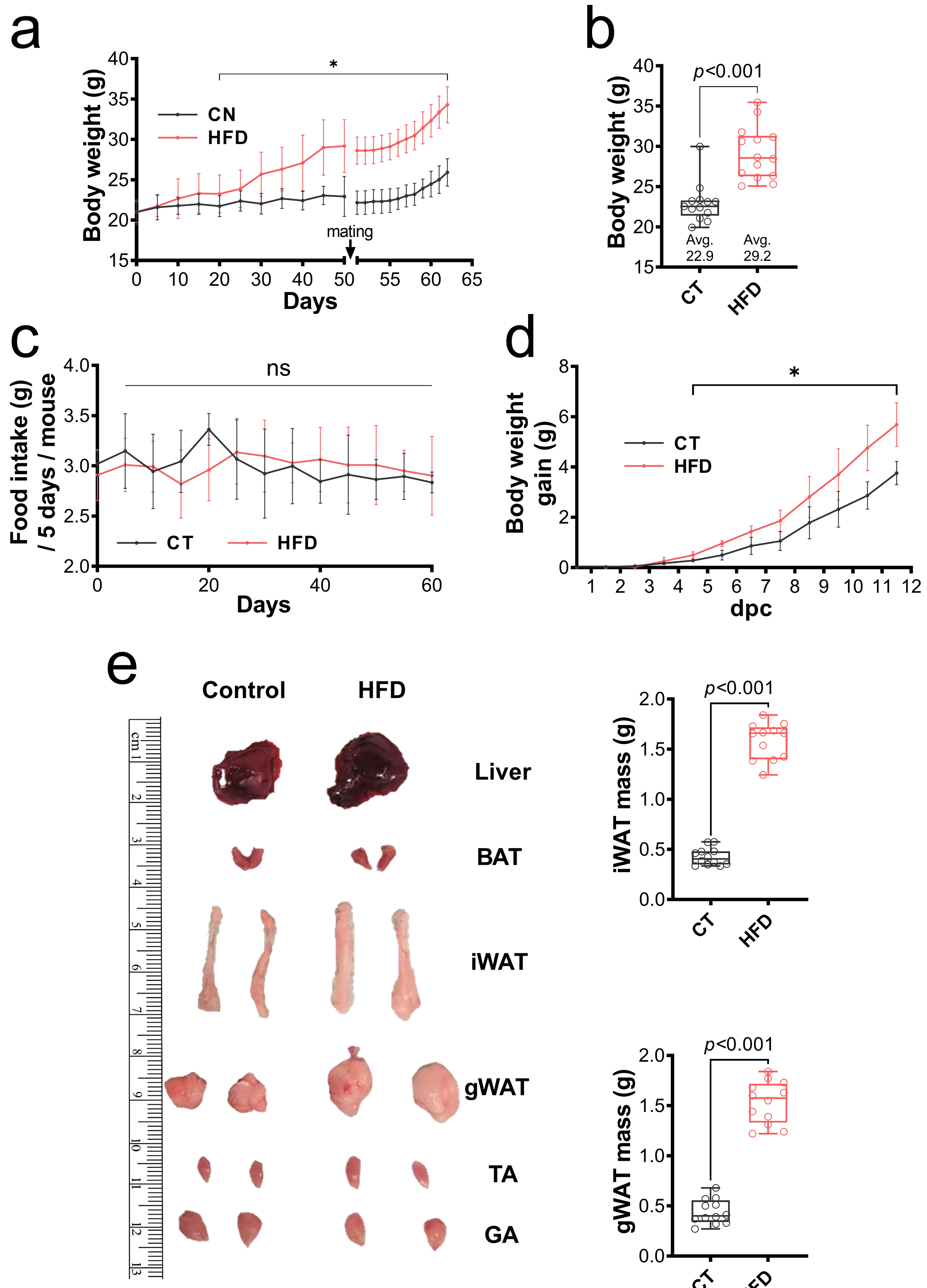

Fig. S1 | HFD feeding induces maternal obesity. a Body weight change of CT and HFD maternal mice throughout the experiment (n=14). **b** Average body weight of CN and HFD maternal mice at mating day (n=14). **c** Food intake of CT and HFD maternal mice throughout the experiment (n=5). **d** Body weight gain of CT and HFD maternal mice during pregnancy (n=14). The weight gain was calculated by comparing the weight increase since E0.5. **e** The representative picture of liver, BAT, WATs and limb skeletal muscles from CN and HFD maternal mice (n=12). Data are presented as mean ± SD, each dot represents one maternal mouse; *p* values from two-tailed unpaired Student's t test. Asterisk (*) indicated *p*<0.05 (B). CT, control; HFD, high-fat diet; BAT, brown adipose tissue; iWAT, inguinal white adipose tissue; gWAT, gonadal white adipose tissue; TA, tibialis anterior; GA, gastrocnemius; dpc, days post coitus.

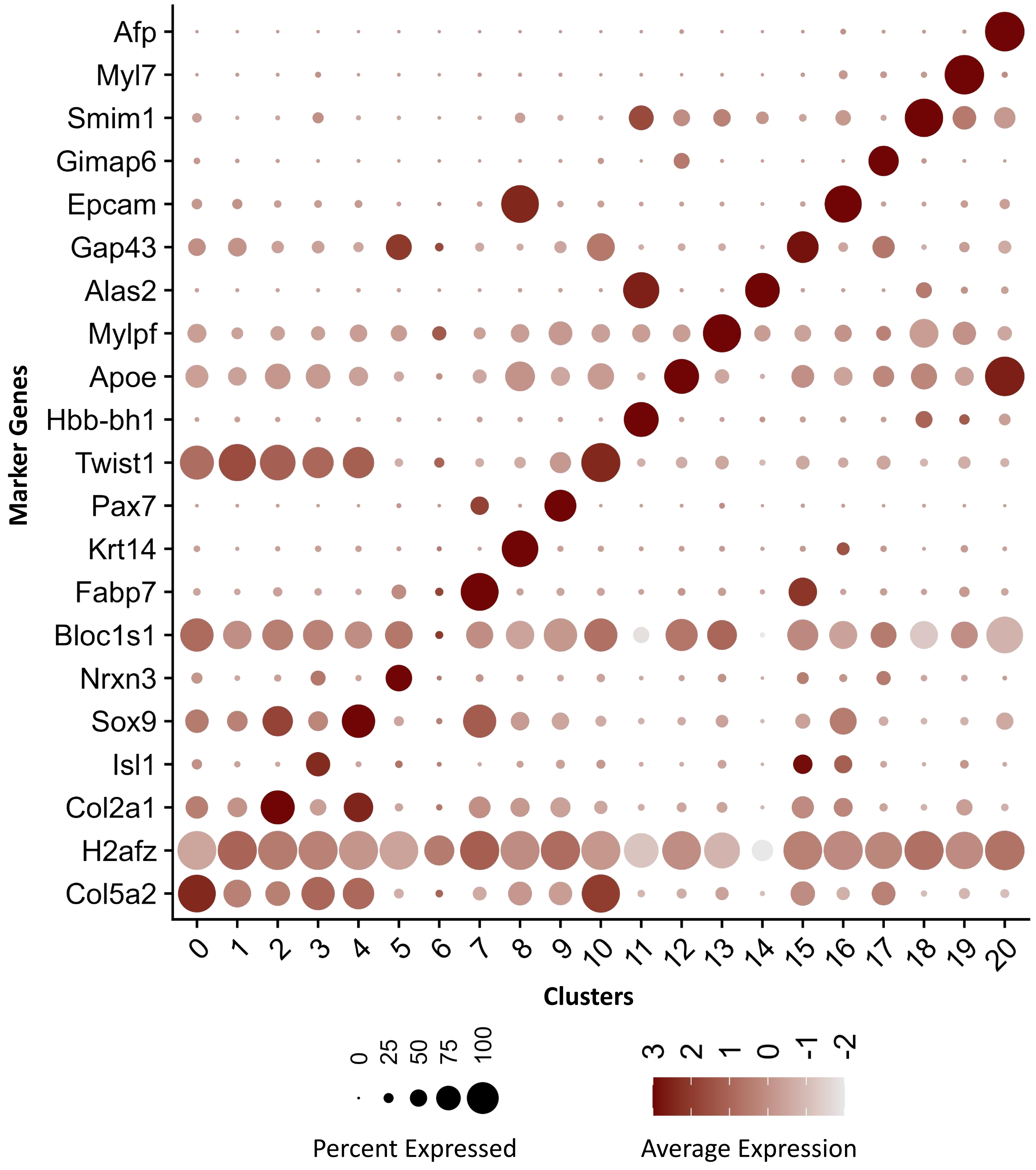

Fig. S2 | Single-cell transcriptomic profiling of embryos. Marker genes for each cell cluster.

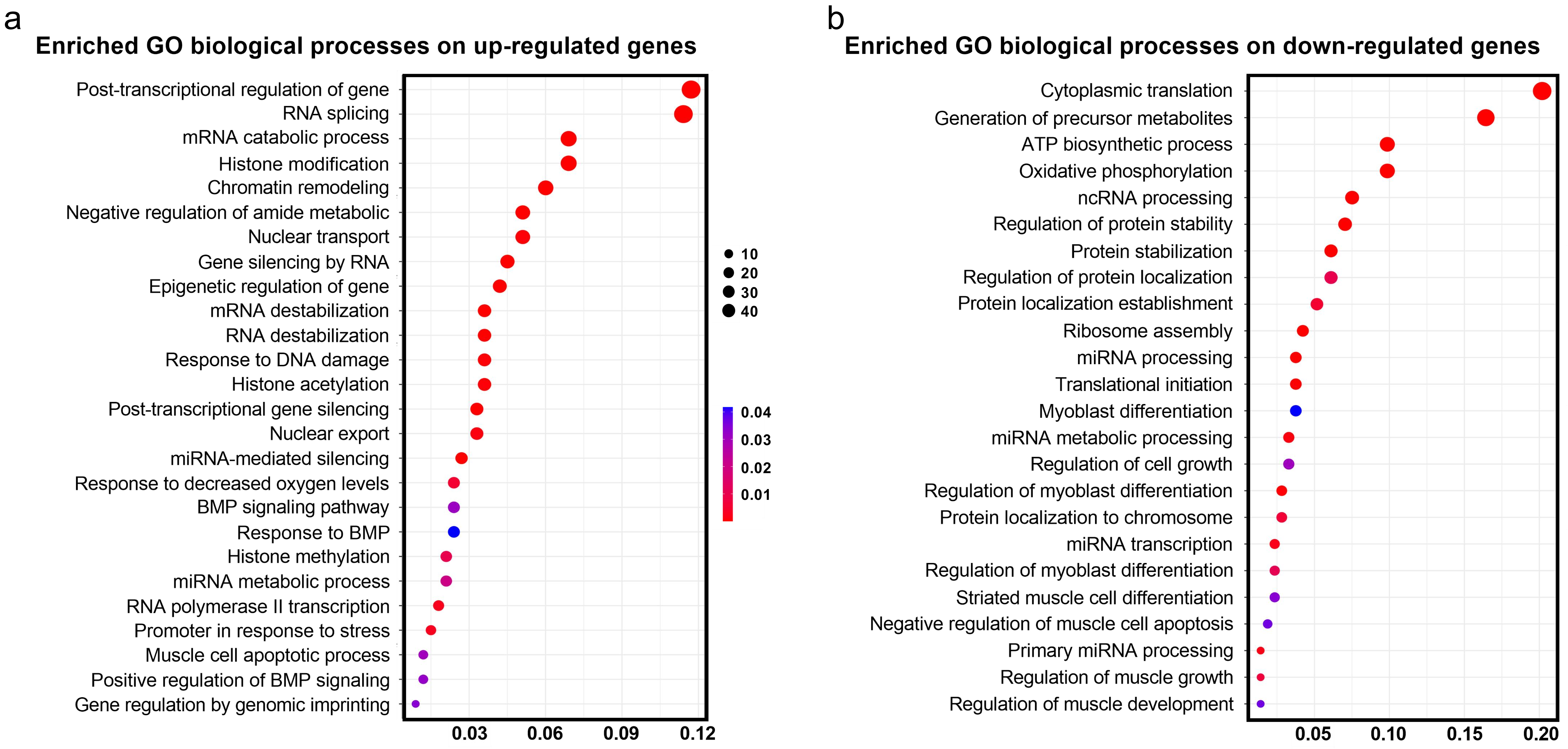

Fig. S3 | The GO enrichment analysis of up-regulated and down-regulated genes in MO myogenic cells. a Enriched GO biological processes on up-regulated genes in MO myogenic cells. **b** Enriched GO biological processes on down-regulated genes in MO myogenic cells. GO, gene ontology.

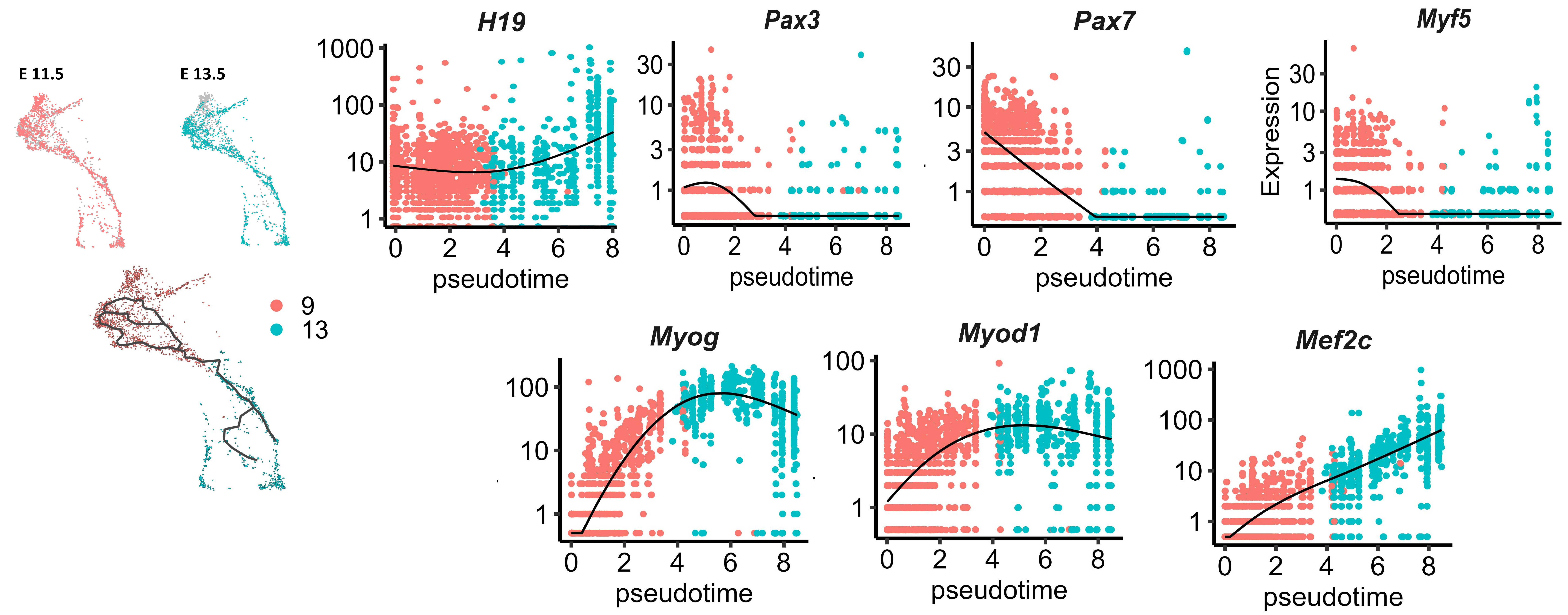

Fig. S4 | Single-cell transcriptomic profiling of embryos. Pseudo-time trajectory analysis of *H19* in myogenic cells.

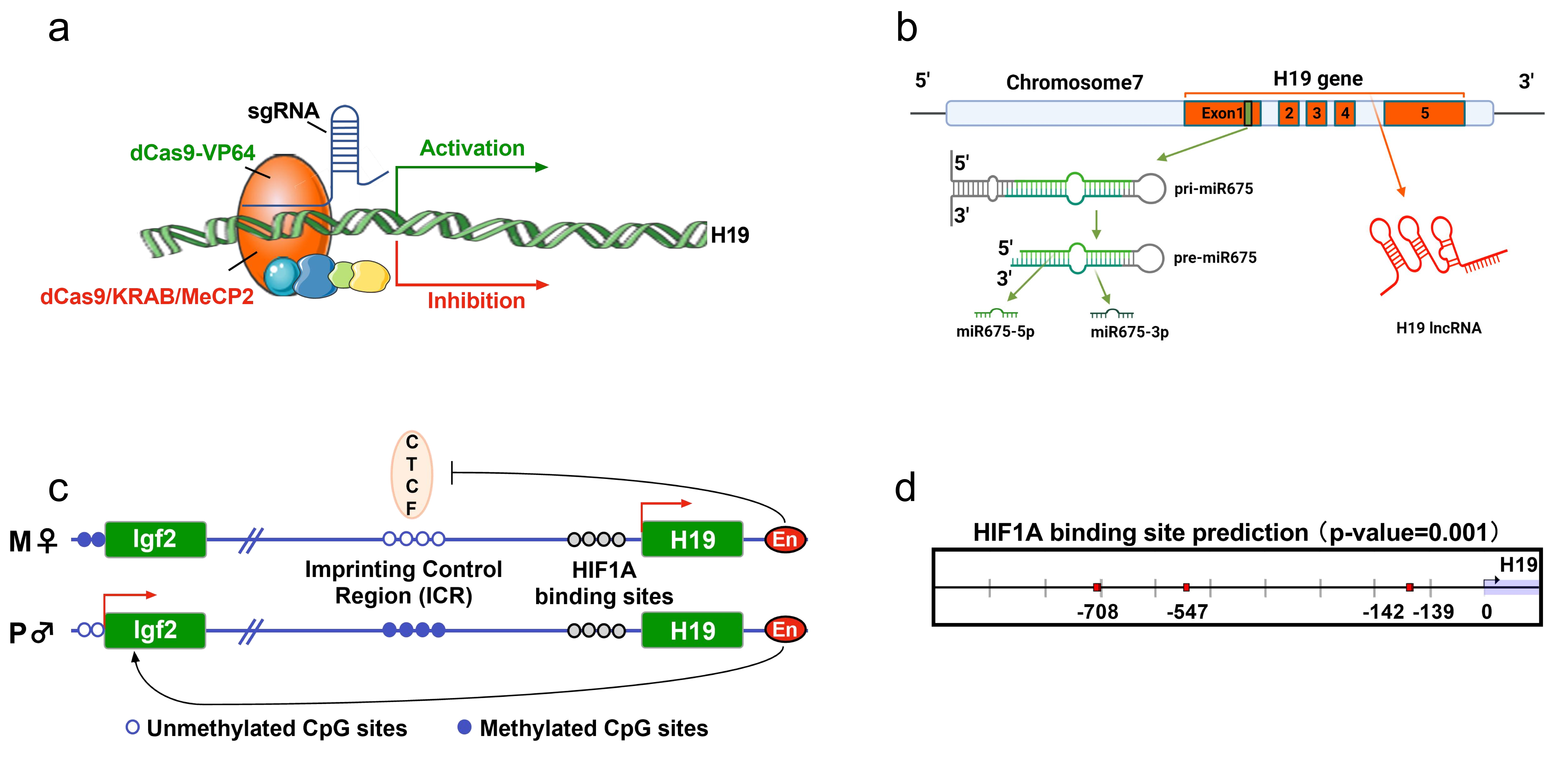

Fig. S5 | Schematics. a dCas9 activation and inhibition mechanism. **b** H19 genomic structure harboring miR675 and H19 lncRNA. **c** Schematic diagram of H19/Igf2 locus. The blue shaded circle indicates methylated CTCF binding sites. The blank circle indicates unmethylated CTCF binding site. The grey shaded circle indicates HIF1A binding site. The red oval indicates enhancer. **d** Putative HIF1A binding site in H19 promoter region.

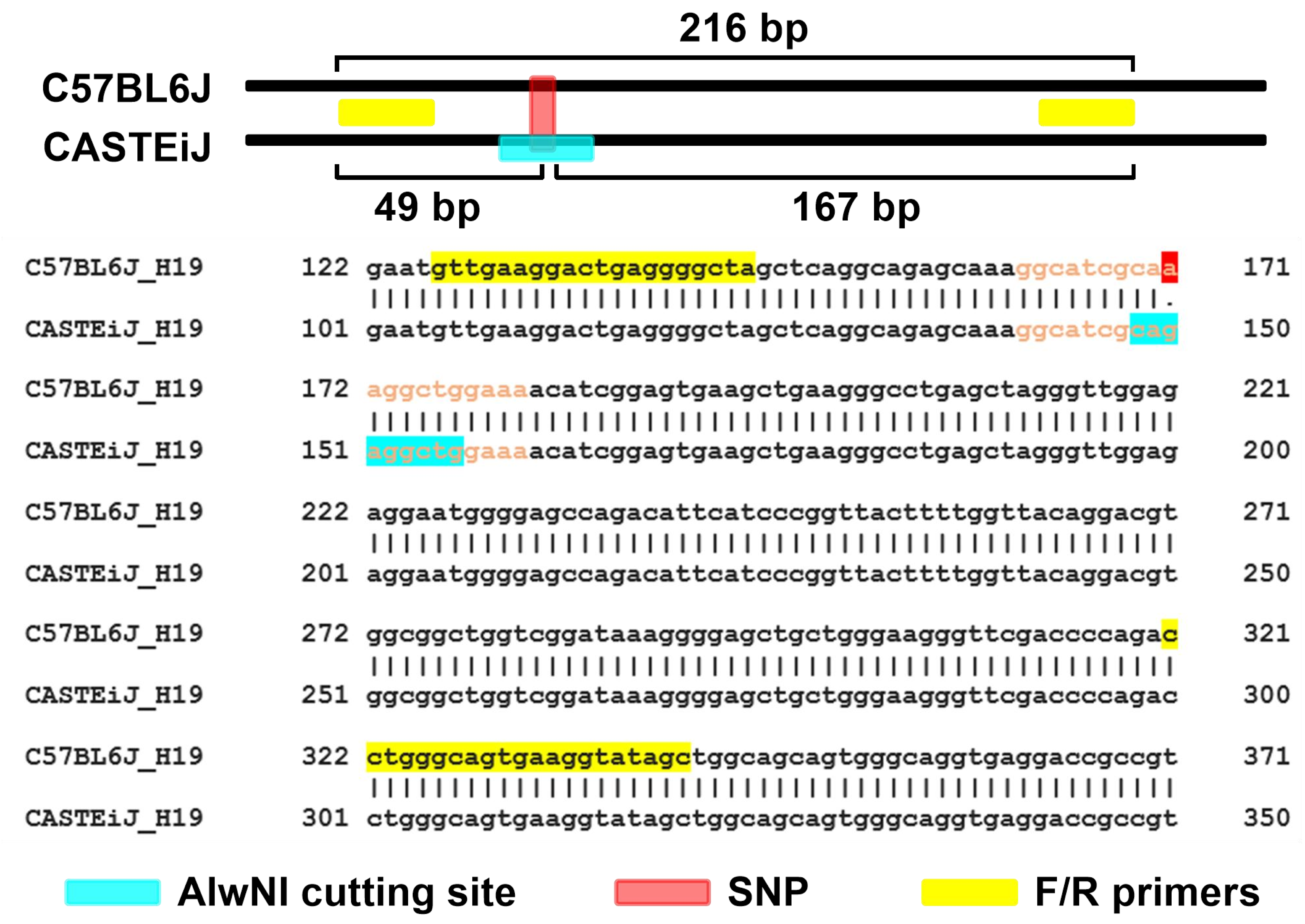

Fig. S6 | Schematic diagram of SNP and restriction enzyme cutting site design in *H19* gene from C57 and EiJ mice. SNP, single-nucleotide polymorphism; F/R primers, forward/reverse primers.

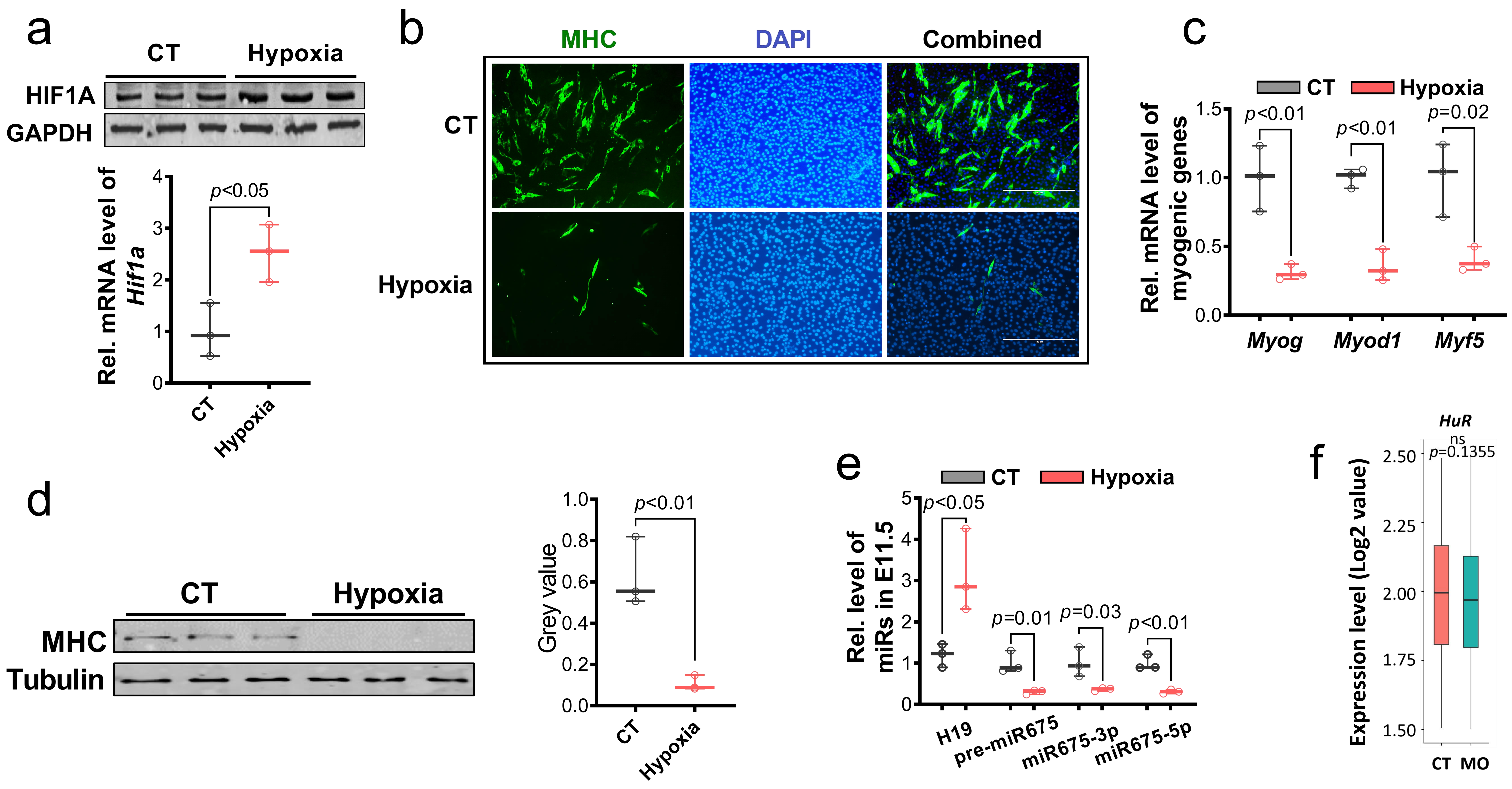

Fig. S7 | Hypoxia inhibits myogenesis in P19 cells. a Expression levels of *Hif1a* mRNA and HIF1A protein in P19 cells from CT and hypoxia groups (n=3). **b** Representative images of ICC for MHC staining in P19 cells (5-day differentiation) from CT and hypoxia groups. **c** Myogenic gene expression in P19 cells from CT and hypoxia groups (n=3). **d** Western blots of MHC protein level in P19 cells from CT and hypoxia groups (n=3). **e** Expression levels of *H19*, pre-*miR675*, *miR675-3p,* and *miR675-5p* in P19 cells from CT and hypoxia groups (n=3). **f** Expression level of *HuR* in scRNA-seq data from myogenic cells. The Data are presented as mean ± SD; *p* values from two-tailed unpaired Student's t test. CT, control; MHC, myosin heavy chain; DAPI, 4,6-diamidino-2-phenylindole.

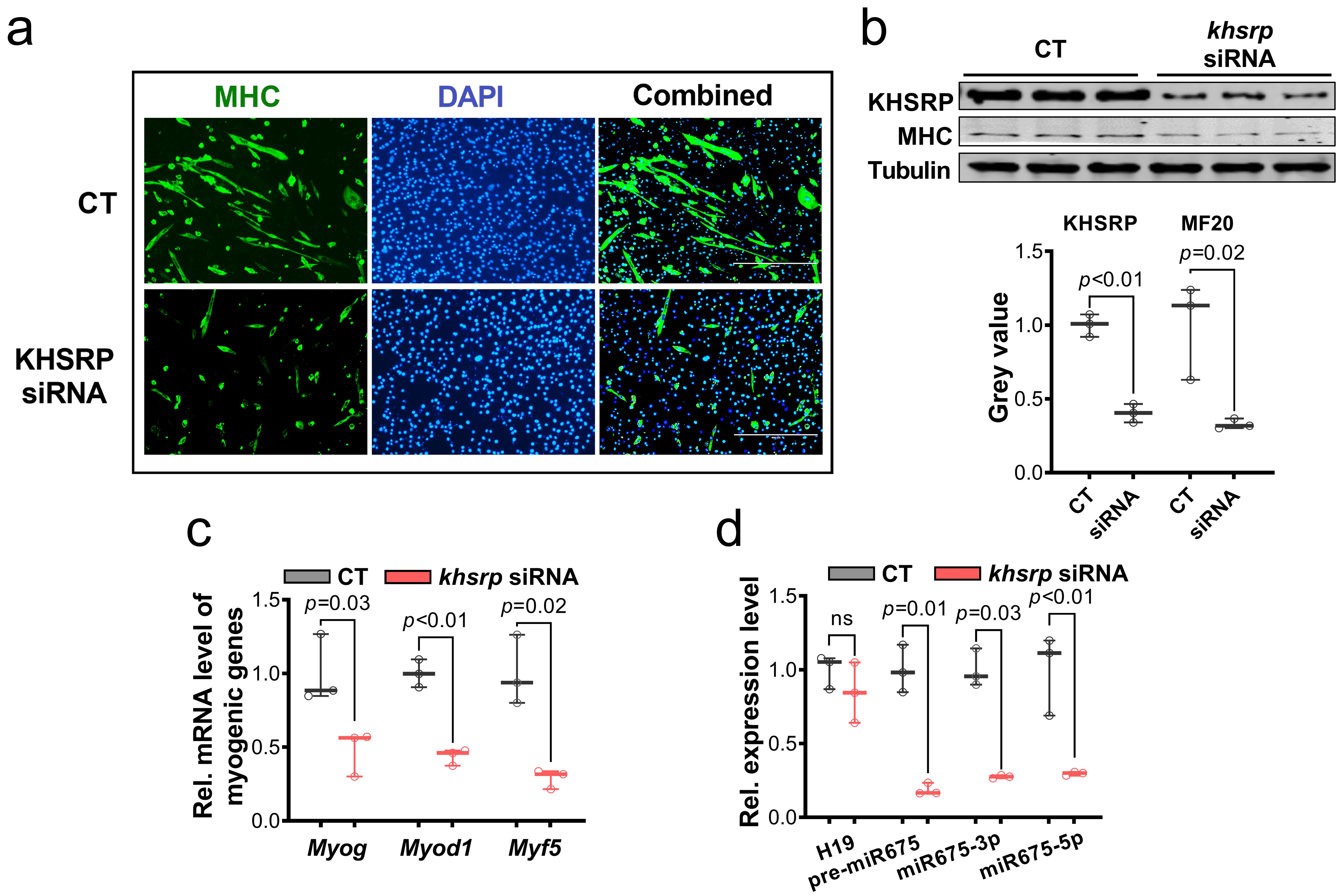

Fig. S8 | Knocking down KHSRP in P19 cells inhibits myogenesis and *miR675* biogenesis. a Representative images of ICC for MHC staining in P19 cells (5-day differentiation) from CT and *Khsrp* siRNA groups. **b** Representative images of western blots and quantification of KHSRP and MHC protein levels in P19 cells from CT and *Khsrp* siRNA groups (n=3). **c** Myogenic gene expression in P19 cells from CT and *Khsrp* siRNA groups (n=3). **d** Expression levels of *H19*, pre-*miR675*, *miR675-3p and miR675-5p* in P19 cells from CT and *Khsrp* siRNA groups (n=3). Data are presented as mean ± SD. *p* values from two-tailed unpaired Student's t test. CT, control; ICC: immunocytochemistry; siRNA, small interfering RNA; MHC, myosin heavy chain; DAPI, 4,6-diamidino-2-phenylindole.

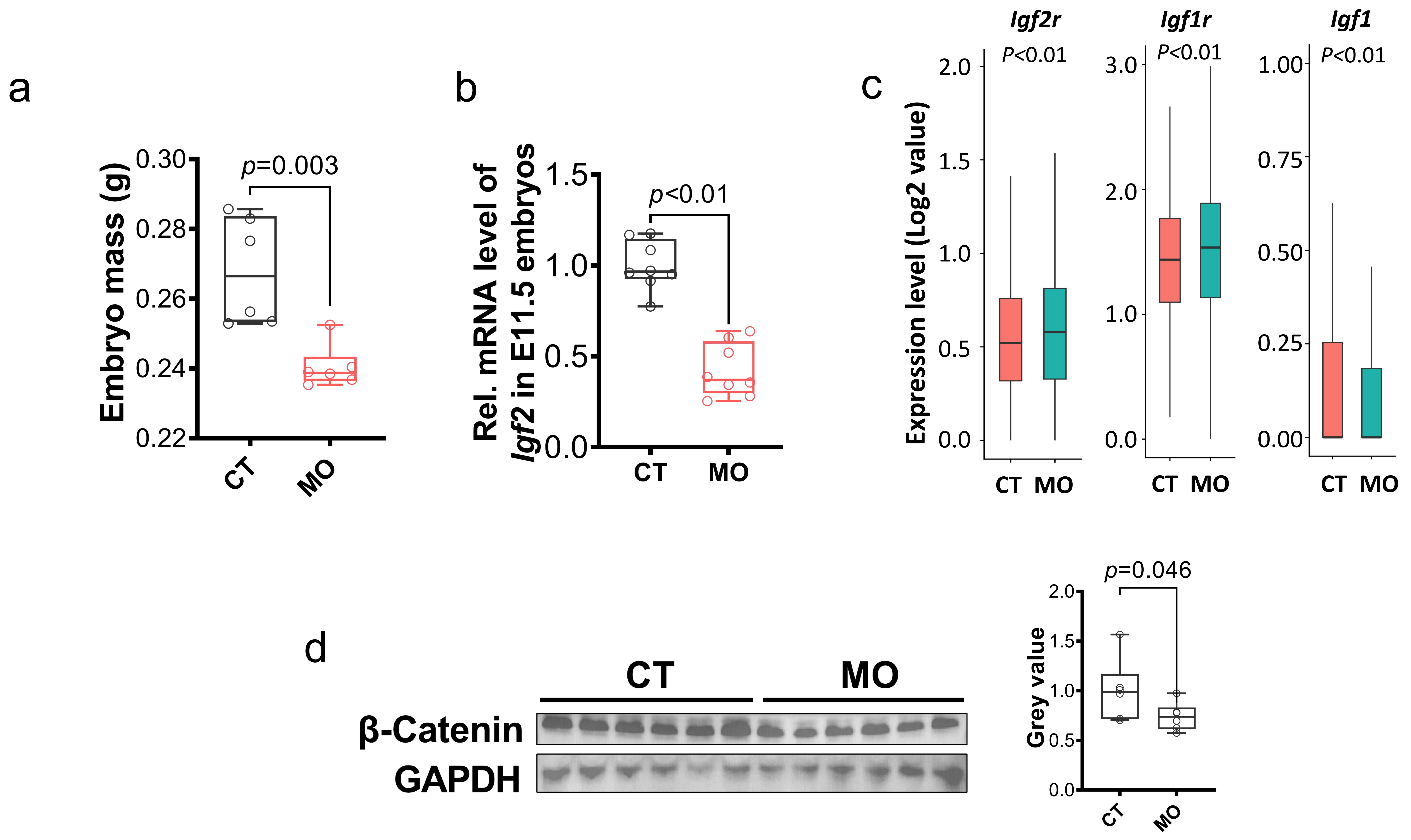

Fig. S9 | Maternal obesity inhibits embryonic growth and *Igf* signaling. a Embryo mass of E11.5 embryos from CT and MO groups (n=6). **b** Expression levels of *Igf2* in E11.5 embryos from CT and MO groups (n=8). **c** Expression level of *Igf2r*, *Igf1r* and *Igf1* in scRNA-seq data from myogenic cells cluster (E11.5 + E13.5). **d** Representative images of western blots and quantification of β-catenin protein levels in E11.5 embryos from CT and MO groups (n=6). Data are presented as mean ± SD; *p* values from two-tailed unpaired Student's t test. CT, control; MO, maternal obesity.

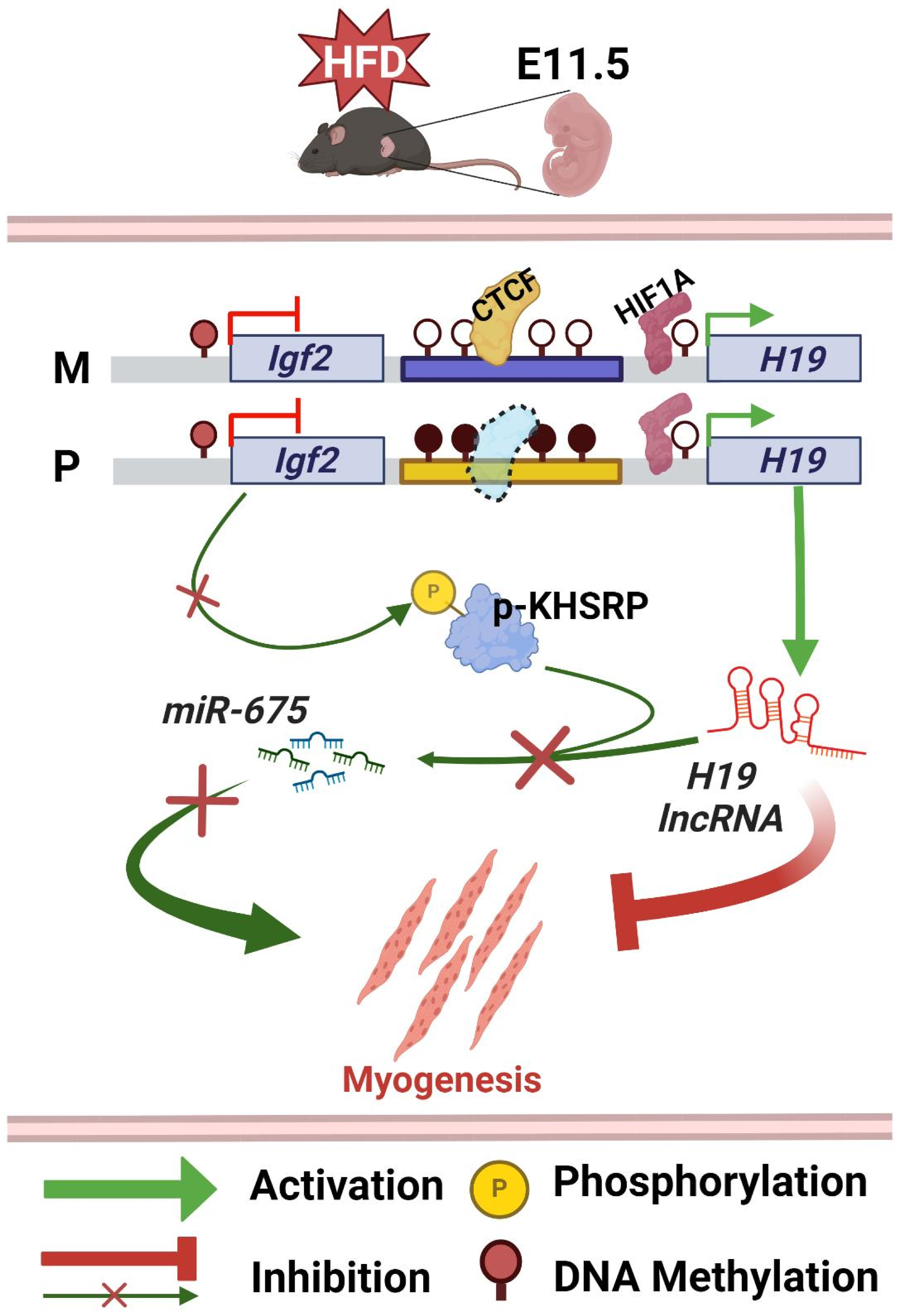

Fig. S10 | Summary schematic diagram. HFD-induced MO disrupts the imprinting of *H19/Igf2* locus, resulting in the diallelic expression of *H19* lncRNA and decreased *Igf2* expression. The decreased level of *Igf2* further reduced phosphorylation level of p-KHSRP. Consequently, KHSRP interacts with H19 and promotes myogenic mRNA decay, while its function of promoting miR675 biogenesis is blocked, which, in turn, deteriorates embryonic myogenesis. CT, control; HFD, high fat diet; lnc, long non-coding; MO, maternal obesity.
