# Supplementary material for: Balancing LncRNA H19 and miR‐675 Bioconversion as a Key Regulator of Embryonic Myogenesis Under Maternal Obesity
Source: J Cachexia Sarcopenia Muscle. 2025 Mar 31;16(2):e13791. doi: 10.1002/jcsm.13791 (PMC11955836; doi:10.1002/jcsm.13791)
Supplement: Supplementary file 4 — Data S4. Supporting Information. [file JCSM-16-e13791-s004.docx]

**Supplemental materials and methods**

**Immunoblotting and Immunocytochemical staining**

Protein samples were extracted as previously described [1]. The 10X Chromium Nuclei Isolation Kit (CG000505, 10x Genomics, CA) were used to extract cell nuclei. Primary antibodies against p-Serine (500-020, Thermo Fisher), KHSRP (MA5-35754, Thermo Fisher; ET7109-30, Huabio, Hong Kong), myosin heavy chain (MHC) (MF-20, DSHB, Iowa City, IA; HA722228, Huabio), HIF1A (PA1-16601, Thermo Fisher; HA721997, Huabio), AKT (AHO1112, Thermo Fisher), p-AKT (44-621G, Thermo Fisher), MYF5 (HA722400, Huabio), MyoD1 (ER1913-45, Huabio),β-Catenin (ET1601-5, Huabio), H2B (ET1612-25, Huabio), GAPDH (ET1601-4, Huabio) and Tubulin (AB_2315513, DSHB) were used. The secondary antibodies (No. 926-32,211, RRID: AB_621,843 and No. 926-68,070, RRID AB_10,956,588) were purchased from LI-COR Biosciences. Immunoblotting images were captured by an Odyssey Infrared Imaging System (LI-COR Biosciences) and quantified by ImageJ. The nuclei were extracted using NE-PER™ Nuclear and Cytoplasmic Extraction Reagents (78833, ThermoFisher).

Immunocytochemical staining was performed as previously described [1]. Primary antibody against embryonic myosin heavy chain (MHC, MF20, AB_2147781, DSHB) and fluorescent secondary antibody (6410-32, AB_2796308, Donkey anti-mouse, Southern Biotech, Birmingham, AL) were used. A mounting medium with DAPI (Abcam, Waltham, MA) was used and images were captured by EVOS XL Core Imaging System (Mil Creek, WA).

**Methylated DNA Immunoprecipitation (MeDIP) and immunoprecipitation (IP)**

Extracts were sonicated by a sonic dismembrator (Fisher scientific) as follows: 30% intensity for 230 s with 5s ON/5s OFF. The optimal size (200-1000 bp) of sonicated DNA was verified by agarose gel electrophoresis. Then, 5mC primary antibody (ab53729, Abcam) or IgG (negative control, Sigma) was added, and immunoprecipitation (IP) was performed using EcoMagTM Protein A Magnetic Particles (#MJA-102, Bioclone Inc., San Diego, CA). The products were further analyzed by PCR. The primers used are listed in Supplementary Table 1. Only 2 pairs of primers for HIF1A binding sites and 1 pair of primers for IGF2 P2 were designed because some of the sites are adjacent. Similar IP protocol was followed using antibody against p-Serine (500-020, Thermo Fisher) to analyze the phosphorylation level of KHSRP.

**Cell culture and treatments**

An embryonic cell line, P19 (CRL-1825, ATCC), was used to examine embryonic myogenesis, as previously described [2]. Briefly, P19 cells were cultured in an alpha minimal essential growth medium containing 7.5% bovine calf serum, 2.5% fetal bovine serum, and 0.5% penicillin streptomycin (Sigma, Burlington, MA) in tissue culture dishes (37°C, 5% CO_2_). At ~70% confluency, a total of 5 × 10^6^ P19 cells were seeded onto a petri dish (100 mm, VWR International, Irving, TX) with growth medium and 1% dimethyl sulfoxide to induce the formation of embryoid bodies [3]. To prevent embryoid bodies from adhering to the bottom, petri dishes were placed in an incubator with shaking (Incu-Shaker Mini, Benchmark, Woburn, MA) at 70 rpm for 4 days. The embryoid bodies were transferred back to tissue culture dishes and kept in adherent culture until confluency. The induction of myogenic differentiation was achieved by adding 1% dimethyl sulfoxide into the culture medium [2]. Medium was replaced every two days. In total, it takes about 10 - 12 days to observe the aligned myofibers after myogenic induction.

To analyze the effects of hypoxia, a hypoxic chamber connected with low oxygen (1% oxygen, 5% CO_2_, and 90% nitrogen) gas mixture was used to culture the P19 cells for 24 h during its myogenic differentiation [4]. In addition, 25 ng/ml of Recombinant Mouse IGF2 protein (792-MG-050, Bio-techne, Minneapolis, MN) [5] or 100 nM of Akt inhibitor PHT427 (4598/10, Bio-techne) was applied for corresponding experiments [6].

**Gene gain and loss function analysis**

To manipulate *H19* expression in P19 cells, dead CRISPR-associated protein 9 (dCas9) system was applied (Fig. S5a) [7]. The dCas9-VP64_Blast activation vector (61425, Addgene, Watertown, MA) and dCas9/KRAB/MeCP2 inhibition vector (VB900121-95577XHD, VectorBuilder, Chicago, IL, USA) were utilized to overexpress and inhibit the expression of *H19* in cells, respectively. Customized pLenti_sgRNA(MS2)_zeo (VectorBuilder Inc.) containing small guide RNAs (sgRNAs) targeting *H19* promoter (Supplementary Table 1), and coactivator vector pLenti_MS2-P65-HSF1_Hygro (61426, Addgene) were also transfected.

The sgRNAs were designed using CHOPCHOP v3 [8, 9]. The *H19* gene sequence is based on NC_000073.7 (Chr7:142129267-142131883 bp, - strand) and MGP_CASTEiJ_G0007116 (Chr7:139668992-139673525 bp, -strand). The TrueGuide™ sgRNA (A35526, Thermo Fisher) was used as a negative control.

All vectors were packed into lentivirus using 293T cells (CRL-3216, ATCC) by Lipofectamine 3000 (L3000008, Invitrogen, Lenexa, KS) transfection. P19 cells were then transfected by packaged lentivirus to select stable *H19* overexpressed and knockdown cell lines.

For RNA interference, KHSRP siRNA (4390771, Thermo Fisher) was transfected into cells to inhibit the expression of KHSRP, and generic Stealth RNAi™ siRNA was used as a negative control (12935300, Thermo Fisher). The mirVana™ miR675 inhibitor (MH11780, Thermo Fisher) and mirVana™ miR675 mimic (MC11780, Thermo Fisher) were used to decrease and increase the *miR675-3p*, respectively. The untargeted miR inhibitor/mimic served as negative controls (4464058, Thermo Fisher).

**RNA quantification**

For mRNA quantification, total RNA was obtained using TRIzol reagent (15596018, Invitrogen). Complementary DNA (cDNA) was reversely transcribed from RNA by an iScriptTM cDNA Synthesis Kit (Bio-Rad, Hercules, CA). To perform PCR, SsoAdvanced^TM^ Universal SYBR Green Supermix (BioRad, Hercules, CA) was used, and a Real-time System (Bio-Rad) was used to run the reaction and visualize the signal. 18S rRNA was used as a reference gene and relative RNA levels were calculated by the 2-∆∆Ct method [10].

To quantify small RNAs, including *pri-miR675*, *pre-miR675* and *miR675*, NucleoSpin® miRNA (740971, Takara Bio Inc., San Jose, CA) was used to extract the small RNAs (<200 nt). Mir-X™ miRNA qRT-PCR TB Green® Kit (638315, Takara) was used to synthesize cDNA from total small RNAs extracted above, as described in the manual. Because noncoding RNAs are poly(A)-tailed, they were amplified using a modified oligo(dT) primer and a target specific primer (Supplementary table 1).

To analyze the accurate expression level of mature *miR675*, TaqMan™ MicroRNA Reverse Transcription Kit (4366596, Thermo Fisher) was used. This method specifically reverse transcribed the *miR675* with a stem-looped primer, and then amplified the stem-looped cDNA by TaqMan™ Universal Master Mix II (4440042, Thermo Fisher) using a target-specific primer [11].

A Real-time System (Bio-Rad) was used to run the reactions and visualize the signals. U6 TaqMan® Assays and Arrays (4427975, Thermo Fisher) was used as an internal control gene. All the sequences of primers mentioned above are listed in Supplementary Table 1.

***H19* allelic expression analysis**

To analyze allelic expression of *H19*, CAST/EiJ (E; 000928, The Jackson Lab, Bar Harbor, Maine) mice were crossed with C57BL/6 (B), resulting in 8 single nucleotide polymorphisms (SNPs) between two alleles in the *H19* gene (Fig. S6). A pair of primers (Supplementary Table1) was designed to generate an amplicon where an AlwNI (R0514S, New England Biolabs, Ipswich, MA) restriction site is present in the CAST/EiJ allele but not in the C57BL/6J allele. Following PCR, the amplicons were incubated with restriction enzyme AlwNI (R0514S, New England Biolabs, Ipswich, MA), and fragments were visualized by agarose gel electrophoresis.

**References**

1. Zhao L, Law NC, Gomez NA, Son J, Gao Y, Liu X, et al. Obesity impairs embryonic myogenesis by enhancing BMP signaling within the dermomyotome. Advanced Science. 2021;8:2102157.

2. Gao Y, Zhao L, Son JS, Liu X, Chen Y, Deavila JM, et al. Maternal exercise before and during pregnancy facilitates embryonic myogenesis by enhancing thyroid hormone signaling. Thyroid. 2022;32:581-93.

3. Wang B, Fu X, Liang X, Deavila JM, Wang Z, Zhao L, et al. Retinoic acid induces white adipose tissue browning by increasing adipose vascularity and inducing beige adipogenesis of PDGFRα+ adipose progenitors. Cell discovery. 2017;3:1-14.

4. Pavlacky J, Polak J. Technical feasibility and physiological relevance of hypoxic cell culture models. Frontiers in endocrinology. 2020;11:57.

5. Wan X, Helman LJ. Effect of insulin-like growth factor II on protecting myoblast cells against cisplatin-induced apoptosis through p70 S6 kinase pathway. Neoplasia. 2002;4:400-8.

6. Ortega I, Villanueva JA, Wong DH, Cress AB, Sokalska A, Stanley SD, et al. Resveratrol reduces steroidogenesis in rat ovarian theca-interstitial cells: the role of inhibition of Akt/PKB signaling pathway. Endocrinology. 2012;153:4019-29.

7. Chen M, Qi LS. Repurposing CRISPR system for transcriptional activation. RNA Activation. 2017;147-57.

8. Labun K, Montague TG, Krause M, Torres Cleuren YN, Tjeldnes H, Valen E. CHOPCHOP v3: expanding the CRISPR web toolbox beyond genome editing. Nucleic acids research. 2019;47:W171-W4.

9. Labun K, Montague TG, Gagnon JA, Thyme SB, Valen E. CHOPCHOP v2: a web tool for the next generation of CRISPR genome engineering. Nucleic acids research. 2016;44:W272-W6.

10. Schmittgen TD, Livak KJ. Analyzing real-time PCR data by the comparative CT method. Nature protocols. 2008;3:1101-8.

11. Chen C, Ridzon DA, Broomer AJ, Zhou Z, Lee DH, Nguyen JT, et al. Real-time quantification of microRNAs by stem–loop RT–PCR. Nucleic acids research. 2005;33:e179-e.
