# Supplementary material for: Balancing LncRNA H19 and miR‐675 Bioconversion as a Key Regulator of Embryonic Myogenesis Under Maternal Obesity
Source: J Cachexia Sarcopenia Muscle. 2025 Mar 31;16(2):e13791. doi: 10.1002/jcsm.13791 (PMC11955836; doi:10.1002/jcsm.13791)

Uncropped membrane and gel

Fig. 3c

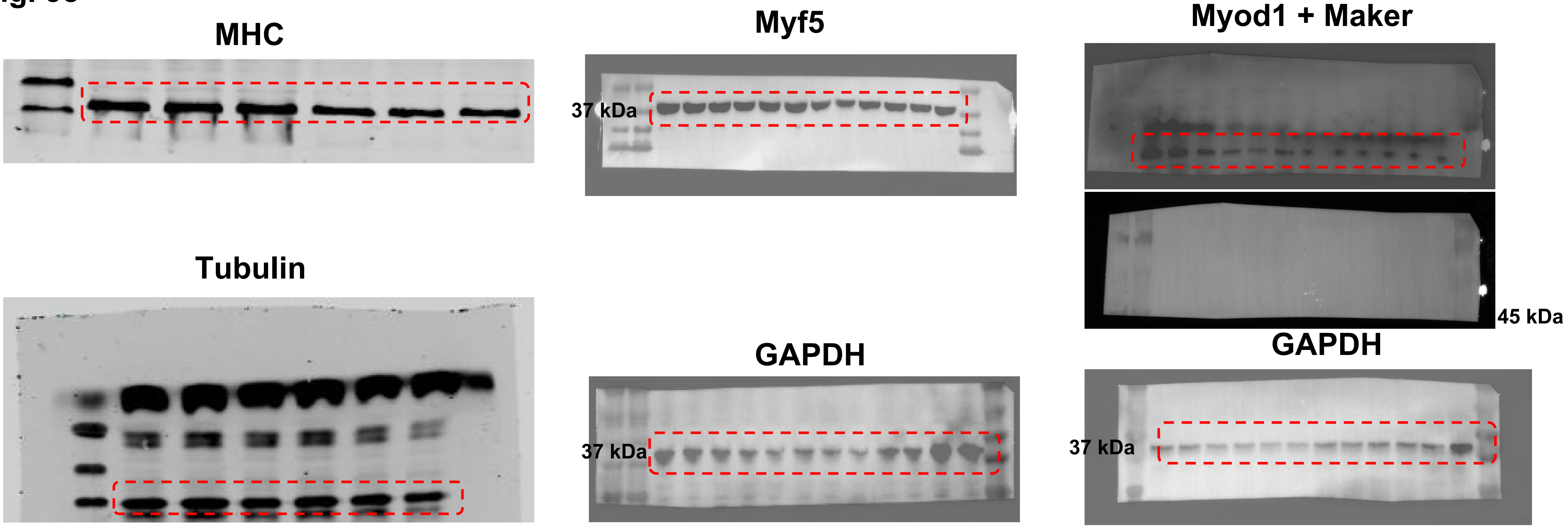

Fig. 4e

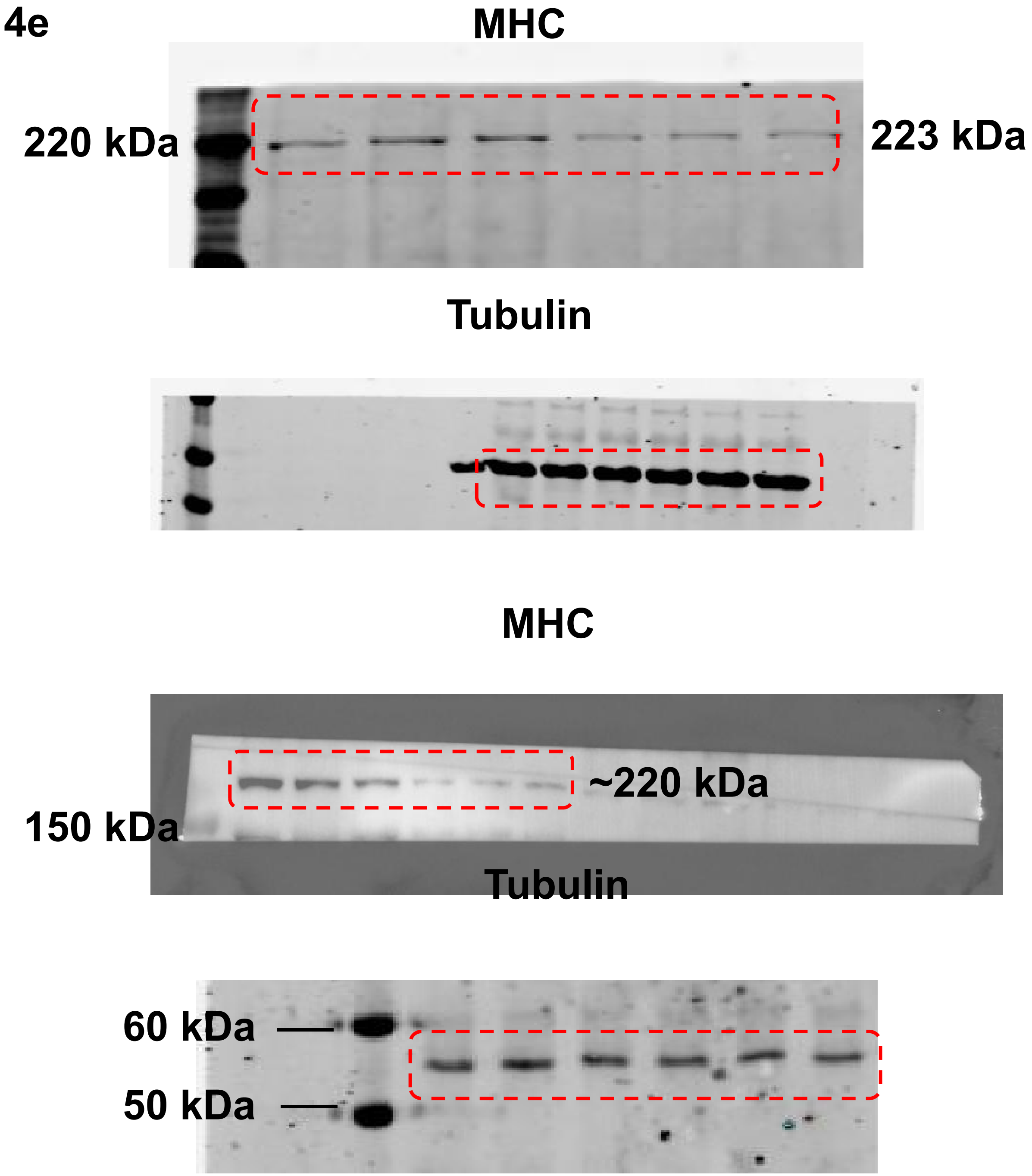
MHC
220 kDa
223 kDa
Tubulin
MHC
~220 kDa
150 kDa
Tubulin
60 kDa
50 kDa

Fig. 6b

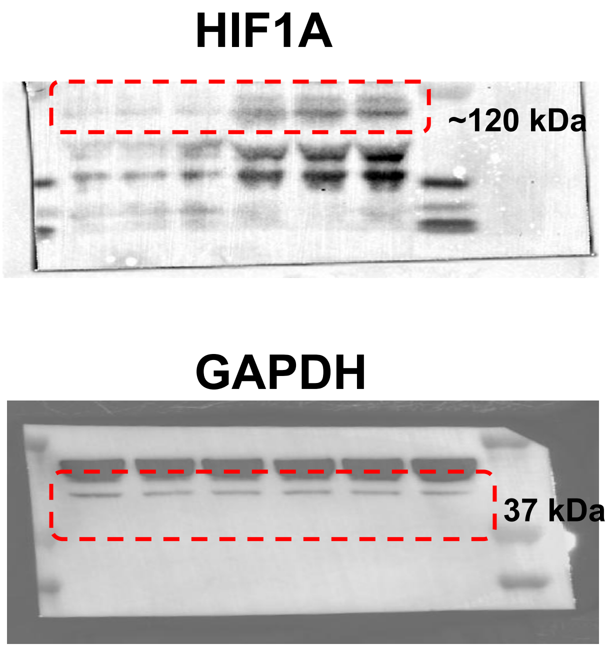

Fig. 6h

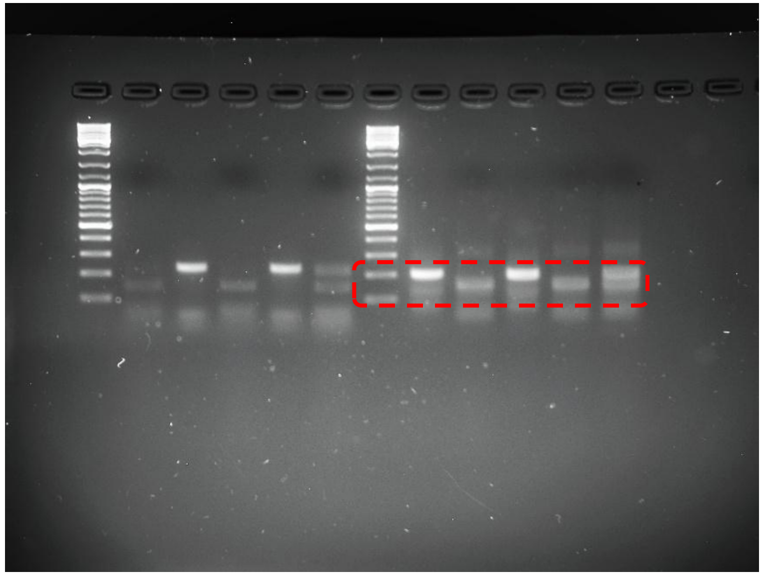

Fig. 7c

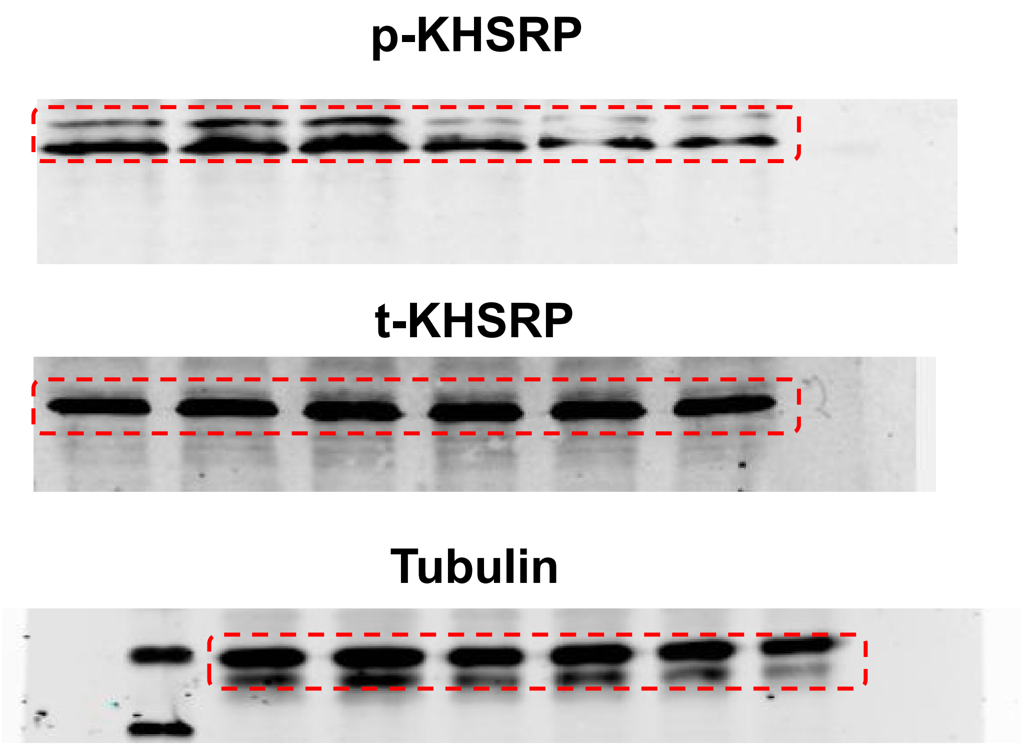

Fig. 7d

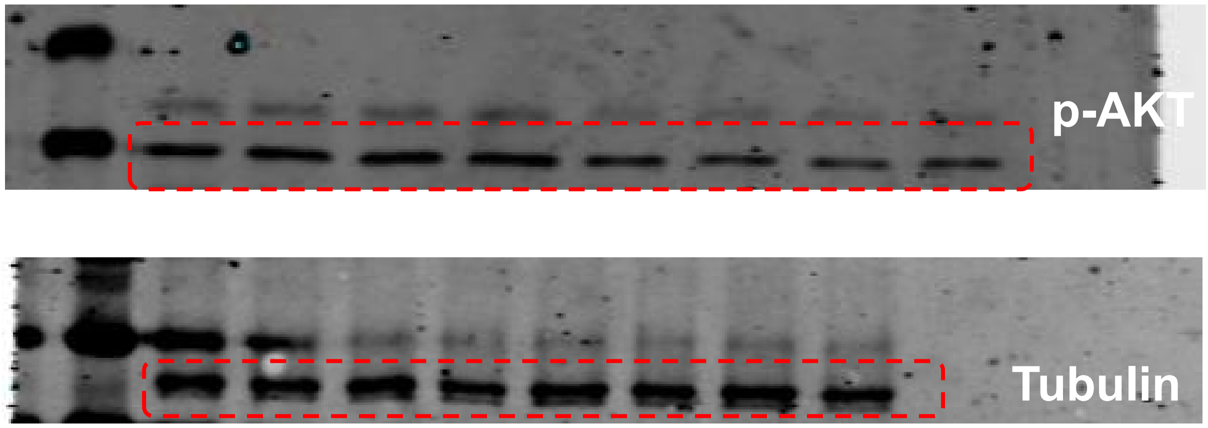

Fig. 8h

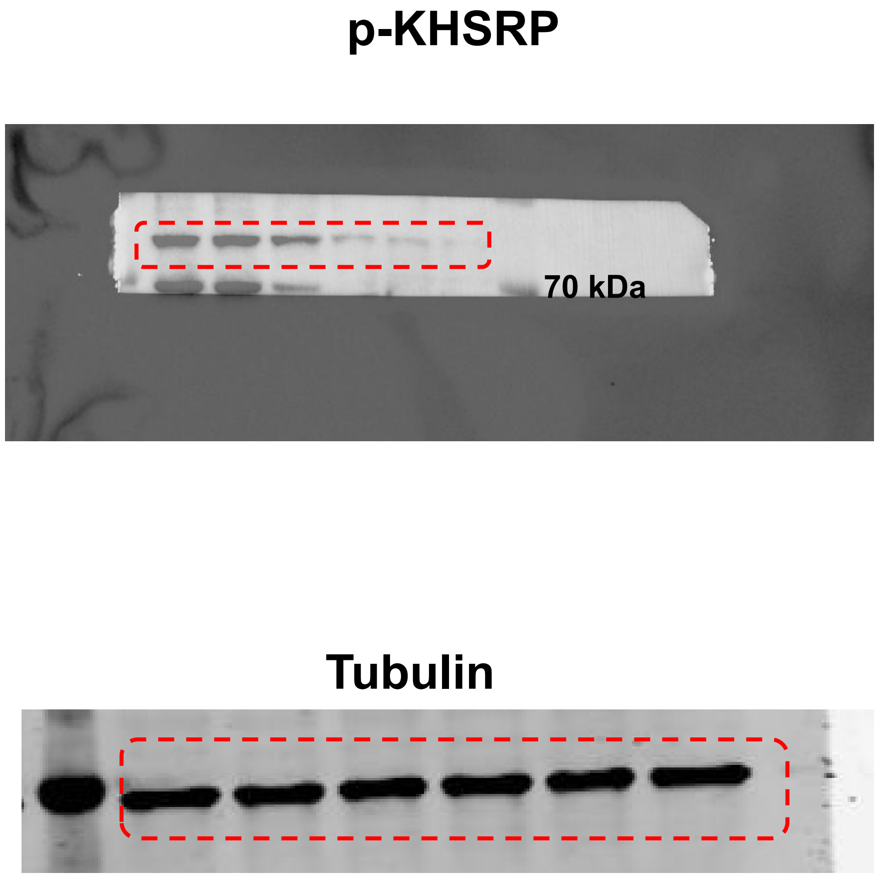

Fig. 8i

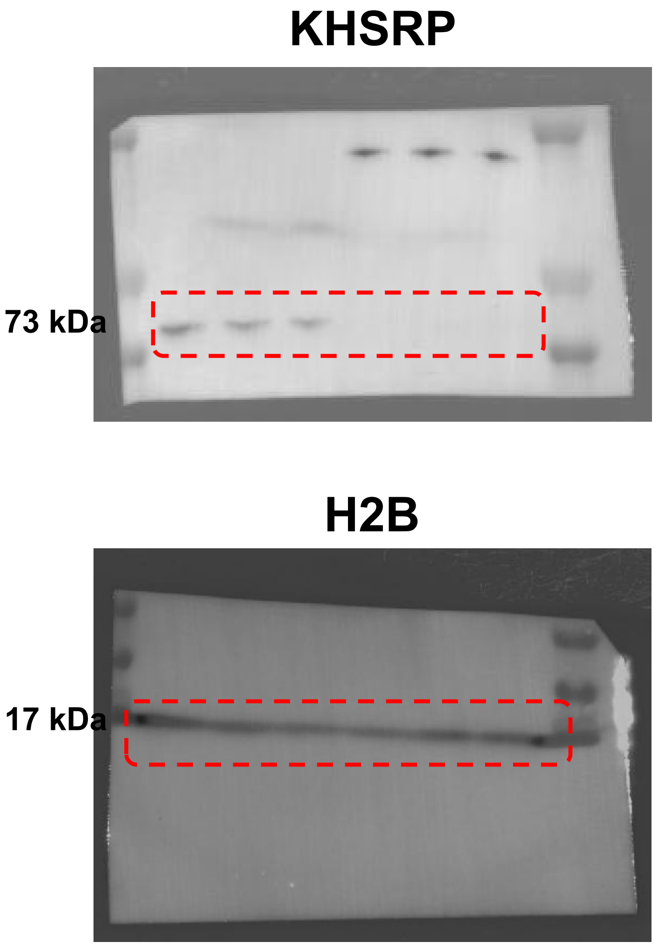

Fig. S7a

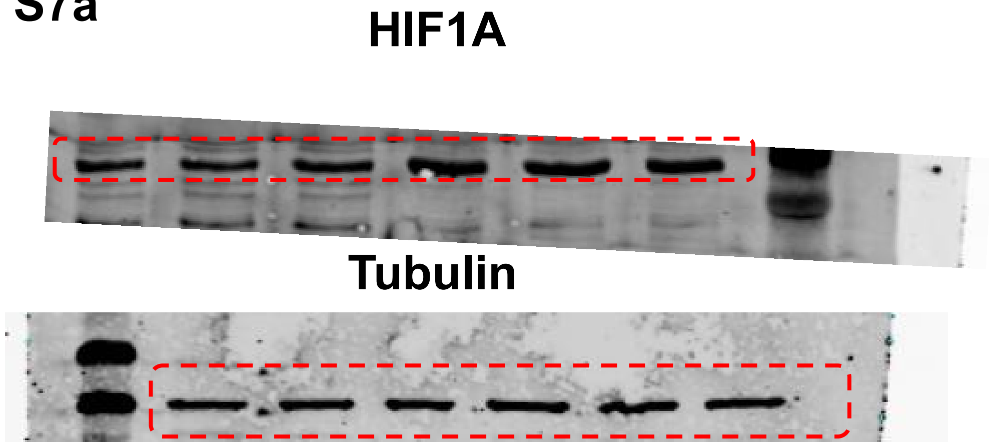

Fig. S7d

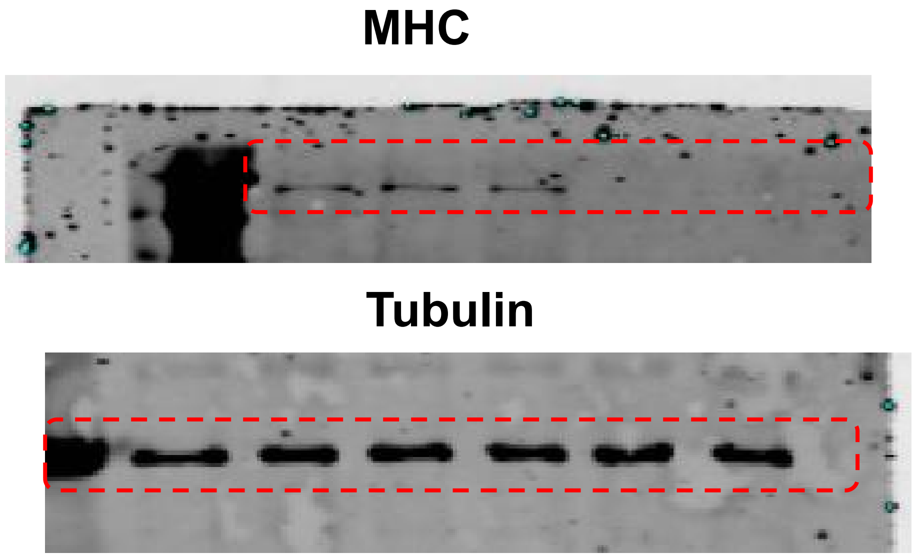

Fig. S8b

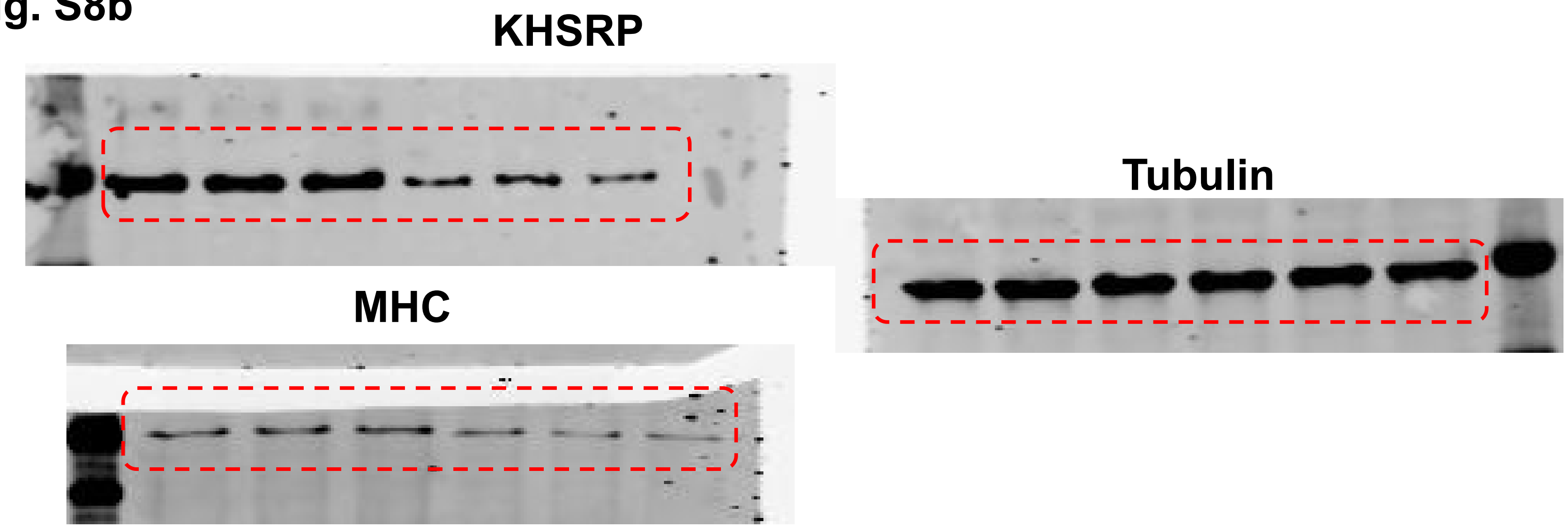

Fig. S9d

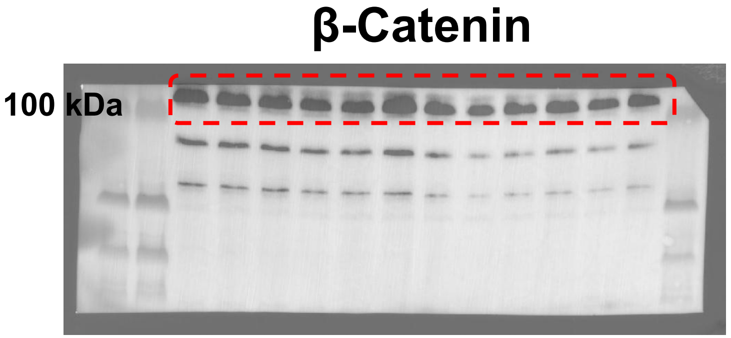

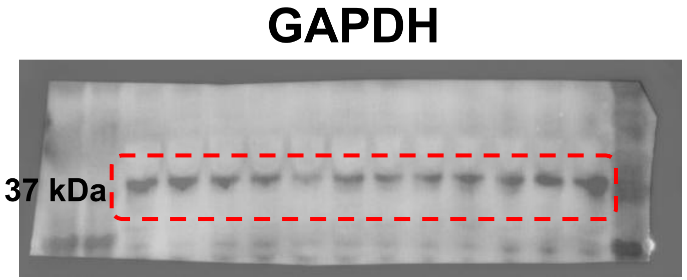

Supplement: Supplementary file 5 — Data S5. Supporting Information. [file JCSM-16-e13791-s002.pdf]
