# Supplementary material for: Balancing LncRNA H19 and miR‐675 Bioconversion as a Key Regulator of Embryonic Myogenesis Under Maternal Obesity
Source: J Cachexia Sarcopenia Muscle. 2025 Mar 31;16(2):e13791. doi: 10.1002/jcsm.13791 (PMC11955836; doi:10.1002/jcsm.13791)
Supplement: Supplementary file 7 — Data S7. Supporting Information. [file JCSM-16-e13791-s003.pdf]

Fragment Analyzer Run Summary:

Filename and Data Path: C:\AATI\Data\2022 10 05\100522-Deqiang 10-50-03\2022 10 05 10H 50M.raw
Created: Wednesday, October 05, 2022 11:05:58 AM
# of Capillaries: 12
Array Serial #: 082720-13SFS
Effect Length: 33 cm
Array Usage Count: 403
FA Version #: 1.1.0.11
Device Serial #: 2821

METHOD INFORMATION
Method Name: DNF-474-33 - HS NGS Fragment 1-6000bp.mthds
Gel Prime: No
Full Conditioning: Yes
Gel Prime to Bufer: No
Gel Selection: Gel 2
Perform Prerun: 6.0 kV, 30 sec.
Rinse: No
Marker 1: No
Rinse: Tray: 3, Row: G, # Dips: 1
Sample Injection: 5.0 kV, 30 sec.
Separation: 6.0 kV, 50.0 min.
Tray Name: 093022-Deqiang-Derek

Analysis Mode: NGS

NOTE

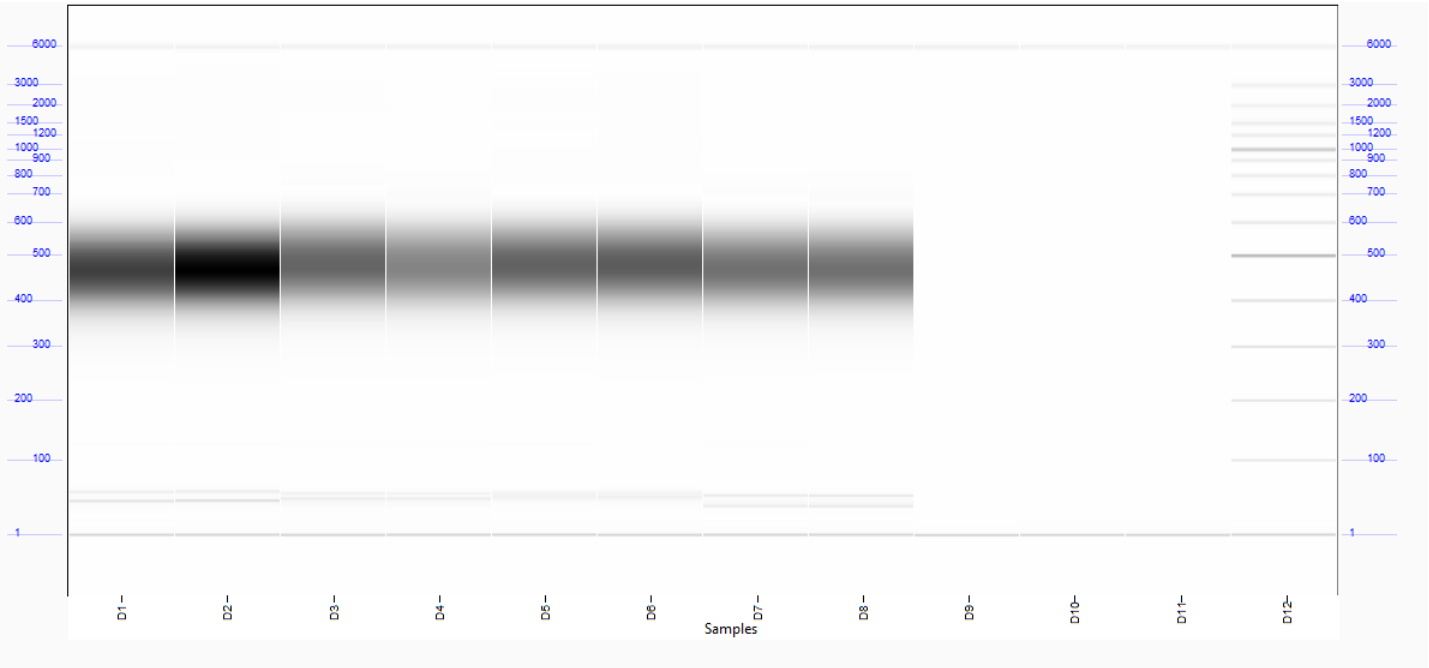

Copyright 2013 Advanced Analytical Technologies, Inc.

Filename and Data Path: C:\AATI\Data\2022 10 05\100522-Deqiang 10-50-03\2022 10 05 10H 50M.raw

D1: Deqiang-c1B

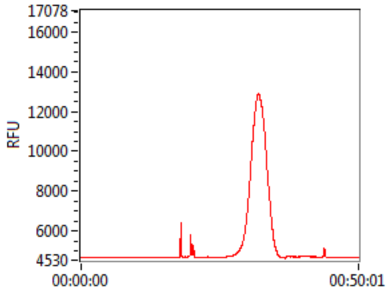

D2: Deqiang-C1B

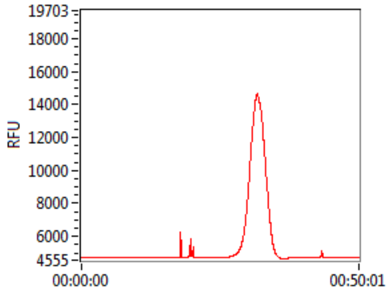

D3: Deqiang-C2B

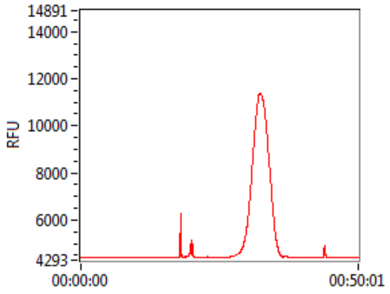

D4: Deqiang-C2B

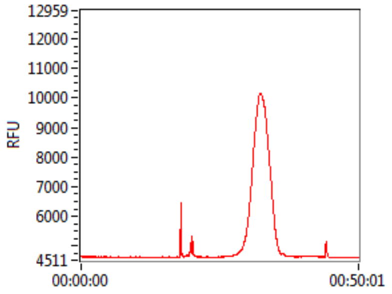

D5: Deqiang-H1B

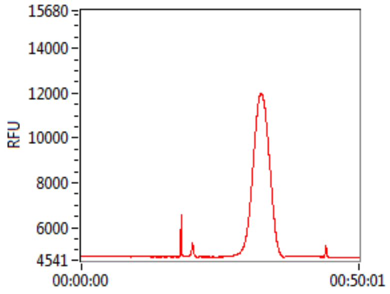

D6: Deqiang-H1B

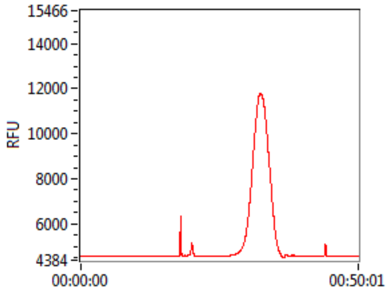

D7: Deqiang-H2B

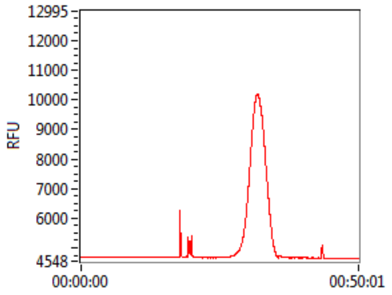

D8: Deqiang-H2B

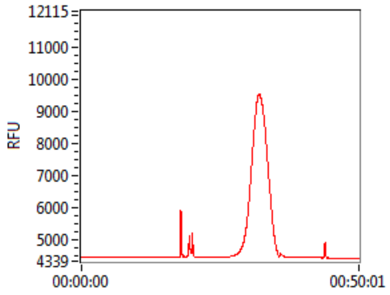

D9: SampD9

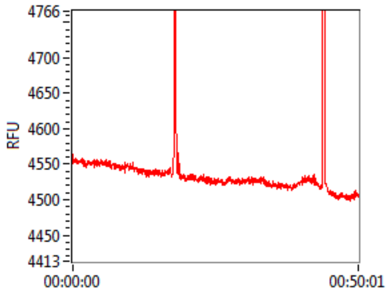

D10: SampD10

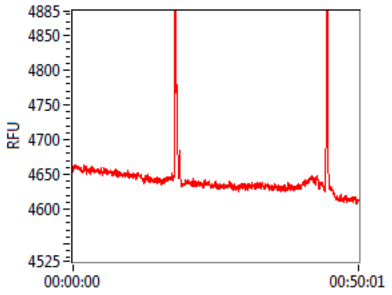

D11: SampD11

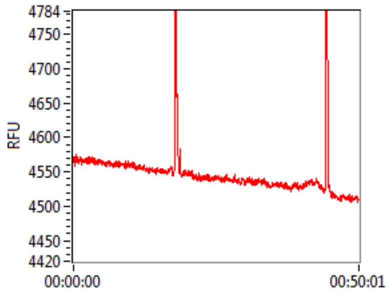

D12: ladder

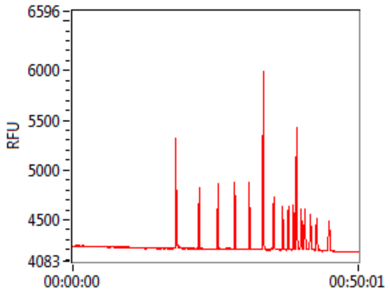

Copyright 2013 Advanced Analytical Technologies, Inc.

Sample: Deqiang-c1B
Well Location: D1
Created: Wednesday, October 05, 2022 11:05:58 AM

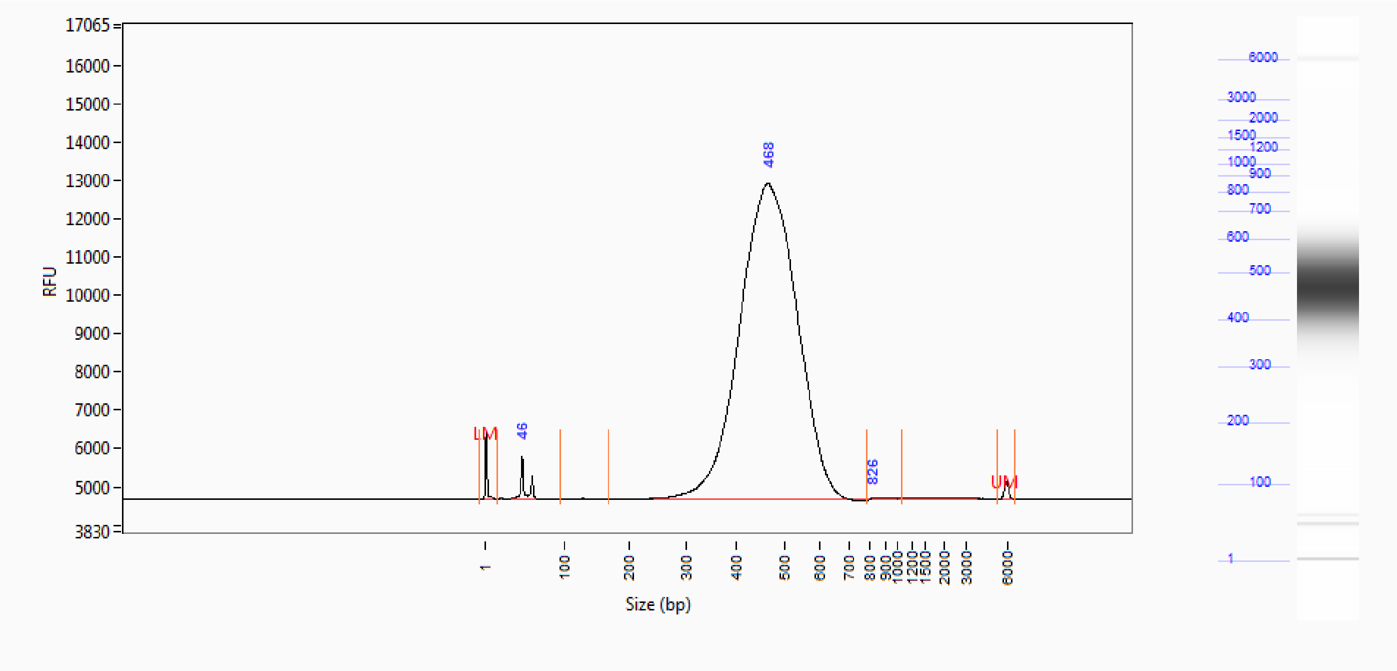

Peak	Size (bp)	Conc. (ng/uL)	Rel. Conc. %	Molarity (nmole/L)	From (bp)	CV%
1	1 (LM)	0.0133		17.353	0	163.22
2	46	0.2223	1.6	7.845	16	18.68
3	468	13.6198	98.3	47.920	168	13.10
4	826	0.0160	0.1	0.032	790	7.81
5	6000 (UM)	0.0037		0.001	5362	2.83

TIC:	13.8581	ng/uL
TIM:	55.796	nmole/L
Total Conc.:	13.9063	ng/uL

Sample Peak Width (sec): 50 Sample Min Peak Height: 25 Sample Baseline V to V?: Y Sample Baseline V to V pts: 3
Sample Filter: Binomial # of Pts for Filter: 3 Sample Start Region (min): 0 Sample End Region (min): 50
Manual Baseline Start (min): 10 Manual Baseline End (min): 48
Marker Peak Width (sec): 5 Marker Min Peak Height: 200 Marker Baseline V to V?: Y Marker Baseline V to V pts: 3
Lower Marker Selection: First Peak > 200 RFU Upper Marker Selection: Last Peak > 200 RFU
Ladder Size (bp): 1, 100, 200, 300, 400, 500, 600, 700, 800, 900, 1000, 1200, 1500, 2000, 3000, 6000
Quantification Using: Ladder Final Concentration (ng/uL): 0.0830 Dilution Factor: 12.0

 Copyright 2013 Advanced Analytical Technologies, Inc.

Sample: Deqiang-C1B
Well Location: D2
Created: Wednesday, October 05, 2022 11:05:58 AM

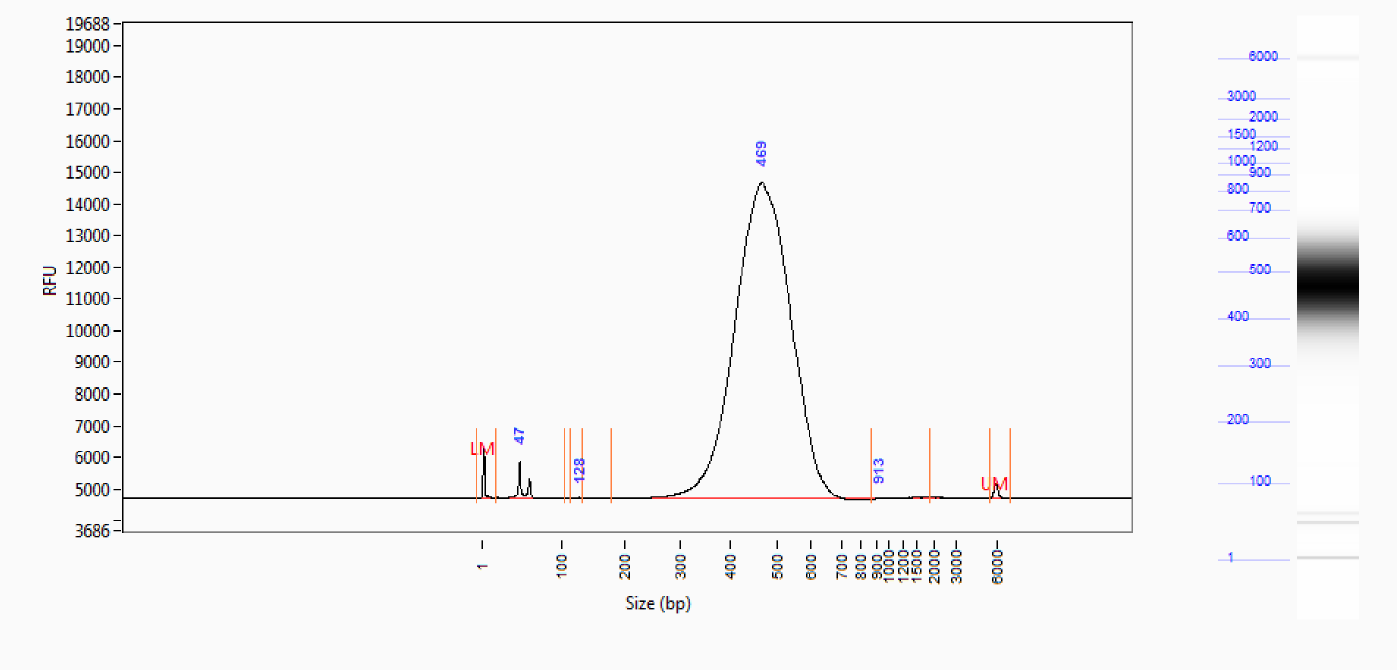

Peak	Size (bp)	Conc. (ng/uL)	Rel. Conc. %	Molarity (nmole/L)	From (bp)	CV%
1	1 (LM)	0.0133		17.353	0	160.16
2	47	0.2491	1.4	8.715	17	19.64
3	128	0.0050	0.0	0.065	116	2.25
4	469	16.9866	98.3	59.593	180	12.99
5	913	0.0334	0.2	0.060	865	22.36
6	6000 (UM)	0.0038		0.001	5551	3.38

TIC:	17.2741	ng/uL
TIM:	68.433	nmole/L
Total Conc.:	17.3054	ng/uL

```
Sample Peak Width (sec): 50   Sample Min Peak Height: 25    Sample Baseline V to V?: Y    Sample Baseline V to V pts: 3
Sample Filter: Binomial       # of Pts for Filter: 3        Sample Start Region (min): 0  Sample End Region (min): 50
Manual Baseline Start (min): 10     Manual Baseline End (min): 48
Marker Peak Width (sec): 5    Marker Min Peak Height: 200   Marker Baseline V to V?: Y    Marker Baseline V to V pts: 3
Lower Marker Selection: First Peak > 200 RFU                Upper Marker Selection: Last Peak > 200 RFU
Ladder Size (bp): 1, 100, 200, 300, 400, 500, 600, 700, 800, 900, 1000, 1200, 1500, 2000, 3000, 6000
Quantification Using: Ladder          Final Concentration (ng/uL): 0.0830          Dilution Factor: 12.0
```

 Copyright 2013 Advanced Analytical Technologies, Inc.

Sample: Deqiang-C2B
Well Location: D3
Created: Wednesday, October 05, 2022 11:05:58 AM

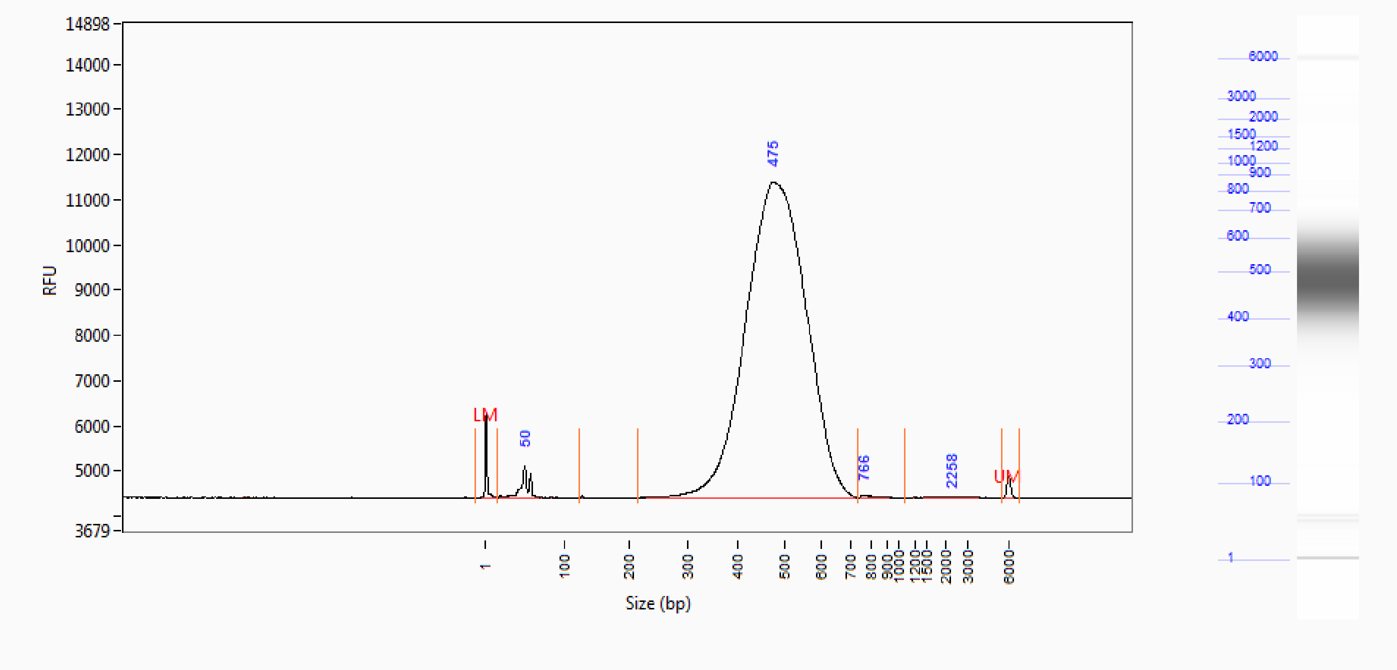

Peak	Size (bp)	Conc. (ng/uL)	Rel. Conc. %	Molarity (nmole/L)	From (bp)	CV%
1	1 (LM)	0.0133		17.353	0	196.09
2	50	0.2173	1.9	7.177	15	20.76
3	475	11.1497	97.6	38.618	215	13.43
4	766	0.0240	0.2	0.052	742	8.90
5	2258	0.0281	0.2	0.020	1088	40.28
6	6000 (UM)	0.0034		0.001	5528	2.51

TIC:	11.4192	ng/uL
TIM:	45.866	nmole/L
Total Conc.:	11.4236	ng/uL

Sample Peak Width (sec): 50 Sample Min Peak Height: 25 Sample Baseline V to V?: Y Sample Baseline V to V pts: 3
Sample Filter: Binomial # of Pts for Filter: 3 Sample Start Region (min): 0 Sample End Region (min): 50
Manual Baseline Start (min): 10 Manual Baseline End (min): 48
Marker Peak Width (sec): 5 Marker Min Peak Height: 200 Marker Baseline V to V?: Y Marker Baseline V to V pts: 3
Lower Marker Selection: First Peak > 200 RFU Upper Marker Selection: Last Peak > 200 RFU
Ladder Size (bp): 1, 100, 200, 300, 400, 500, 600, 700, 800, 900, 1000, 1200, 1500, 2000, 3000, 6000
Quantification Using: Ladder Final Concentration (ng/uL): 0.0830 Dilution Factor: 12.0

 Copyright 2013 Advanced Analytical Technologies, Inc.

Sample: Deqiang-C2B
Well Location: D4
Created: Wednesday, October 05, 2022 11:05:58 AM

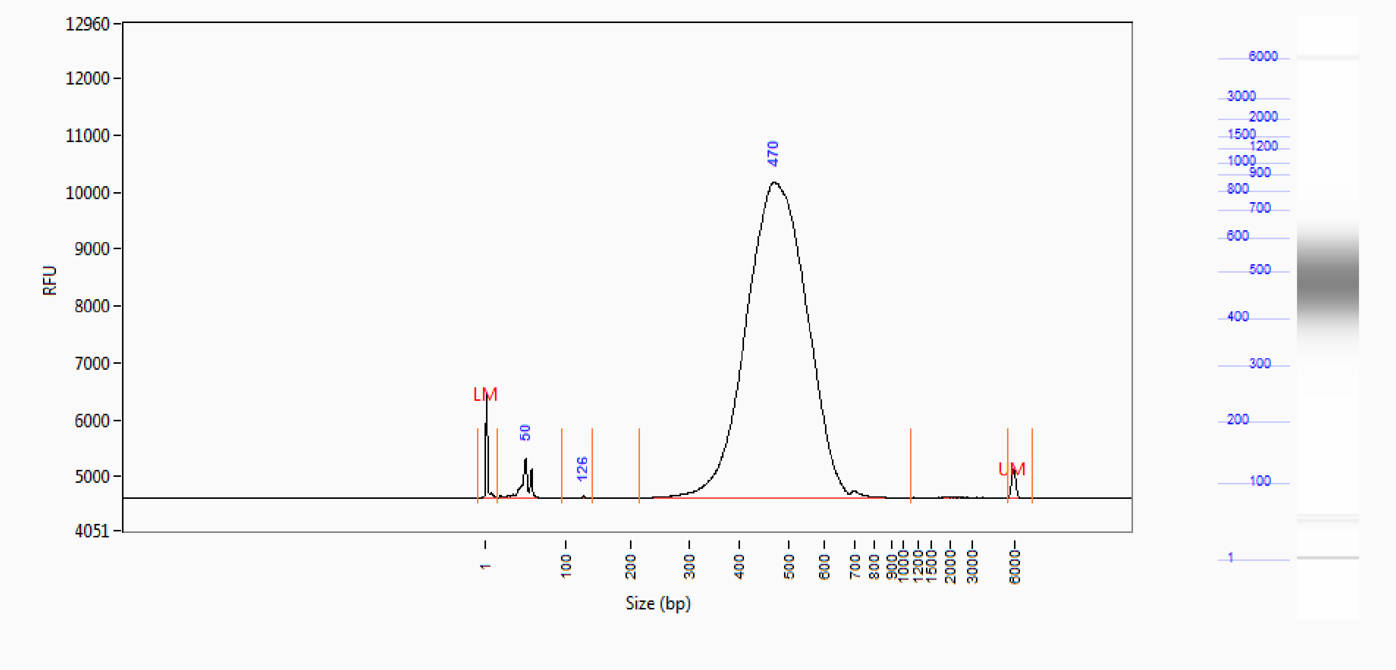

Peak	Size (bp)	Conc. (ng/uL)	Rel. Conc. %	Molarity (nmole/L)	From (bp)	CV%
1	1 (LM)	0.0133		17.353	0	160.49
2	50	0.2135	2.4	7.050	15	21.52
3	126	0.0042	0.0	0.055	94	5.91
4	470	8.8053	97.6	30.803	215	14.04
5	6000 (UM)	0.0036		0.001	5528	3.22

TIC:	9.0231	ng/uL
TIM:	37.909	nmole/L
Total Conc.:	9.0515	ng/uL

```
Sample Peak Width (sec): 50   Sample Min Peak Height: 25    Sample Baseline V to V?: Y    Sample Baseline V to V pts: 3
Sample Filter: Binomial       # of Pts for Filter: 3        Sample Start Region (min): 0  Sample End Region (min): 50
Manual Baseline Start (min): 10     Manual Baseline End (min): 48
Marker Peak Width (sec): 5    Marker Min Peak Height: 200   Marker Baseline V to V?: Y    Marker Baseline V to V pts: 3
Lower Marker Selection: First Peak > 200 RFU                Upper Marker Selection: Last Peak > 200 RFU
Ladder Size (bp): 1, 100, 200, 300, 400, 500, 600, 700, 800, 900, 1000, 1200, 1500, 2000, 3000, 6000
Quantification Using: Ladder          Final Concentration (ng/uL): 0.0830          Dilution Factor: 12.0
```


Copyright 2013 Advanced Analytical Technologies, Inc.

Sample: Deqiang-H1B
Well Location: D5
Created: Wednesday, October 05, 2022 11:05:58 AM

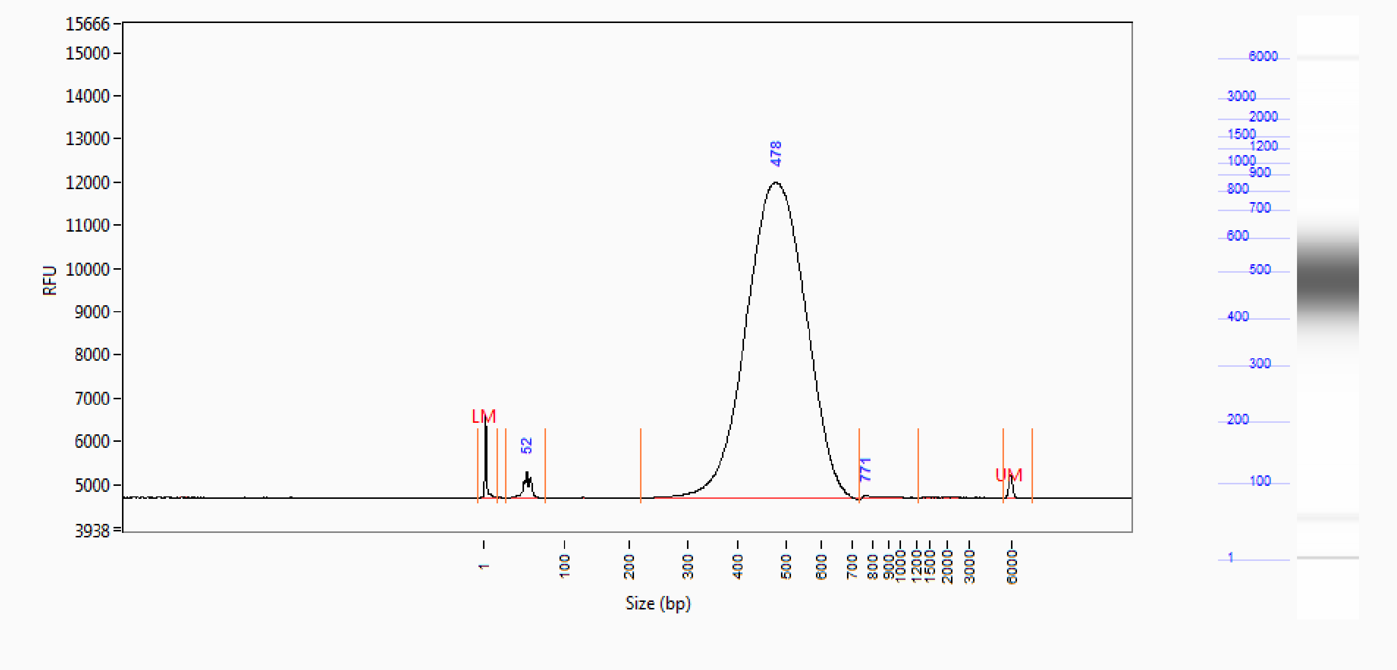

Peak	Size (bp)	Conc. (ng/uL)	Rel. Conc. %	Molarity (nmole/L)	From (bp)	CV%
1	1 (LM)	0.0133		17.353	0	150.06
2	52	0.1635	1.5	5.111	28	11.46
3	478	10.7708	98.2	37.043	220	13.10
4	771	0.0317	0.3	0.068	743	14.84
5	6000 (UM)	0.0036		0.001	5480	3.65

TIC:	10.9660	ng/uL
TIM:	42.221	nmole/L
Total Conc.:	11.0047	ng/uL

Sample Peak Width (sec): 50   Sample Min Peak Height: 25   Sample Baseline V to V?: Y   Sample Baseline V to V pts: 3
Sample Filter: Binomial   # of Pts for Filter: 3   Sample Start Region (min): 0   Sample End Region (min): 50
Manual Baseline Start (min): 10   Manual Baseline End (min): 48
Marker Peak Width (sec): 5   Marker Min Peak Height: 200   Marker Baseline V to V?: Y   Marker Baseline V to V pts: 3
Lower Marker Selection: First Peak > 200 RFU   Upper Marker Selection: Last Peak > 200 RFU
Ladder Size (bp): 1, 100, 200, 300, 400, 500, 600, 700, 800, 900, 1000, 1200, 1500, 2000, 3000, 6000
Quantification Using: Ladder   Final Concentration (ng/uL): 0.0830   Dilution Factor: 12.0

 Copyright 2013 Advanced Analytical Technologies, Inc.

Sample: Deqiang-H1B
Well Location: D6
Created: Wednesday, October 05, 2022 11:05:58 AM

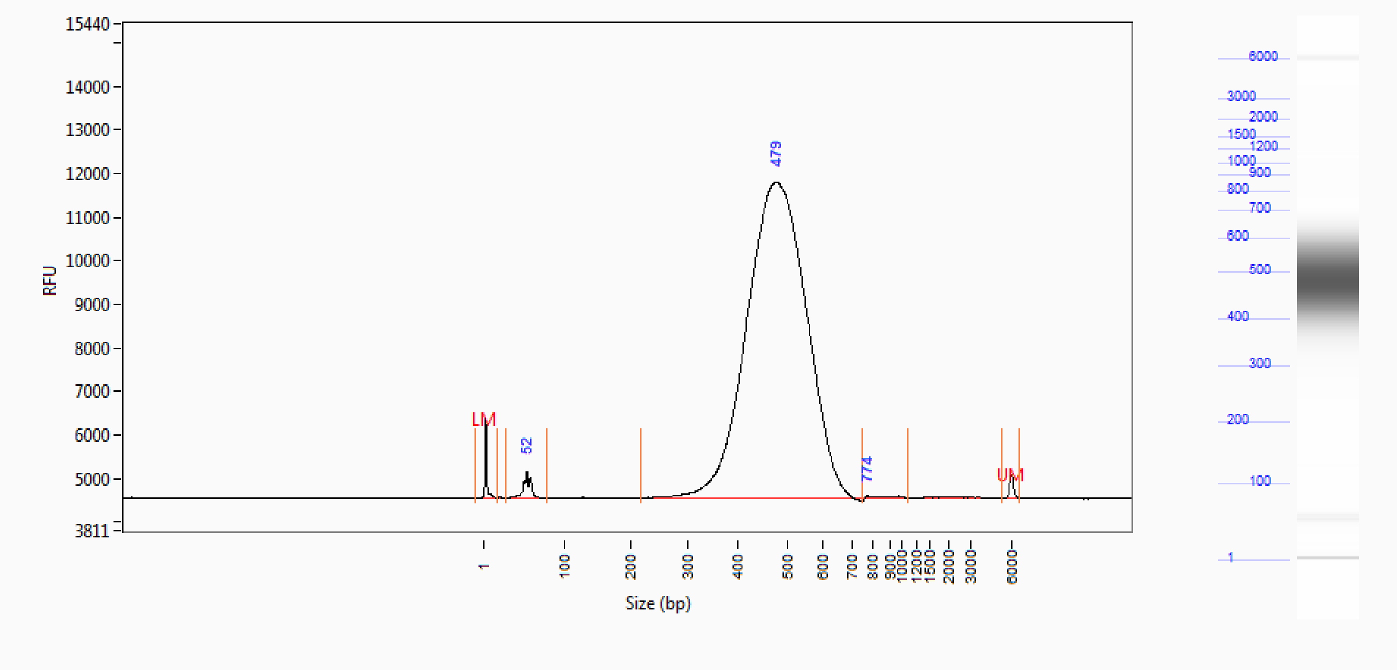

Peak	Size (bp)	Conc. (ng/uL)	Rel. Conc. %	Molarity (nmole/L)	From (bp)	CV%
1	1 (LM)	0.0133		17.353	0	186.45
2	52	0.1789	1.6	5.680	28	12.48
3	479	11.2261	98.1	38.554	218	13.23
4	774	0.0349	0.3	0.074	748	9.39
5	6000 (UM)	0.0038		0.001	5244	2.86

TIC:	11.4399	ng/uL
TIM:	44.308	nmole/L
Total Conc.:	11.5096	ng/uL

```
Sample Peak Width (sec): 50   Sample Min Peak Height: 25    Sample Baseline V to V?: Y    Sample Baseline V to V pts: 3
Sample Filter: Binomial       # of Pts for Filter: 3        Sample Start Region (min): 0  Sample End Region (min): 50
Manual Baseline Start (min): 10     Manual Baseline End (min): 48
Marker Peak Width (sec): 5    Marker Min Peak Height: 200   Marker Baseline V to V?: Y    Marker Baseline V to V pts: 3
Lower Marker Selection: First Peak > 200 RFU                Upper Marker Selection: Last Peak > 200 RFU
Ladder Size (bp): 1, 100, 200, 300, 400, 500, 600, 700, 800, 900, 1000, 1200, 1500, 2000, 3000, 6000
Quantification Using: Ladder          Final Concentration (ng/uL): 0.0830         Dilution Factor: 12.0
```

 Copyright 2013 Advanced Analytical Technologies, Inc.

Sample: Deqiang-H2B
Well Location: D7
Created: Wednesday, October 05, 2022 11:05:58 AM

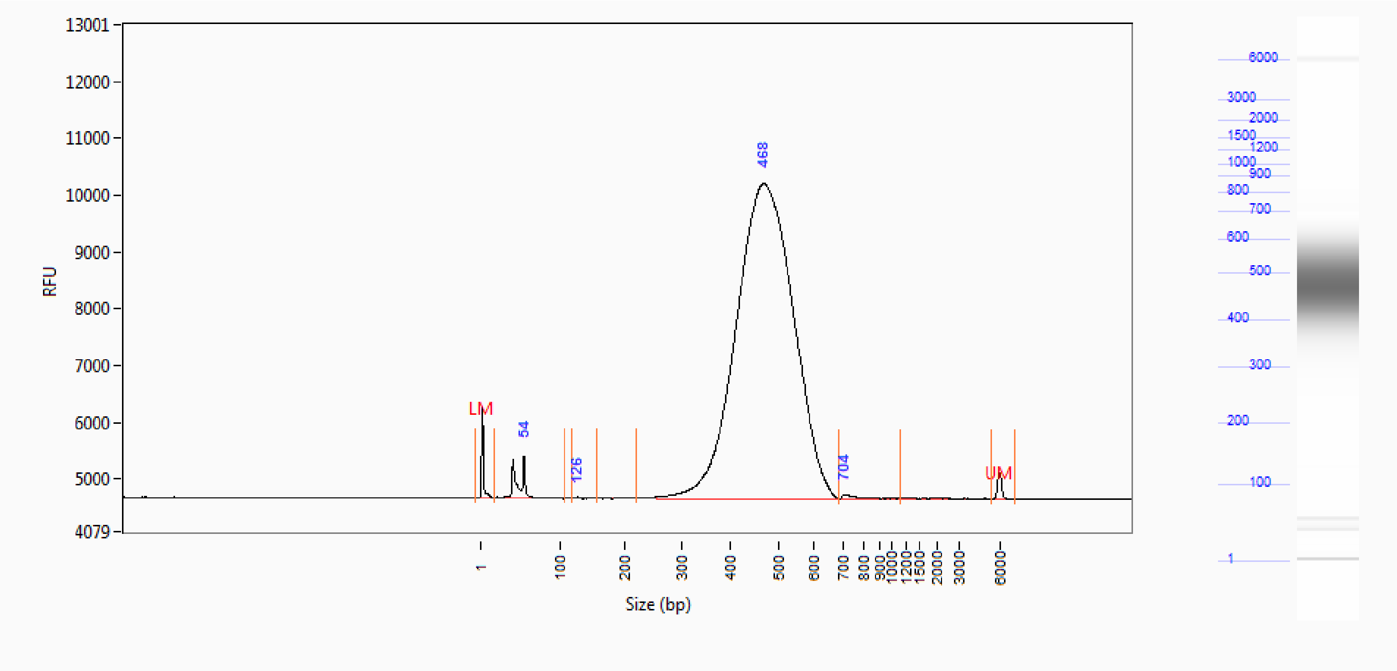

Peak	Size (bp)	Conc. (ng/uL)	Rel. Conc. %	Molarity (nmole/L)	From (bp)	CV%
1	1 (LM)	0.0133		17.353	0	156.93
2	54	0.2297	2.3	6.968	16	20.71
3	126	0.0029	0.0	0.039	118	7.38
4	468	9.7006	97.3	34.081	223	12.97
5	704	0.0335	0.3	0.078	689	12.51
6	6000 (UM)	0.0036		0.001	5410	2.75

TIC:	9.9667	ng/uL
TIM:	41.166	nmole/L
Total Conc.:	9.9900	ng/uL

Sample Peak Width (sec): 50 Sample Min Peak Height: 25 Sample Baseline V to V?: Y Sample Baseline V to V pts: 3
Sample Filter: Binomial # of Pts for Filter: 3 Sample Start Region (min): 0 Sample End Region (min): 50
Manual Baseline Start (min): 10 Manual Baseline End (min): 48
Marker Peak Width (sec): 5 Marker Min Peak Height: 200 Marker Baseline V to V?: Y Marker Baseline V to V pts: 3
Lower Marker Selection: First Peak > 200 RFU Upper Marker Selection: Last Peak > 200 RFU
Ladder Size (bp): 1, 100, 200, 300, 400, 500, 600, 700, 800, 900, 1000, 1200, 1500, 2000, 3000, 6000
Quantification Using: Ladder Final Concentration (ng/uL): 0.0830 Dilution Factor: 12.0

 Copyright 2013 Advanced Analytical Technologies, Inc.

Sample: Deqiang-H2B
Well Location: D8
Created: Wednesday, October 05, 2022 11:05:58 AM

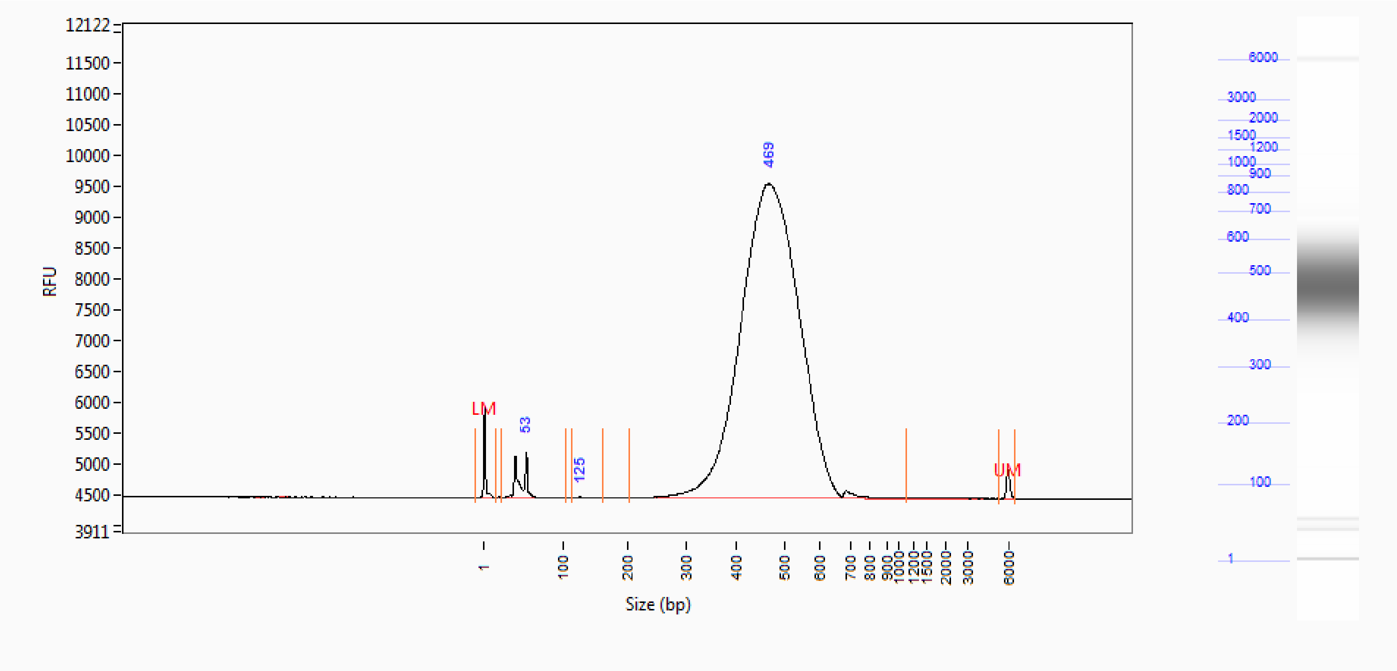

Peak	Size (bp)	Conc. (ng/uL)	Rel. Conc. %	Molarity (nmole/L)	From (bp)	CV%
1	1 (LM)	0.0133		17.353	0	144.24
2	53	0.2392	2.3	7.367	24	15.93
3	125	0.0014	0.0	0.018	113	0.62
4	469	9.9921	97.6	35.055	201	13.76
5	6000 (UM)	0.0038		0.001	5362	2.26

TIC:	10.2327	ng/uL
TIM:	42.440	nmole/L
Total Conc.:	10.2553	ng/uL

```
Sample Peak Width (sec): 50   Sample Min Peak Height: 25    Sample Baseline V to V?: Y    Sample Baseline V to V pts: 3
Sample Filter: Binomial       # of Pts for Filter: 3        Sample Start Region (min): 0  Sample End Region (min): 50
Manual Baseline Start (min): 10     Manual Baseline End (min): 48
Marker Peak Width (sec): 5    Marker Min Peak Height: 200   Marker Baseline V to V?: Y    Marker Baseline V to V pts: 3
Lower Marker Selection: First Peak > 200 RFU                Upper Marker Selection: Last Peak > 200 RFU
Ladder Size (bp): 1, 100, 200, 300, 400, 500, 600, 700, 800, 900, 1000, 1200, 1500, 2000, 3000, 6000
Quantification Using: Ladder          Final Concentration (ng/uL): 0.0830          Dilution Factor: 12.0
```

 Copyright 2013 Advanced Analytical Technologies, Inc.

Sample: SampD9
Well Location: D9
Created: Wednesday, October 05, 2022 11:05:58 AM

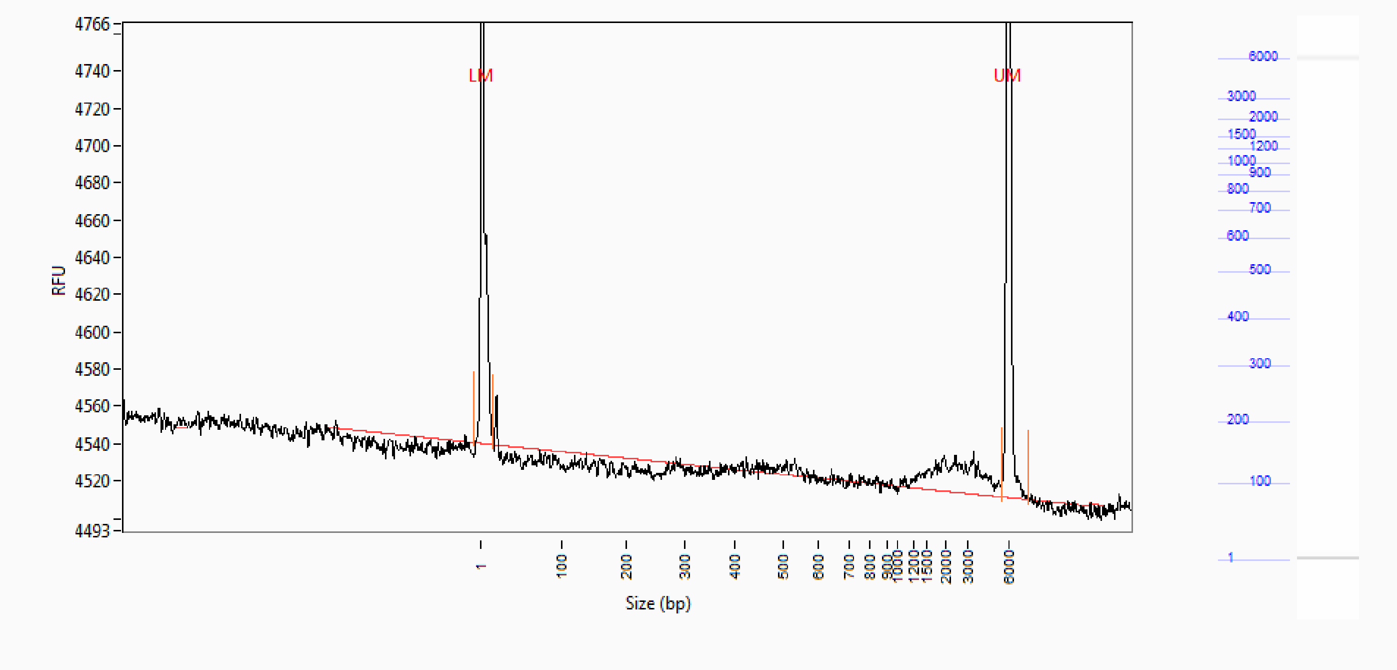

Peak	Size (bp)	Conc. (ng/uL)	Rel. Conc. %	Molarity (nmole/L)	From (bp)	CV%
1	1 (LM)	0.0133		17.353	0	218.34
2	6000 (UM)	0.0038		0.001	5480	2.97

TIC:	0.0000	ng/uL
TIM:	0.000	nmole/L
Total Conc.:	0.0228	ng/uL

Sample Peak Width (sec): 50 Sample Min Peak Height: 25 Sample Baseline V to V?: Y Sample Baseline V to V pts: 3
Sample Filter: Binomial # of Pts for Filter: 3 Sample Start Region (min): 0 Sample End Region (min): 50
Manual Baseline Start (min): 10 Manual Baseline End (min): 48
Marker Peak Width (sec): 5 Marker Min Peak Height: 200 Marker Baseline V to V?: Y Marker Baseline V to V pts: 3
Lower Marker Selection: First Peak > 200 RFU Upper Marker Selection: Last Peak > 200 RFU
Ladder Size (bp): 1, 100, 200, 300, 400, 500, 600, 700, 800, 900, 1000, 1200, 1500, 2000, 3000, 6000
Quantification Using: Ladder Final Concentration (ng/uL): 0.0830 Dilution Factor: 12.0

 Copyright 2013 Advanced Analytical Technologies, Inc.

Sample: SampD10
Well Location: D10
Created: Wednesday, October 05, 2022 11:05:58 AM

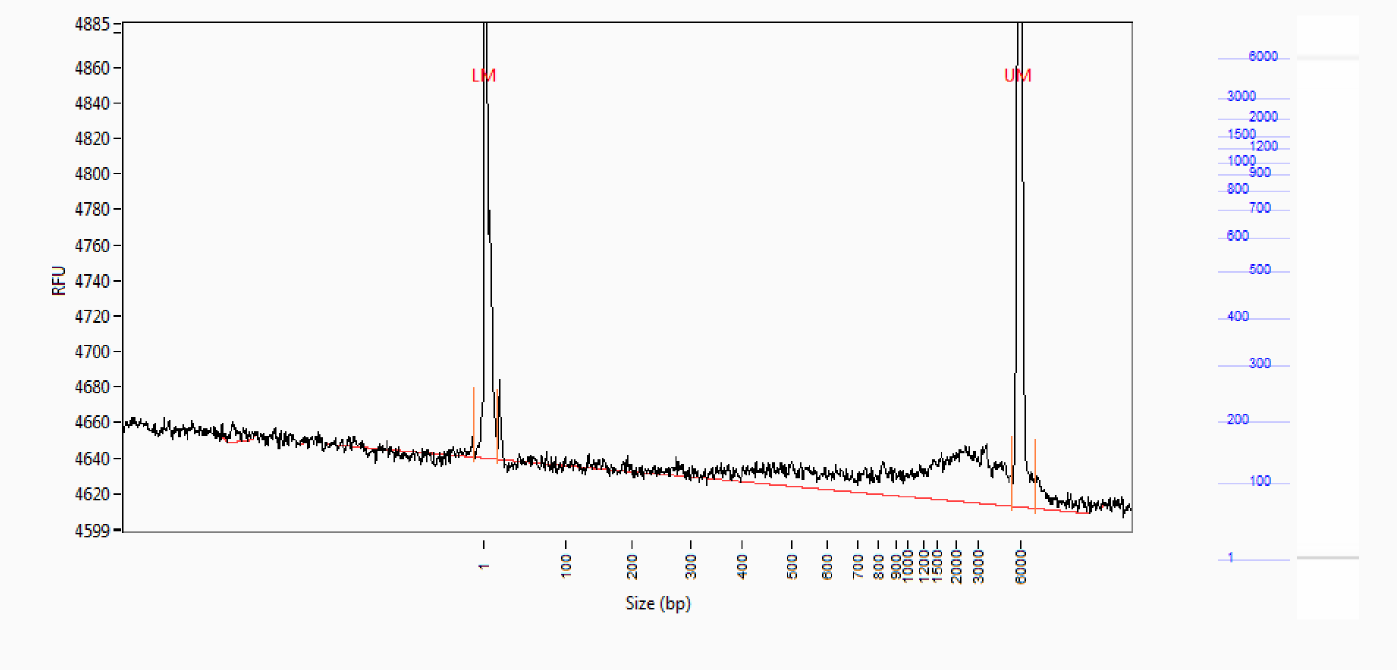

Peak	Size (bp)	Conc. (ng/uL)	Rel. Conc. %	Molarity (nmole/L)	From (bp)	CV%
1	1 (LM)	0.0133		17.353	0	194.50
2	6000 (UM)	0.0035		0.001	5504	3.77

TIC:	0.0000	ng/uL
TIM:	0.000	nmole/L
Total Conc.:	0.0629	ng/uL

```
Sample Peak Width (sec): 50   Sample Min Peak Height: 25    Sample Baseline V to V?: Y    Sample Baseline V to V pts: 3
Sample Filter: Binomial       # of Pts for Filter: 3        Sample Start Region (min): 0  Sample End Region (min): 50
Manual Baseline Start (min): 10     Manual Baseline End (min): 48
Marker Peak Width (sec): 5    Marker Min Peak Height: 200   Marker Baseline V to V?: Y    Marker Baseline V to V pts: 3
Lower Marker Selection: First Peak > 200 RFU                Upper Marker Selection: Last Peak > 200 RFU
Ladder Size (bp): 1, 100, 200, 300, 400, 500, 600, 700, 800, 900, 1000, 1200, 1500, 2000, 3000, 6000
Quantification Using: Ladder          Final Concentration (ng/uL): 0.0830          Dilution Factor: 12.0
```

 Copyright 2013 Advanced Analytical Technologies, Inc.

Sample: SampD11
Well Location: D11
Created: Wednesday, October 05, 2022 11:05:58 AM

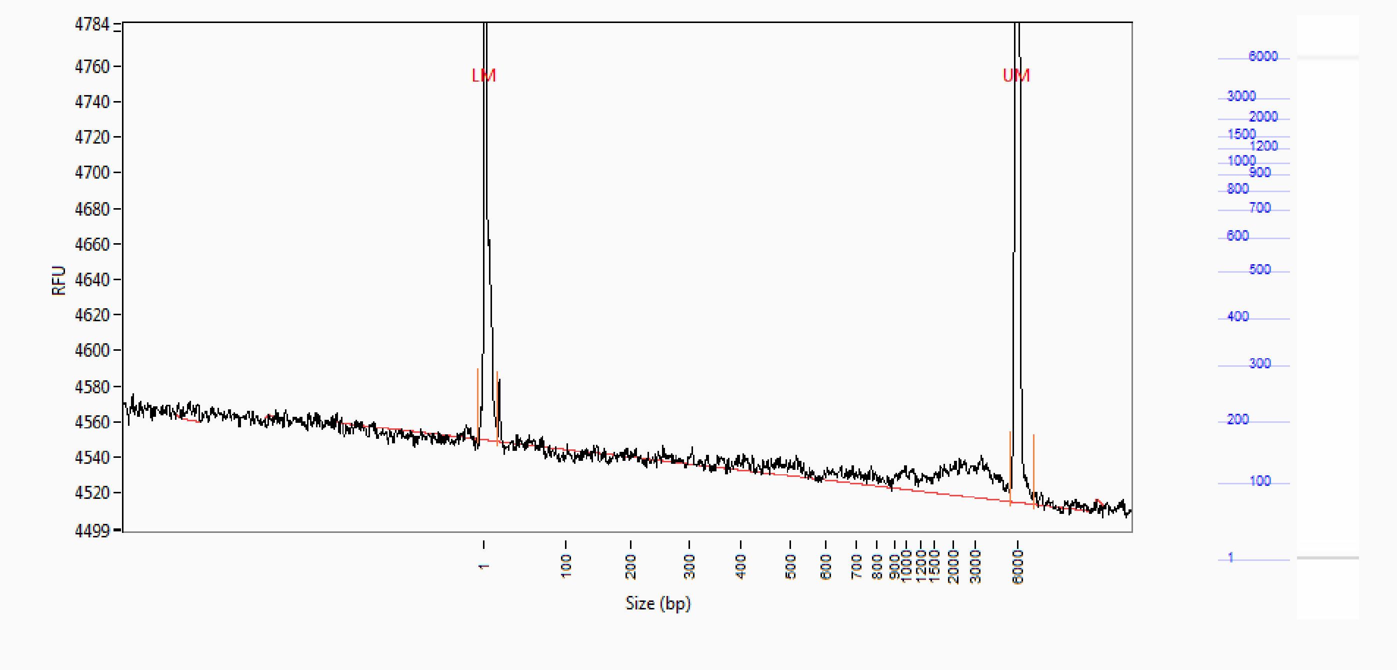

Peak	Size (bp)	Conc. (ng/uL)	Rel. Conc. %	Molarity (nmole/L)	From (bp)	CV%
1	1 (LM)	0.0133		17.353	0	185.88
2	6000 (UM)	0.0034		0.001	5480	3.12

TIC:	0.0000	ng/uL
TIM:	0.000	nmole/L
Total Conc.:	0.0424	ng/uL

```
Sample Peak Width (sec): 50   Sample Min Peak Height: 25    Sample Baseline V to V?: Y    Sample Baseline V to V pts: 3
Sample Filter: Binomial       # of Pts for Filter: 3        Sample Start Region (min): 0  Sample End Region (min): 50
Manual Baseline Start (min): 10     Manual Baseline End (min): 48
Marker Peak Width (sec): 5    Marker Min Peak Height: 200   Marker Baseline V to V?: Y    Marker Baseline V to V pts: 3
Lower Marker Selection: First Peak > 200 RFU                Upper Marker Selection: Last Peak > 200 RFU
Ladder Size (bp): 1, 100, 200, 300, 400, 500, 600, 700, 800, 900, 1000, 1200, 1500, 2000, 3000, 6000
Quantification Using: Ladder          Final Concentration (ng/uL): 0.0830          Dilution Factor: 12.0
```

 Copyright 2013 Advanced Analytical Technologies, Inc.

Sample: ladder
Well Location: D12
Created: Wednesday, October 05, 2022 11:05:58 AM

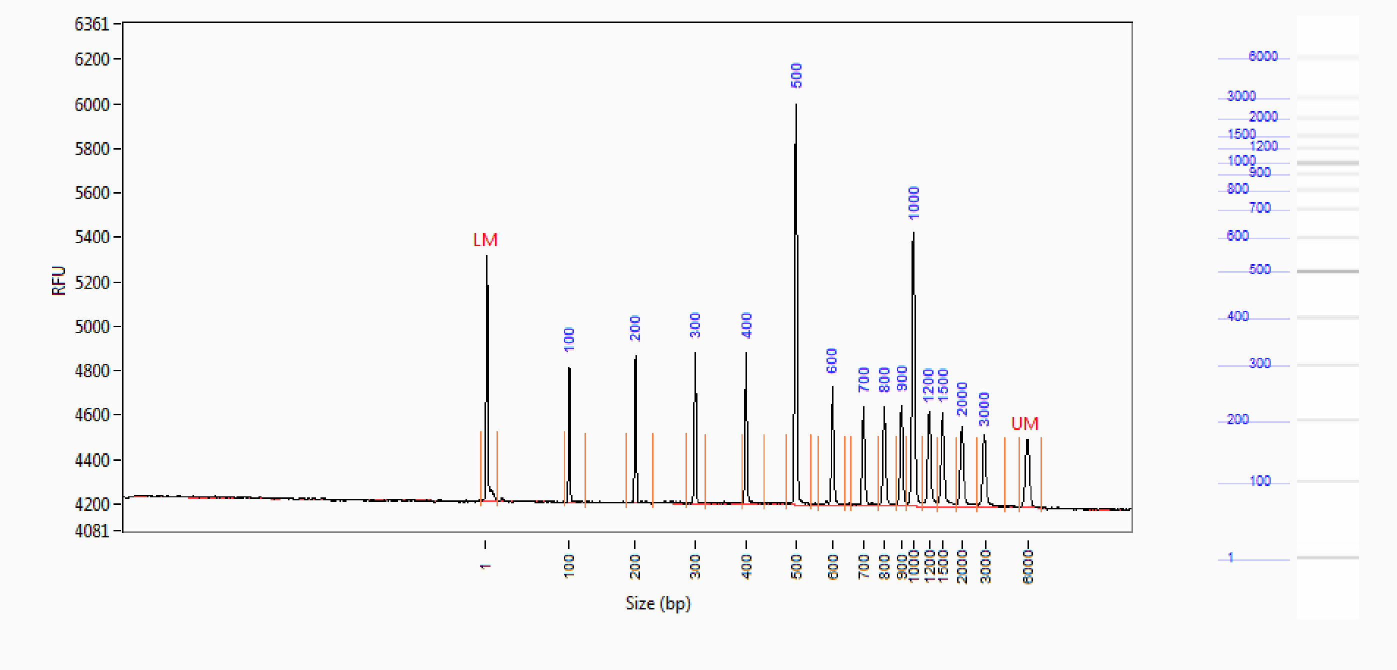

Peak	Size (bp)	Conc. (ng/uL)	Rel. Conc. %	Molarity (nmole/L)	From (bp)	CV%
1	1 (LM)	0.0133		17.353	0	143.96
2	100	0.0595	6.1	0.977	96	2.36
3	200	0.0607	6.3	0.499	186	1.56
4	300	0.0626	6.5	0.343	287	1.05
5	400	0.0674	6.9	0.277	393	1.34
6	500	0.1772	18.3	0.583	480	0.84
7	600	0.0587	6.1	0.161	561	1.30
8	700	0.0500	5.1	0.117	661	1.78
9	800	0.0500	5.1	0.103	771	1.48
10	900	0.0486	5.0	0.089	869	1.08
11	1000	0.1386	14.3	0.228	942	1.80
12	1200	0.0520	5.4	0.071	1109	3.39
13	1500	0.0540	5.6	0.059	1393	4.77
14	2000	0.0479	4.9	0.039	1849	6.64
15	3000	0.0431	4.4	0.024	2697	7.48
16	6000 (UM)	0.0035		0.001	5480	2.83

TIC:	0.9704	ng/uL
TIM:	3.573	nmole/L
Total Conc.:	0.9960	ng/uL

```
Sample Peak Width (sec): 10   Sample Min Peak Height: 100   Sample Baseline V to V?: Y    Sample Baseline V to V pts: 3
Sample Filter: Binomial       # of Pts for Filter: 3        Sample Start Region (min): 0  Sample End Region (min): 50
Manual Baseline Start (min): 10     Manual Baseline End (min): 48
Marker Peak Width (sec): 5    Marker Min Peak Height: 200   Marker Baseline V to V?: Y    Marker Baseline V to V pts: 3
Lower Marker Selection: First Peak > 200 RFU                Upper Marker Selection: Last Peak > 200 RFU
Ladder Size (bp): 1, 100, 200, 300, 400, 500, 600, 700, 800, 900, 1000, 1200, 1500, 2000, 3000, 6000
Quantification Using: Ladder          Final Concentration (ng/uL): 0.0830          Dilution Factor: 12.0
```


Copyright 2013 Advanced Analytical Technologies, Inc.

Sample: ladder
Well Location: D12
Created: Wednesday, October 05, 2022 11:05:58 AM
Fit Type: Point to Point

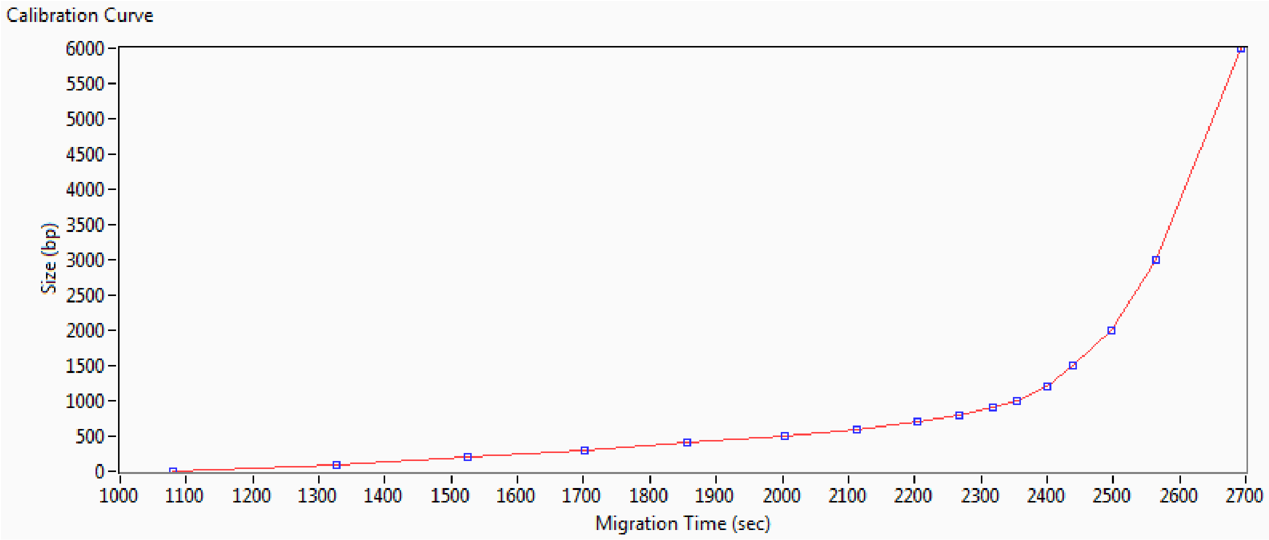

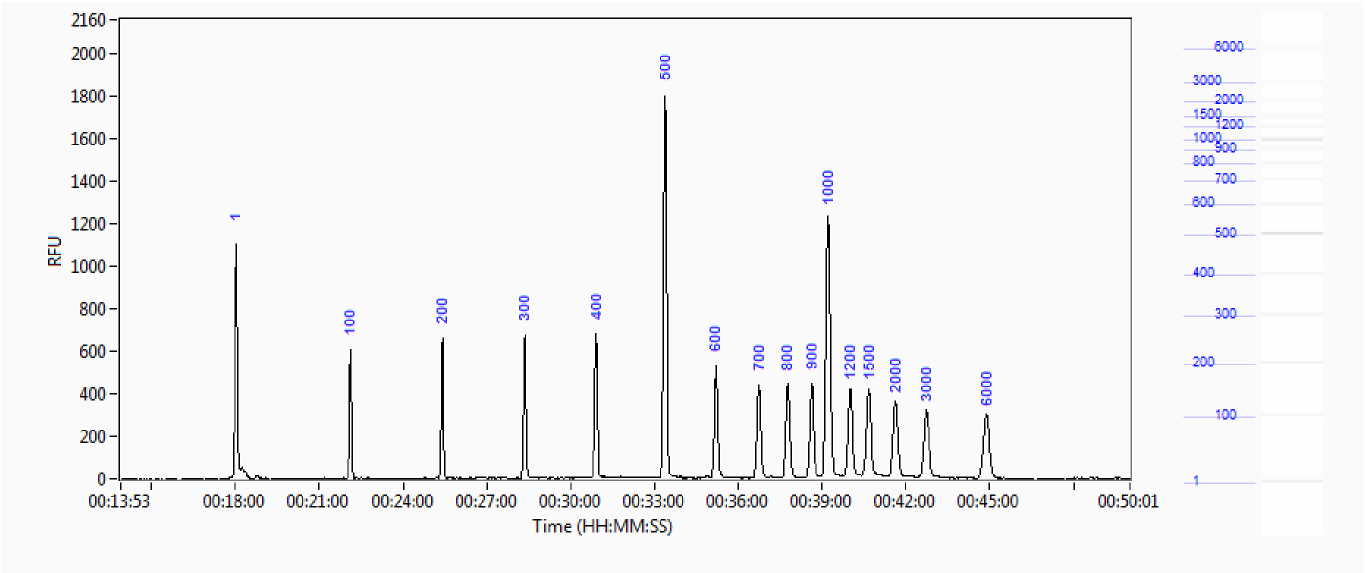

 Copyright 2013 Advanced Analytical Technologies, Inc.
